# Structure-Dependent Water Solubility and Receptor Properties of C_3_‑Symmetric Dendrimers Bearing Sumanene or Triphenylene Cores

**DOI:** 10.1021/acsorginorgau.5c00048

**Published:** 2025-07-22

**Authors:** Stanisław Kulczyk, Yumi Yakiyama, Mariola Koszytkowska-Stawińska, Hidehiro Sakurai, Artur Kasprzak

**Affiliations:** † Faculty of Chemistry, 49566 Warsaw University of Technology, Noakowskiego Street 3, 00-664 Warsaw, Poland; ‡ Division of Applied Chemistry, Graduate School of Engineering, The University of Osaka, 2-1 Yamadaoka, Suita, 565-0871 Osaka, Japan; § Innovative Catalysis Science Division, Institute for Open and Transdisciplinary Research Initiatives (ICS-OTRI), The University of Osaka, Suita, Osaka 565-0871, Japan

**Keywords:** sumanene, molecular receptor, water solubility, cation detection, aluminum, indium, gallium

## Abstract

Four water-soluble dendrimers with sumanene or triphenylene cores were obtained with the intended application as molecular receptors. Their water solubilities were found to vary from 46 to 820 μM. Solubility values strongly correlated with free energy of dimerization values calculated using umbrella sampling method (a molecular dynamics-based method.). Moreover, it was found that the fluorescence of two compounds was selectively quenched in the presence of group 13 cations (Al^3+^, Ga^3+^, and In^3+^) in water with nanomolar limit of detection values. Structures of the formed complexes were proposed based on cation-binding site studies by ^13^C­{^1^H} NMR spectroscopy. Performance of a selected compound in the analysis of real-life water samples was examined. It was found that the compound was capable of detecting group 13 cation contamination in surface water samples when the organic substance content was low. The obtained compounds are the first known water-soluble sumanene derivatives and the first sumanene derivatives capable of group 13 cation detection.

## Graphical abstract

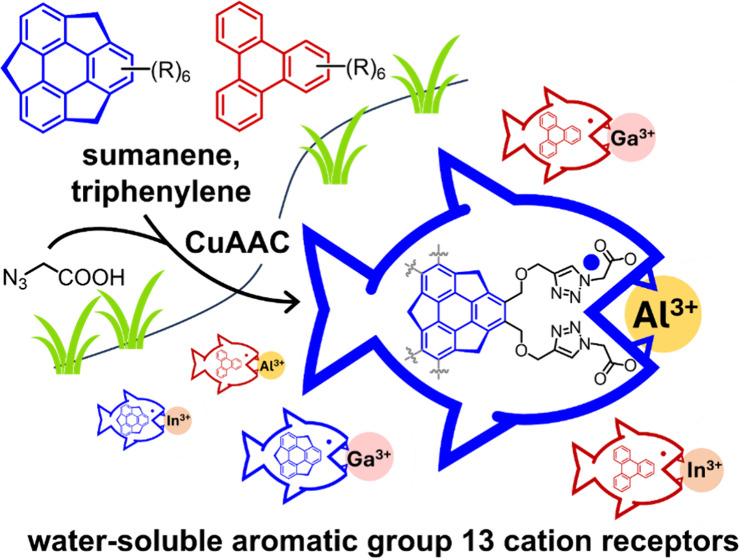

## Introduction

Design of organic receptors that are able to selectively detect metal cations in aqueous solutions has drawn significant attention from the scientific community for almost half a century. Fluorescent receptors constitute one of the most important class of optical receptors.^1–3^ The molecular recognition process with such receptors most commonly includes noncovalent interactions with analytes (metal cations) with the participation of receptors’ structural motifs, such as oxygen or nitrogen containing groups. The polyaromatic skeleton is crucial in terms of providing attractive fluorescent properties of optical receptors, enabling effective tracking of the fluorescence boosting or quenching behaviors. There are many possibilities regarding the choice of such a π-conjugated aromatic motif for a given application. Most commonly, highly emissive π-conjugated planar molecules are used for such purposes, including pyrene, perylene, or triphenylene.^4–7^

Attractive possibilities in terms of fluorescent aromatic compound design have been initiated with the discovery of bowl-shaped molecules.^8–10^ Sumanene is a flagship example belonging to this class of compounds (Figure 1).^11–14^ It is a C_60_ fullerene fragment featuring the bowl depth of 1.11 Å.^15^ Since its discovery, sumanene has received constantly increasing attention of the scientific community due to attractive possibilities toward application in light-emitting devices, supramolecular frameworks, sensors, and dielectric materials.^16–19^ Notably, apart from introducing fluorescent properties to the receptor, the polyaromatic skeleton itself might also take part in the molecular recognition process by means of cation-π interaction forces. In terms of sumanene molecule and its derivatives, such an application concept is a new research idea; the properties of sumanene congeners toward the recognition of cesium,^20–23^ lithium,^24^ or silver cations^25^ were reported recently. Due to the presence of curvature in sumanene receptor structures, their photophysical and receptor properties are tuned in comparison to their planar analogues.

**Figure 1:**
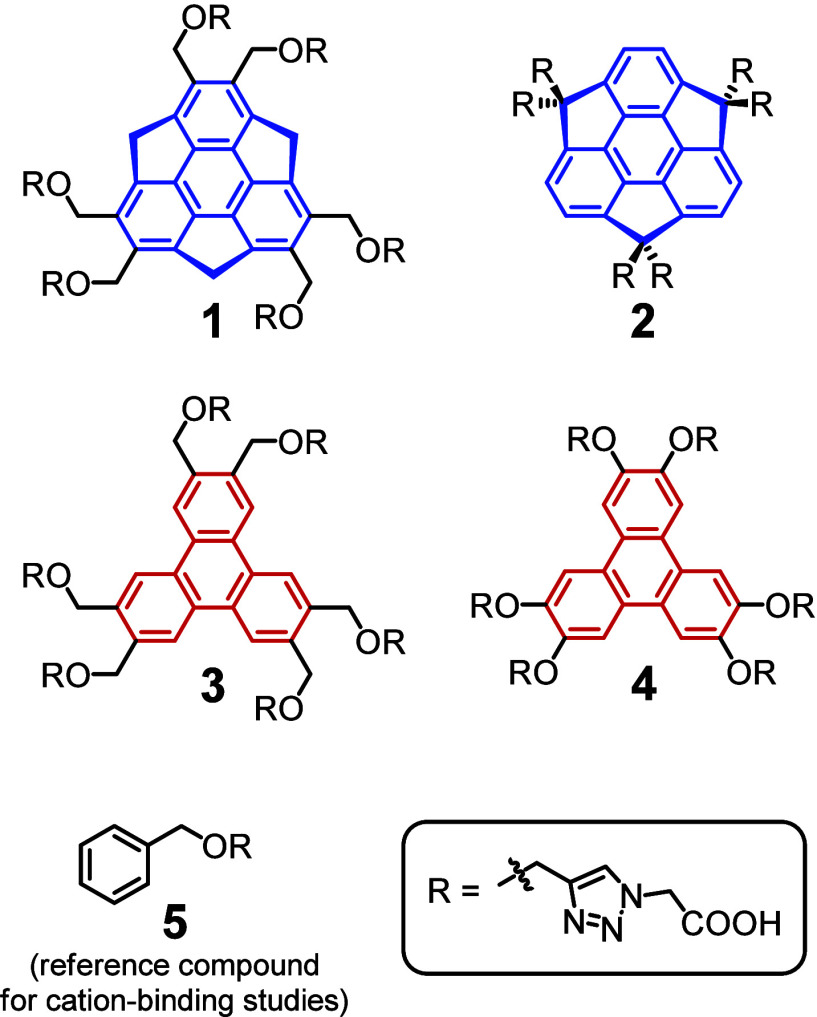
Sumanene (blue) and triphenylene (red) cores in the synthesized compounds **1**–**** **5**.

The concept of designing water-soluble metal cation receptors featuring an aromatic compound motif has been one of the most attractive yet difficult research endeavors. This results from the need to design a structurally sophisticated aromatic receptor molecule featuring both acceptable water solubility profile and selective recognition feature in aqueous solution, which is a demanding environment in terms of molecular recognition process of metal cations.^26^ Nevertheless, important achievements in this field have been reported in the field of water-soluble molecular receptors and supramolecular chemistry in general, such as the design of water-soluble organic cages^27–29^ or macrocycles, including those able to act as molecular receptors.^30–34^ Within the bowl-shaped aromatic molecule family, there are recent reports on the design of corannulene derivatives featuring water solubility profiles satisfactory for biological applications.^35,36^

Herein, we report the design of four water-soluble dendritic molecules **1–4** featuring the sumanene or triphenylene structural motif (Figure 1). We found that the observed water solubility reported for the first time in sumanene chemistry was dependent on the interactions between the aromatic motifs in the solid phase. We discovered that molecules **1** and **3** feature unexpected property of selectively recognizing group 13 cations, namely, Al^3+^, Ga^3+^, and In^3+^. The interactions were not only characterized by satisfactory binding parameters, a selected receptor (**1**) could also be applied for the analysis of real-life water samples contaminated with group 13 cations.

## Results and Discussion

### Molecular Design, Synthesis and Spectroscopic Properties

Four potential water-soluble cation receptors (**1–4**) with sumanene and triphenylene cores were designed and synthesized (Figure 1). The compounds were dendrimers with hydrophilic arms attached to aromatic cores. Polar, hydrophilic arms provided them with water solubility and cation recognition capabilities. Aromatic cores provided them with near-UV absorption and fluorescence properties. Out of the synthesized compounds, **1** and **2** had bowl-shaped sumanene cores and compounds **3** and **4** had triphenylene cores. In **1**, hydrophilic arms were attached to the sumanene core in the aromatic positions of sumanene, and in **2**, the arms were attached in the benzylic positions. Compound **3** was an analogue of **1** with a triphenylene core rather than a sumanene core. Compound **4** was an analogue of **3** with the difference being one less methylene bridge in each arm. In addition, **5** (Figure 1) was obtained as a reference compound to determine the cation-binding mechanism of the above. Overall, **1**–**** **5** constitute a library of hydrophilic dendrimers of high structural similarity.

Refer to the "Methods" section at the end of the main text and Supporting Information (SI) for experimental details on the synthesis of dendrimers **1–5** and synthetic intermediates, as well on their characterization data (1D, 2D and pseudo 2D NMR, and HRMS). Briefly, the synthesis of each compound involved two stages: (a) synthesis of a propargylated precursor and (b) copper-catalyzed 1,3-dipolar cycloaddition (CuAAC) reaction (click chemistry approach) between the precursor and azidoacetic acid. The first stage was realized differently for each compound (Figure 2). Precursor **7** was synthesized from hexakis­(bromomethyl)­sumanene (**6**)^37^ in 82% yield by alkoxylation with propargyl alcohol in the presence of dibutyltin­(IV) oxide. Precursor **8** was obtained in 88% yield by alkylation of pristine sumanene (**9**) with propargyl bromide under PTC conditions.^38^ Precursor **12** was synthesized from ester **10** ^39^ by means of reduction with LiAlH_4_ (56% yield) and alkylation of the obtained alcohol (**11**) with propargyl bromide in DMSO in the presence of KOH (32% yield).^40^ Precursor **14** was synthesized from hexahydroxytriphenylene (**13**) by means of alkylation with propargyl bromide in the presence of K_2_CO_3_ in 82% yield. Last, precursor **16** was synthesized from benzylic alcohol (**15**) by alkylation with propargyl bromide in DMSO in the presence of KOH (81% yield).^40^

**Figure 2:**
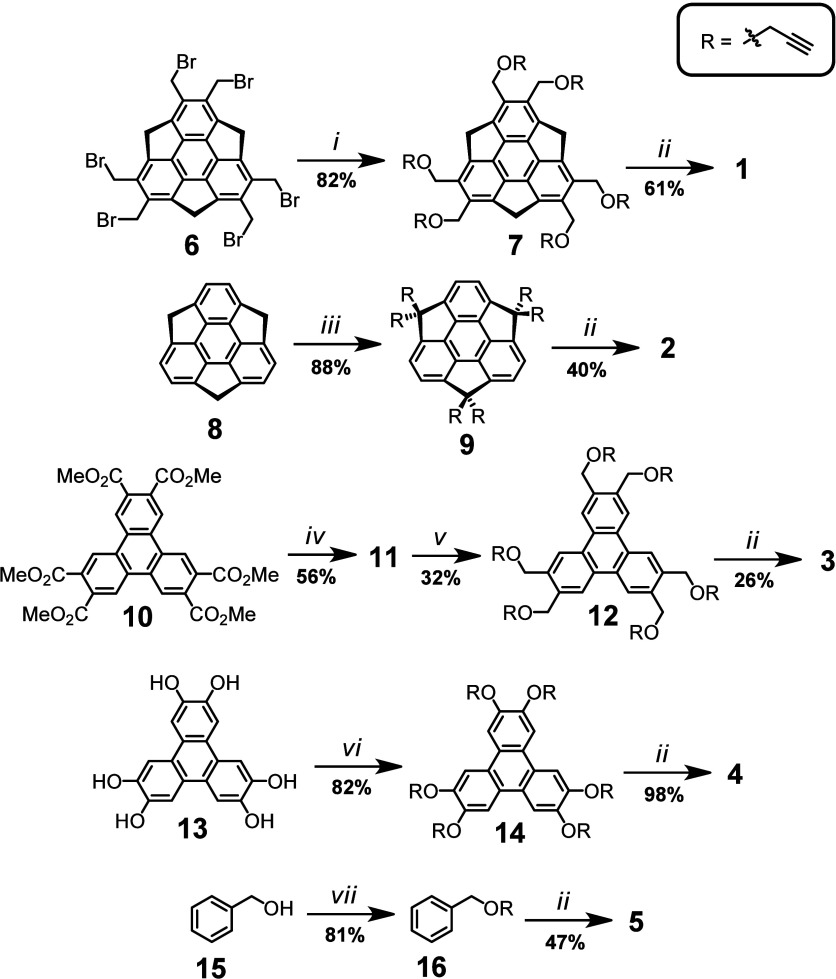
Synthesis of compounds **1–5** and their precursors **6**–**** **16**. Reagents and reaction conditions: (i) propargyl alcohol, SnO­(*n*-Bu)_2_, 80 °C, 3 h; (ii) azidoacetic acid, copper­(I) thiophene-2-carboxylate (CuTC), Et_3_N, DMSO, 80 °C (microwave conditions), 20 min; (iii) propargyl bromide, 30% NaOH (*aq*), THF, tetrabutylammonium bromide (TBAB), room temperature (RT), 48 h; (iv) LiAlH_4_, THF, 0 → 80 °C (microwave conditions), 2 h; (v) propargyl bromide, KOH, DMSO, RT, 3 h; (vi) propargyl bromide, K_2_CO_3_, DMF, RT, 8 days. (vii) propargyl bromide, KOH, DMSO, RT, 16 h.

Propargylated precursors **7**, **9**, **12**, **14**, and **16** were subjected to CuAAC reactions with azidoacetic acid to afford dendrimeric triazoles **1**–**** **5**. The cycloaddition reactions were conducted in DMSO in the presence of copper­(I) thiophene-2-carboxylate (CuTC)^41^ and triethylamine (TEA) in 80 °C under microwave irradiation over the course of 20 min. Products **1**–**** **4** were isolated by means of dissolving the reaction mixture in sodium carbonate (5%, *aq*), washing the water phase with ethyl acetate, precipitating the product with HCl (*aq*), separating the product on a membrane filter, and washing it with deionized water. Because of its high solubility, product **5** was isolated differently, namely, by extraction and recrystallization from water. Crude products **1**–**** **4** were found to be contaminated by Cu^2+^ and thus were subjected to Cu^2+^ removal procedure similar to the one described by Jansa et al.^42^ The final products **1**–**** **5** were obtained as powders in varying yields (from 26 to 98%). They were free from Cu^2+^ contamination [<0.5 mol % Cu^2+^, measured by induced coupling plasma-atomic emission spectroscopy (ICP-AES)].

Compounds **1–16** were characterized using HRMS, as well as ^1^H and ^13^C­{^1^H} NMR spectroscopies. Signals in 1D NMR spectra were assigned using ^1^H–^13^C HMBC spectroscopy. The presence of 1,2,3-triazole skeletons in compound **1** was further confirmed by ^1^H–^15^N HMBC spectroscopy, and the structure of **9** was additionally confirmed by single-crystal X-ray diffraction. ^1^H DOSY NMR experiments confirmed that the samples were composed of single compounds with hydrodynamic radii (*r*_H,solv_) given below (Table 1, where *r*_H,solv_ values were calculated using the unmodified Stokes–Einstein equation). Radii of **1**, **3,** and **4** were similar (about 1.30 nm). Radius of **2** was smaller (1.15 nm), which could be explained by its more spherical shape.^43,44^ The small model compound **5** had a radius of 0.50 nm. Overall, the measured hydrodynamic radii were similar to the radii reported for similar compounds.^45–47^

**Table 1:** Chosen Physical Properties of Compounds **1**–**** **5** ^a^

compound	*r*_H,solv_ [nm]	*λ*_abs_ [nm]	*λ*_exc_ [nm]	*λ*_em_ [nm]	*ε* [M^–1^·cm^–1^]	*Φ* [%]
**1**	1.30	290	290	390	68,500	6.7
**2**	1.15	278	279	375	43,300	7.7
**3**	1.30	275	271	376	58,400	9.5
**4**	1.30	269	266	383	62,200	14.5
**5**	0.50	ND	ND	ND	ND	ND

a *r*_H,solv_ – Hydrodynamic radius (DMSO, 30 °C, 10 mM concentration), *λ*_abs_ – absorption maximum, *λ*_exc_ – excitation maximum, *λ*_em_ – emission maximum, *ε* – molar absorption coefficient, and *Φ* – quantum yield. ND – not determined.

Optical properties of **1**–**4** in H_2_O are summarized in Table 1 and are presented in Figure 3. All of the studied compounds featured singular sharp absorption maxima (*λ*_abs_) at wavelengths from 269 nm (**4**) to 290 nm (**1**). These maxima could be attributed to π–π* transitions in their aromatic cores^48^ and were characterized by molar absorption coefficient (*ε*) values from 43,300 M^–1^·cm^–1^­(**2**) to 68,500 M^–1^·cm^–1^­(**1**). Values of excitation maxima (*λ*_exc_) were very similar to the values of absorption maxima (λ_abs_) in each case. Emission maxima (*λ*_em_) were found at wavelengths from 375 nm (**2**) to 390 nm (**2**). Stokes shifts were large: from 96 nm (**2**) to 117 nm (**4**). Quantum yields (*Φ*) varied from 6.7% (**1**) to 14.5% (**4**). Exchange of a sumanene core (**1**) to a triphenylene core (**3**) resulted in an increase of the quantum yield (from 6.7 to 9.5%) at the expense of hypsochromic shift of absorption (by 15 nm) and emission (by 14 nm) maxima.

**Figure 3:**
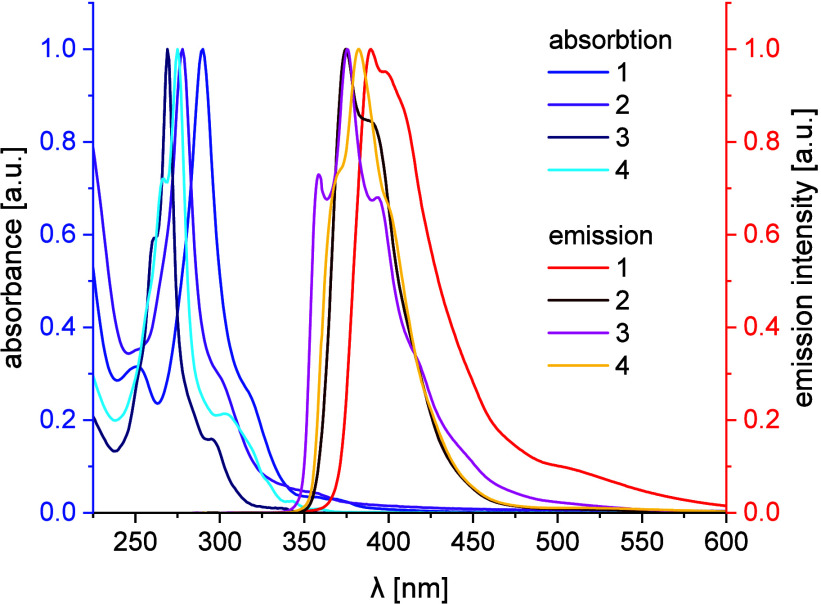
Absorption and emission spectra of compounds **1–4**. Solvent: H_2_O, concentration: 10^–5^ M, excitation wavelengths: **1**: 290 nm, **2**: 279 nm, **3**: 271 nm, **4**: 266 nm.

### Solubility

Having measured the absorption coefficients of **1**–**** **4**, it was possible to determine their water solubility. The solubility was determined by measuring the absorbance of samples prepared from saturated solutions of each compound (for more details, see SI). All compounds **1–4** were found to be soluble in water to different extents. Interestingly, the solubilities of compounds **1**–**** **4** varied significantly (Table 2). The results obtained were unexpected as they did not correlate with lipophilicity or size of the molecules.

**Table 2:** Solubility and Predicted Free Energies of Dimerization in Water (Δ*G°*_dimerization_) of Compounds **1**–**** **4**

compound	solubility [μM]	Δ*G°*_dimerization_ [kJ/mol]
**1**	149	–60.1 ± 3.3
**2**	820	–37.2 ± 1.6
**3**	299	–52.9 ± 2.0
**4**	46	–74.7 ± 2.1

Dimerization is a type of aggregation process previously studied in the context of predicting solubility.^49^ Therefore, it was hypothesized that the solubility of **1–4** could correlate with the dimerization energy of these molecules. Free energy of dimerization was calculated using the umbrella sampling method.^50,51^ This molecular dynamics-based method allows us to accurately predict the free energy of an association process under dynamic conditions and in the presence of an explicit solvent. In this study, the association of two molecules of each compound in water at 298 K was considered. The computed free energy was in excellent correlation with the observed solubility with the correlation coefficient as high as *R* ^2^ = 0.990 (Figure 4, Table 2). This result suggested that dimerization energy calculations could find use in quantitative solubility prediction models.

**Figure 4:**
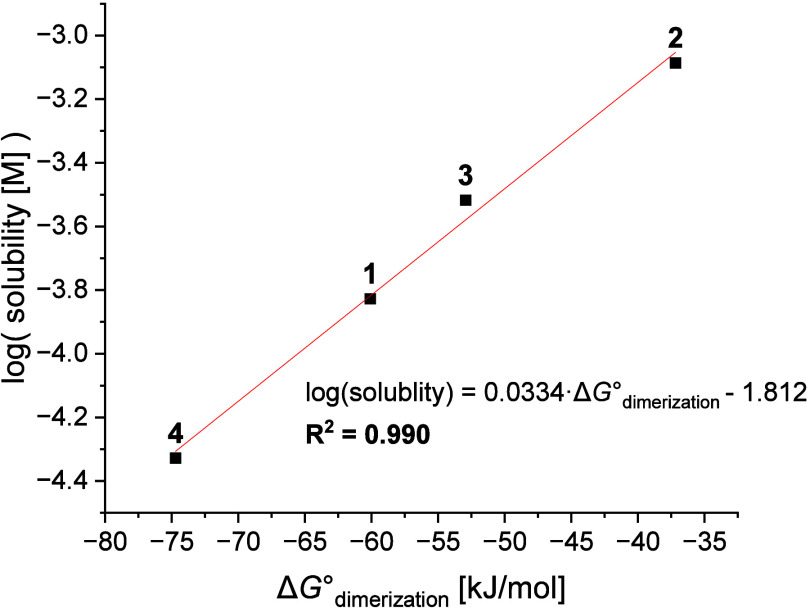
Measured logarithm of solubility of **1**–**** **4** as a function of their predicted free energy of dimeriztaion (Δ*G*°_dimerization_). Experimental and predicted values are highly correlated (*R* ^2^ = 0.990).

### Receptor Properties

Dendrimers **1**–**** **4** were investigated as potential fluorescent cation receptors. A preliminary assay involved measurements of fluorescence of compounds **1**–**** **4** in the presence of 18 different cations. Measurements were conducted in water in the presence of 1 mM pH 5.0 AcOH/Tris buffer. The acidic buffer was used to limit the hydrolysis of acidic cations.^52^ Concentration of the receptor compounds was 0.1 μM, and the concentration of the metal ions was 5.0 μM. Fluorescence quenching was observed in all cases. While **2** and **4** were nonselective (SI), **1** and **3** displayed selective quenching in the presence of group 13 cations, namely, Al^3+^, Ga^3+^, and In^3+^ (Figure 5). Interestingly, Sc^3+^ cation, the size of which (89 ppm) was between those of Ga^3+^ (76 ppm) and In^3+^ (94 ppm) caused only a moderate response.^53^ In 2015, Goesten et al. postulated that group 13 cations should display higher affinity to small-atom ligands than their d-block counterparts, such as Sc^3+^, because of their unique electronic properties.^54^ Our experimental finding seems to be in agreement with this prediction.

**Figure 5:**
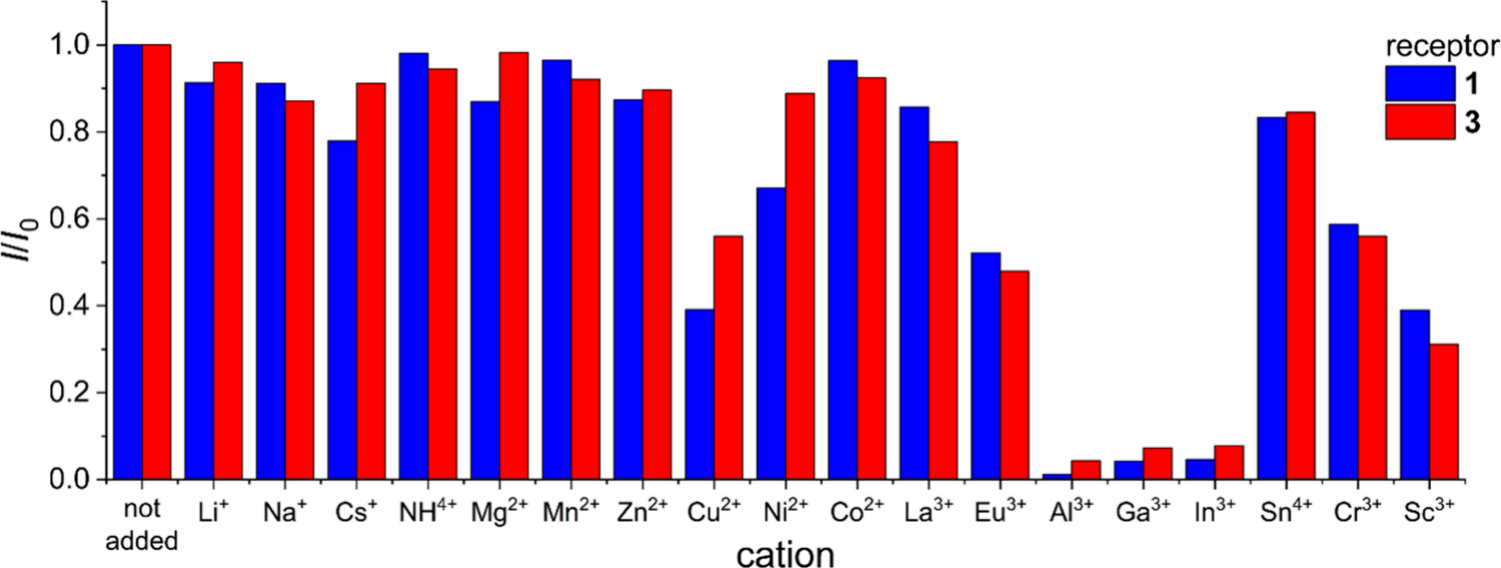
Fluorescence intensity of **1** and **3** in the presence of various cations. Fluorescence was selectively quenched in the presence of group 13 cations (Al^3+^, Ga^3+^, and In^3+^). Solvent: water; receptor concentration: 0.1 μM; cation concentration: 5 μM, 1 mM of pH 5.0 AcOH/Tris buffer added.

A literature survey was conducted to compare **1** and **3** to other fluorescent receptors for the detection of group 13 cations in aqueous media (see the SI for methodology and results). From the structural point of view, receptors based on Schiff bases were by far most commonly reported.^55–57^ The use of a carboxylic group as a cation-binding motif, as in **1** and **3**, is therefore novel. The literature reports on In^3+^-selective receptors are sparse; to the best of our knowledge, only seven publications featuring such receptors were published so far.^58–64^ Furthermore, **1** and **3** appear to be the first ever reported receptors with the ability to selectively detect all group 13 cations: Al^3+^, Ga^3+^, and In^3+^.

Detailed fluorometric titrations of **1**, **3,** and **4** with group 13 cations were conducted to further assess their cation-binding properties (see the SI for a full description). The obtained quenching curves were found to fit to a modified double logarithmic Stern–Volmer equation.^65–67^ Fitting results are summarized in Table 3. Based on these results, we concluded that the stoichiometry of receptor-ion complexes was approximately 1:2 in each case (2 cations bound to 1 receptor). LODs (limits of detection) were also calculated as 3σ of the fitting. They were found to be in the nanomolar range, namely, from 90 to 370 nM.

**Table 3:** Modified Stern–Volmer Parameters of Quenching of **1**, **3,** and **4** by Group 13 Cations^a^

compound	cation	LOD [nM]	*n*	log (*K*_SV_)
**1**	Al^3+^	240	1.65	9.93
	Ga^3+^	370	1.40	8.24
	In^3+^	90	1.94	12.12
**3**	Al^3+^	170	2.58	15.41
**4**	Al^3+^	110	2.87	17.63

a LOD – limit of detection, n – average number of cations binding to one receptor, and *K*_SV_ – modified Stern–Volmer constant.

Cation-binding mechanism of the obtained compounds was further elucidated using ^13^C­{^1^H} NMR spectroscopy. Because of the poor solubility of Al^3+^ complexes **1** and **3** in D_2_O, a highly soluble reference compound (**5b**) was used instead (Figure 6). ^13^C­{^1^H} NMR spectra of two samples were recorded: (a) spectrum of 10 mM solution of **5b** in D_2_O and (b) spectrum of identical solution with 1.0 equiv of AlCl_3_ added. Relative heights of the signals of the two nuclei (*A* and *B*) were significantly reduced. The height of signal *A* originating from the carbonyl group carbon nucleus was reduced by at least 80%. Moreover, the intensity of signal *B* originating from the nucleus in the proximity of the carbonyl group was reduced by 70%. The observed changes were ascribed to the accelerated chemical exchange involving the carbonyl group or the proximity of quadrupolar ^27^Al nucleus.^68,69^ Overall, this observation suggested that the carboxyl group was the most involved in the binding of group 13 cations by the synthesized receptors.

**Figure 6:**
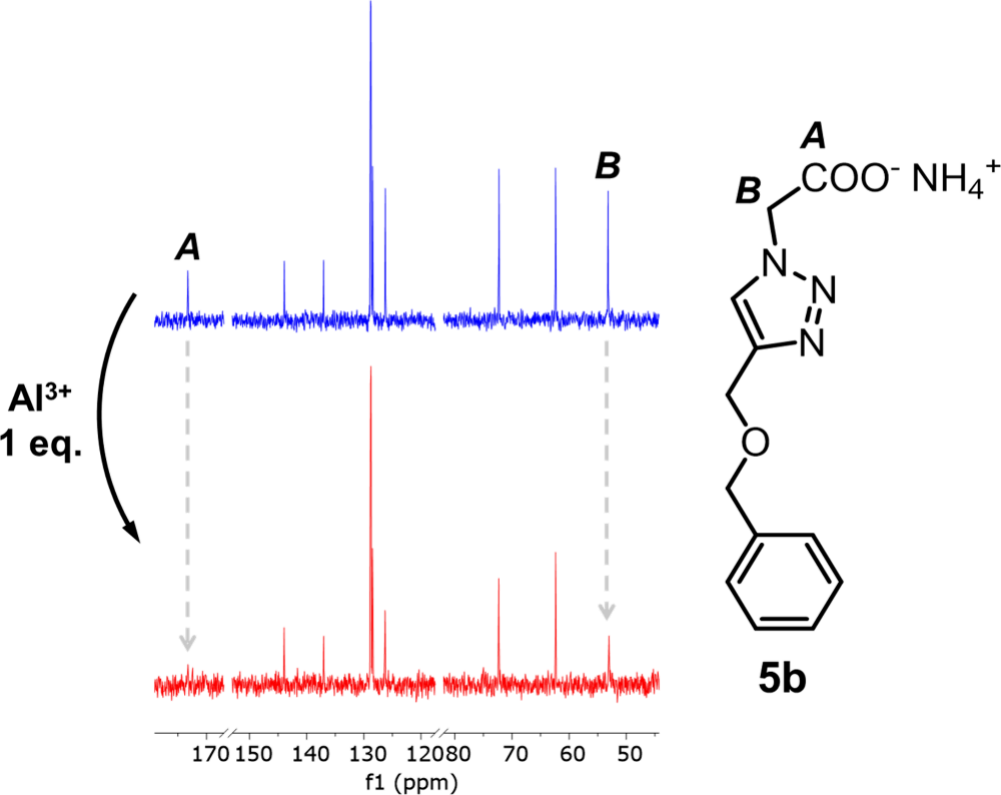
Insets of the ^13^C­{^1^H} NMR spectra of 10 mM solution of **5b** in D_2_O. Blue: without AlCl_3_ added; red: 1.0 equiv of AlCl_3_ added. Relative heights of signals *A* and *B* change on adding AlCl_3_.

A possible structure of complex of **1** with Al^3+^ cations was proposed based on literature data, theoretical predictions, and experimental observations (Figure 7). The complex was modeled using molecular dynamics and refined using 3-corrected Hartree–Fock (HF-3c) composite method (see the SI for computational details). In the proposed structure, **1** binds to two Al^3+^ cations. The complex is electroneutral. Thus, the proposed stoichiometry is in accord with fluorometric titration results. Each Al^3+^ cation is coordinated to three carboxylic groups and three molecules of hydration water. This proposed coordination is based on the NMR study involving analogue **5b**, and it was reported to be typical to Al^3+^ carboxylate complexes.^70,71^ In addition to the interactions involving Al^3+^, a network of hydrogen bonds and lipophilic interactions was predicted to be present in the complex. These interactions are thought to stabilize the complex.

**Figure 7:**
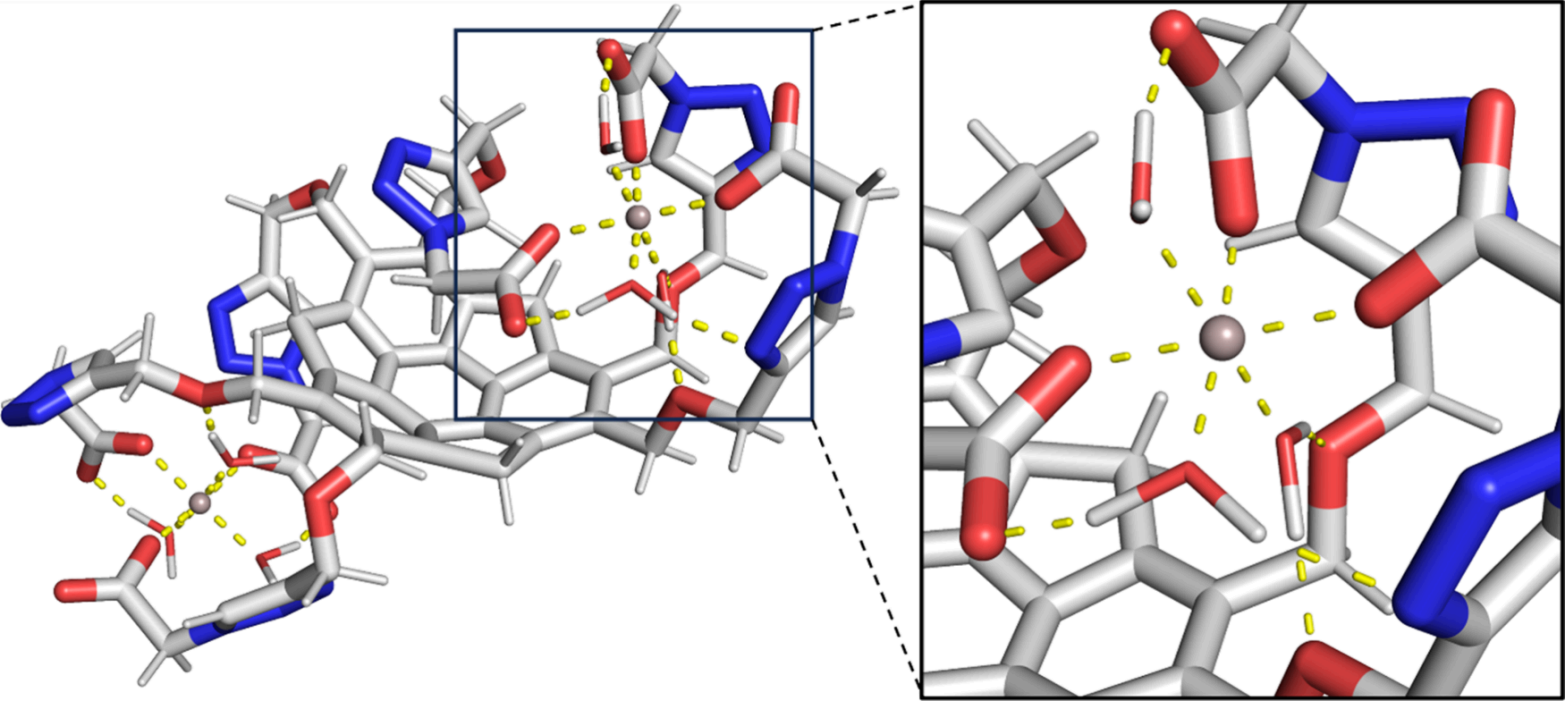
Structure of complex **1** with two Al^3+^ cations and hydration water. Coordination sphere of Al^3+^ is shown magnified on the right side of the figure. Beige – aluminum, gray – carbon, white – hydrogen, red – oxygen, blue – nitrogen, and yellow – chosen polar interactions. Each Al^3+^ cation is bound to three carboxylic groups of **1** and three hydration water molecules. Water creates a framework of hydrogen bonds with **1**, further stabilizing the complex. The structure was modeled using MD with QM optimization, and it is in agreement with the ^13^C NMR analysis of analogue **5b** and with titration data.

We envisioned that receptors **1** and **3** could be possibly applied to surface or groundwater monitoring for Al^3+^, Ga^3+^, or In^3+^ contamination. Al^3+^ cations are toxic and a common groundwater contaminant.^72–74^ Ga^3+^ and In^3+^ are substantially toxic^75^ and commonly found in electronics, such as LEDs^76,77^ or flexible electronic devices.^78–80^ Thus, to further verify the applicability of the obtained receptors, the receptor properties of **1** in real-life samples were examined. The real-life surface water samples were collected from six locations in Kansai region, Japan, in summer (for precise locations and experimental methodology, see the SI). Out of the six samples, three were collected from lakes and ponds. These three samples displayed intense absorbance above 250 nm and intense fluorescence (SI). Hence, they were presumed to be rich in organic matter. The other three samples were collected from mountainous streams and a mountain spring. They did not display significant absorbance or fluorescence and were presumed to be poor in organic matter. Al^3+^ content in each sample was measured using ICP-AES. Expected fluorescence of the receptor in the sample was then calculated using the modified Sten-Volmer equation (SI) based on the measured Al^3+^ content (Figure 8, light blue). Receptor **1** and the buffer were then added to each sample. Fluorescence of each sample was measured (Figure 8, dark blue). In samples 1–3 (rich in organic content), the observed fluorescence was significantly higher than expected. This could be due to masking of Al^3+^ cations by organic humic acids.^81^ In samples 4–6 (poor organic content), only small deviations were observed. Al^3+^ ions were then added to each sample. Observed and expected fluorescence after adding Al^3+^ ions was compared (Figure 8, dark red and light red). Again, in samples 1–3 (rich in organic content), the observed fluorescence was significantly higher than expected, while in samples 4–6 (poor in organic content), only small deviations were observed.

**Figure 8:**
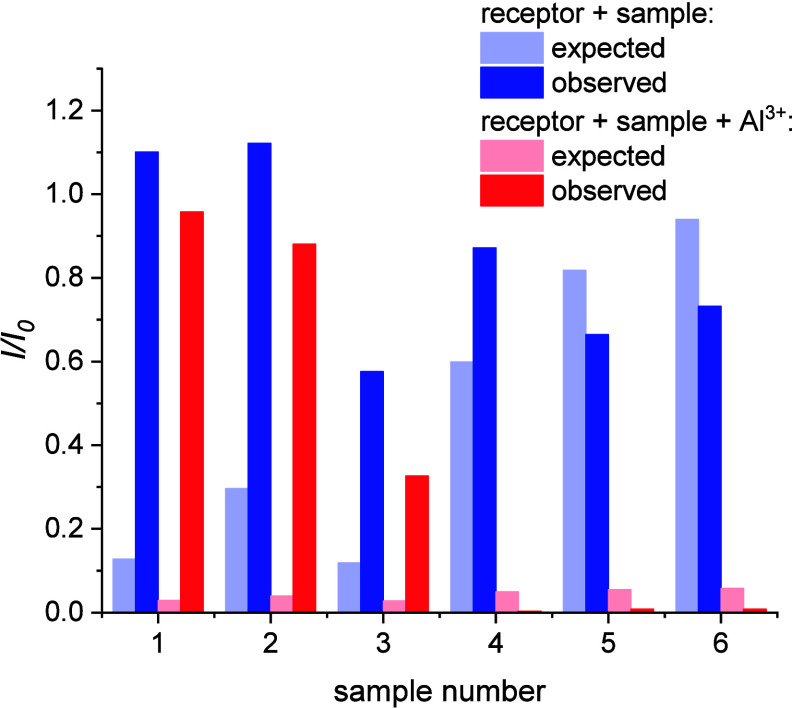
Fluorescence of **1** in real-life water samples 1–6, with and without 5 μM Al^3+^ added. Expected values (light blue, light red) were compared with observed values (dark blue, dark red). Predicted values were calculated from ICP/AES-determined Al^3+^ concentrations using the modified Stern–Volmer equation. Samples 4–6 were free of organic contamination and performed better. Receptor concentration: 0.1 μM; 1 mM of pH 5.0 AcOH/TRIS buffer added; Al^3+^ concentration: 5 μM (only in red measurements).

## Conclusions

In conclusion, we obtained four water-soluble group 13 cation receptors with sumanene or triphenylene cores. Synthesis of the receptors was achieved by attaching polar arm fragments to aromatic core fragments via the click chemistr*y* approach. Water solubilities of the synthesized receptors varied from 46 μM to 820 μM. We observed a strong correlation between the measured solubility values and MD-calculated free energy of dimerization values, suggesting that such calculations could be a useful component of solubility prediction models. Furthermore, we showed that some of the synthesized compounds were useful as optical receptors. Compounds **1** and **3** selectively detected Al^3+^, Ga^3+^, and In^3+^ in aqueous solutions with LOD values ranging from 90 to 370 nM. We identified the carboxylic group as the cation-binding site by ^13^C­{^1^H} NMR spectroscopy and modeled the complex using MD and QM composite methods. Performance of receptor **1** was further examined by attempting Al^3+^ detection in real-life surface water samples. We found that **1** could be used to detect Al^3+^ ions in water samples poor in organic content. Therefore, **1** was demonstrated to be useful in monitoring groundwater or surface water in mountainous regions. This work not only demonstrates the possibility of synthesizing sumanene- and triphenylene-based water-soluble cation receptors but also sheds new light on possible approaches to the design of water-soluble aromatic dendrimers.

## Methods

Chemical reagents and solvents were commercially purchased and purified according to the standard methods if necessary. Sumanene^11^ and azidoacetic acid^82^ were synthesized following the literature procedures. Structural assignments were made using additional information from gCOSY, gHSQC, and gHMBC experiments.

### Microwave Experiments

A Biotage initiator + reactor was used in microwave experiments. Reactions were conducted in sealed 2 mL vials. Reaction times and temperatures are specified in the relevant preparative procedures.

### NMR Experiments

The experiments were carried out using a Varian JEOL JNM-ECZS400 spectrometer (^1^H at 400 MHz, ^13^C­{^1^H} NMR at 101 MHz). Unless indicated otherwise, the spectra were recorded at 25 °C, and standard 5 mm NMR tubes were used. ^1^H and ^13^C chemical shifts (δ) were reported in parts per million (ppm) relative to the solvent signal, i.e., chloroform-d: δH (residual chloroform) 7.26 ppm, δC (residual chloroform) 77.16 ppm; DMSO-d_6_: δH (residual DMSO) 2.50 ppm, δC (residual DMSO) 40.45 ppm; D_2_O: δH (residual D_2_O) 4.79 ppm.

^1^H DOSY NMR (bpp_led_dosy_pfg JEOL pulse sequence) parameters are as follows: 16 scans, gradients 10–300 mT·m^–1^ in equal logarithmic increments, τ = 2 ms, diffusion time = 100 ms, Δ = 6.5 ms. In each case, the sample concentration was 10 mM, the temperature was 30 °C, and 3 mm NMR tubes were used to suppress diffusion. NMR spectra were analyzed with MestReNova v14.1software (Mestrelab Research S.L). The hydrodynamic radius from ^1^H DOSY NMR experiment was estimated using unmodified Stokes–Einstein equation (eq 1).^83,84^

Unmodified Stokes–Einstein equation: *r*_H,solv_ – hydrodynamic radius, *k*_B_ – Boltzmann constant, *T* – temperature of ^1^H DOSY NMR spectrum acquisition (303 K), and η – viscosity of the solvent (DMSO) at temperature *T* (0.001808 kg·m^–1^·s^–1^)­

$$r_{H,\mathrm{solv}}=\frac{k_{B}T}{6\pi \eta D}$$ 1

### Absorption Spectra

Measurements were performed with a Jasco V-670 spectrometer with the following parameters: UV/vis bandwidth – 2 nm, data interval – 1.0 nm, and response – fast. A quartz measurement cell was used.

### Emission Spectra

Measurements were performed with a Jasco FP-8550 spectrometer with the following parameters: excitation bandwidth – 5 nm, emission bandwidth – 5 nm, data interval – 0.5 nm, and response – 0.1 s. A quartz measurement cell was used.

### Single-Crystal Preparation

Monocrystal of **9** was obtained by evaporating a solution of 1 mg of 9 in 0.5 mL of diethyl ketone at RT.

### Single-Crystal Diffraction

The diffraction data were recorded on a XtaLAB Synergy with a Cu target (λ = 1.54184 Å) equipped with a Rigaku HyPix-6000HE as the detector at 123 K in house. The diffraction images were processed by using CrysAlisPro.^85^ The structures were solved by a direct method (SHELXT-2015, 2018/2)^86^ and refined by full-matrix least-squares calculations on F2 (SHELXL-2018/3)^87^ using the Olex2 ^88^ program package.

### Atomic Emission Spectroscopy

A Shimadzu ICPS-8100 emission spectrometer was used to perform ICP/AES. Samples **1–5** were prepared by dissolving 1–2 mg of each compound in 150 μL of aqua regia, heating in 90 °C for 5 min, diluting the sample to 10.00 mL with deionized water, and filtering it through a 0.5 μm syringe filter. Cu^2+^ content in **1–5** was then calculated based on the initial mass of the compound and the Cu^2+^ content in the ICP/AES sample. Real-life water samples were subjected to ICP/AES as-they-were after filtration through a 0.5 μm syringe filter.

### HRMS

ESI-HRMS and MALDI-HRMS spectra were measured on a JEOL JMS-T100LP spectrometer. EI-HRMS spectra were measured on a JEOL JMS-700 spectrometer.

### Melting Point

Melting point of **5** was determined on an Optimelt MPA100 automated melting point apparatus (Stanford Research Systems, Inc.) and expressed without correction.

### Molar Absorption Coefficient

Stock solutions of **1–4** (0.1 or 0.01 mM concentration) were used to prepare a series of solutions of a given compound in pure water in at least five different concentrations between 0 and 10 μM. Absorbance of the solutions at their respective absorption maxima was measured (**1**–290 nm, **2**–278 nm, **3**–275 nm, and **4**–269 nm). Molar absorption coefficient was determined by fitting the obtained data to the Lambert–Beer equation.

### Solubility

1.0 mg portion of the compound was placed in a small vial, 0.5 mL of deionized H_2_O was added, and the vial was closed. The vial and its contents were sonicated for 5 min at RT, vigorously shaken, and left for 1 h at RT. This was repeated six times. The vial was then left for next 12 h at RT. Supernatant was then collected and filtered through a 0.22 μm syringe filter. The solution obtained this way was considered saturated. It was diluted 10 times (**4**), 50 times (**1**), or 100 times (**2, 3**). Absorbance at the respective absorption maximum was measured (**1**–290 nm, **2**–278 nm, **3**–275 nm, and **4**–269 nm). Solubility (concentration of the saturated solution) was calculated based on the applied dilution, Lambert–Beer equation, and molar absorption coefficient obtained beforehand.

### Cation-Binding Experiments (Qualitative)

Cation-binding experiments between compounds **1–4** (chemosensors) and cations (analytes; Li^+^, Na^+^, Cs^+^, NH_4_^+^, Mg^2+^, Zn^2+^, Cu^2+^, Ni^2+^, Co^2+^, La^3+^, Eu^3+^, Al^3+^, Ga^3+^, In^3+^, Sn^4+^, Cr^3+^, and Sc^3+^) were performed employing emission spectra measurements. Cations were introduced in the form of their corresponding chloride salts. The experiments were performed as follows. In a 5 mL volumetric flask, stock solutions of **1–4** (10 μM) were diluted with an adequate volume of deionized H_2_O to reach a volume of approximately 3 mL. 0.5 mL of solution of AcOH/Tris buffer (10 mM) was added. An appropriate volume of stock solution of cation (0.1 mM) was added. H_2_O was added to reach the final volume of 5 mL. The final concentration of **1–4** in each sample equaled 0.10 μM. The final concentration of the cation in each sample equaled 5.0 μM. Excitation wavelength (λ_ex_) for the corresponding receptors was as follows: 289 nm (**1**), 278 nm (**2**), 271 nm (**3**), and 266 nm (**4**). Fluorescence intensity data were collected at the following emission wavelengths: 390 nm (**1**), 375 nm (**2**), 376 nm (**3**), and 383 nm (**4**).

### Cation-Binding Experiments (Quantitative)

Cation-binding experiments between compounds **1, 3**, and **4** (chemosensors) and the chosen cations (analytes; Al^3+^, Ga^3+^, and In^3+^) were performed employing the emission spectra measurements. Cations were introduced in the form of their corresponding chloride salts. The experiments were performed as follows. In a 5 mL volumetric flask, stock solution of **1**, **3**, or **4** (10 μM) was diluted with an adequate volume of deionized H_2_O to reach a volume of approximately 3 mL. 0.5 mL of a solution of AcOH/Tris buffer (10 mM) was added. An appropriate volume of stock solution of cation (0.01, 0.1, or 1 mM) was added. H_2_O was added to reach the final volume of 5 mL. Final concentration of **1–4** in each sample equaled 0.10 μM. Excitation wavelength (λ_ex_) for the corresponding receptors was as follows: 289 nm (**1**), 278 nm (**2**), 271 nm (**3**), and 266 nm (**4**). Fluorescence intensity data were collected at the following emission wavelengths: 390 nm (**1**), 375 nm (**2**), 376 nm (**3**), and 383 nm (**4**). For the chosen receptor-cation pairs, modified Stern–Volmer plots were used for the evaluation of detection parameters, namely, Stern–Volmer constants (*K*_SV_), average numbers of binders (*n*), and LOD values.^89^ This was done by fitting the binding data to the modified Stern–Volmer equation (eq 2).^65–67^ Fitting was done using the ″Simple Fit” functionality of Origin2024 software.

Modified Stern–Volmer equation was used to describe the quenching. *I*_0_ – fluorescence of the receptor before adding the cation, *I* – fluorescence after adding the cation, *K*_SV_ – Stern–Volmer constant, *n* – number of molecules bound to one receptor, and *c*_ion_ – ion concentration.

$$\log\left(\frac{I_{0}-I}{I}\right)=\log(K_{\mathrm{SV}})+n\cdot \log(c_{\mathrm{ion}})$$ 2

### Cation-Binding Experiments (Binding Site Determination)

Twenty mM stock solutions (20 mM) of 5b and AlCl_3_ in D_2_O were prepared by dissolving appropriate quantities of these compounds in D_2_O. Two NMR samples were then prepared. Sample 1 was prepared by mixing 250 μL of 5b stock solution and 250 μL of D_2_O. Sample 2 was prepared by mixing 250 μL of 5b stock solution and 250 μL of 5b AlCl_3_ stock solution. ^13^C­{^1^H} NMR spectra of both solutions were then measured.

### Cation-Binding Experiments (Real-Life Samples)

Samples were collected on August 15th, 2024 in the locations indicated below (Table 4). After collection, the samples were filtered through a 0.5 μm syringe filter and subjected to analyses.

**Table 4:** Locations Where Real-Life Samples were Collected

sample number	geographic coordinates	location name	location description
1	34°49′21.0″N 135°31′06.1″E	Zuion’ike Pond	urban retention pond
2	35°00′04.8″N 135°53′32.7″E	Biwa Lake	large lake
3	35°12′14.3″N 135°52′40.3″E	Kojorougaike Pond	eutrophic mountain pond
4	35°11′35.2″N 135°53′22.7″E	Kusushinotaki Waterfall	mountain waterfall
5	35°11′57.7″N 135°52′54.2″E		mountain spring
6	35°12′04.5″N 135°53′15.6″E	Nohanarene River	mountain stream near its source

Absorption and fluorescence spectra of each sample were measured as follows. To a 5 mL volumetric flask was added 500 μL of 10 mM AcOH/Tris pH 5.0 buffer. 100 μL of distilled water was also added. The flask was filled with the respective water sample to obtain a total volume of 5 mL. Absorption spectra of each sample were then measured (SI, Plot S4). Fluorescence spectra of each sample at a 289 nm excitation wavelength were also measured (SI, Plot S5).

Fluorescence of 1 in real-life water samples was measured as follows. To a 5 mL volumetric flask was added 500 μL of 10 mM AcOH/Tris pH 5.0 buffer. 50 μL of 10 μM stock solution of 1 was also added. 50 μL of distilled water was then added. The flask was filled with the respective water sample to obtain the total volume of 5 mL. Fluorescence intensity of the sample at 289 nm excitation wavelength and 390 nm emission wavelength was then measured. Fluorescence of the sample without 1 (SI, Plot S5) was subtracted from the obtained value.

Fluorescence of 1 in real-life water samples in the presence of 5 μM Al^3+^ was measured as follows. To a 5 mL volumetric flask was added 500 μL of 10 mM AcOH/Tris pH 5.0 buffer. 50 μL of 10 μM stock solution of 1 was also added. 50 μL of 0.5 mM AlCl_3_ stock solution in water was then added. The flask was filled with the respective water sample to obtain the total volume of 5 mL. Fluorescence intensity of the sample at 289 nm excitation wavelength and 390 nm emission wavelength was then measured. Fluorescence of the sample without 1 (SI, Plot S5) was subtracted from the obtained value.

### Molecular Dynamics – General Methodology

GROMACS software version 2023.4 was used in the simulations.^90,91^ Starting geometries of molecules were constructed using Avogadro software, version 1.2.0.^92^ Sobtop tool^93^ was used to parametrize compounds **1–4** (GAFF force field,^94^ EEM charges^95^). SPC/E water model was used.^96^ Short-range electrostatic interaction cutoff was applied at 1.0 nm. Particle-mesh Ewald method was applied for long-range electrostatic interactions.^97,98^ Short-range dispersion interaction cutoff was applied at 1.0 nm, and long-range dispersion corrections for energy and pressure were applied. The temperature was set to 298 K. A Bussi-Donadio-Parinello (v-rescale) thermostat with 0.1 ps time constant was used.^99^ A Parrinello–Rahman barostat was used with isotropic coupling, 2.0 ps time constant, reference pressure set to 1.0 bar, and compressibility set to 4.5 × 10^–5^ bar^–1^.^100^ Periodic boundary conditions were used in all directions. Simulation step was set to 2 fs, and leapfrog integrator was used in the simulations.

### Free Energy of Dimerization Calculations

For sumanene derivatives (**1** and **2**), geometries of three types of dimers were independently constructed (a: convex-to-concave, b: convex-to-convex, and c: concave-to-concave). For triphenylene derivatives (**3** and **4**) geometry of one type of dimer was constructed. In each case, a dimer was placed in a rectangular box (5 nm × 5 nm × 7 nm). Solvent was added. Energy of the system was minimized. Reaction coordinate was defined as the distance between molecular cores (only carbon atoms) of monomers in the *Z* direction only. Additionally, the distance between the monomers was constrained in *X* and *Y* directions by an umbrella potential (500 kJ·mol^–1^·nm^–2^) in each simulation. Initial pulling was performed (0.6 ns, 0.0 → 3.0 nm, 500 kJ·mol^–1^·nm^–2^). Simulation frames were extracted from the initial trajectory (one frame every 0.1 nm, from 0.0 to 3.0 nm). Independent simulations were performed for each of these frames (umbrella potential constant: 2000 kJ·mol^–1^·nm^–2^, 11 ns, first 1 ns treated as equilibration time and discarded). Free-energy landscape was calculated from the sampling data using WHAM method.^101,102^ Error was estimated by using bootstrapping. Dimerization energy was determined as the value of the lowest minimum on the free energy of the dimerization profile. For sumanene derivatives, the lowest value obtained for all three dimers was used. Calculated free-energy profiles are provided in the SI (Section S7).

### Cation Structure Modeling

Two Al^3+^ cations and one anion of **1** (fully ionized, hexaanion) was placed in a 5 nm × 5 nm × 5 nm rectangular box. The following nonbonded parameters were used for Al^3+^ ions: charge: 3.0, mass: 26.98, sigma: 1.94126 × 10^–1^, epsilon: 9.77218 × 10^–3^.^103^ 32 independent MD simulations of the system were performed. The structures resulting from the simulations were visually inspected. They could be grouped in three groups: a) both Al^3+^ cations on the same (convex) side of the sumanene bowl, b) both Al^3+^ cations on the same (concave) side of the sumanene bowl, and c) Al^3+^ cations on the different sides of the sumanene bowl. For each of these groups, a representative structure was chosen. Water molecules were deleted so that only three water molecules closest to each Al^3+^ cation remained. Each of the three structures was then optimized using three-corrected Hartree–Fock method (HF-3c)^104^ with the CPCM water model.^105^ Orca 6.0.0 software was used.^106^ The energies of the complexes were compared. Lowest-energy structure was assumed to be the prevalent complex structure.

### Synthesis of Dendrimers

#### 2,2’,2’’,2’’’,2’’’’,2’’’’’-(4,4’,4’’,4’’’,4’’’’,4’’’’’-((((4,7-Dihydro-1H-Tricyclopenta­[def,jkl,pqr]­triphenylene-2,3,5,6,8,9-hexayl)­hexakis­(methylene))­hexakis­(oxy))­hexakis­(methylene))­hexakis­(1H-1,2,3-triazole-4,1-diyl))­hexaacetic acid, **1**

Alkyne **6** (8.4 mg, 1.0 equiv), copper­(I) thiophene-2-carboxylate (1.4 mg, 0.6 equiv), DMSO (1.0 mL), azidoacetic acid (11.2 μL, 12 equiv), and triethylamine (22.6 μL, 13 equiv) were sealed in a microwave vial under nitrogen flow. The mixture was heated at 80 °C for 20 min using microwave radiation. When the mixture cooled, 5 mL of 5% Na_2_CO_3_ (aq) was added. The mixture was washed with 5 × 10 mL of AcOEt. The pH of water phase was then adjusted to pH = 2 using concentrated HCl (aq). After 10 min, the precipitate was separated by membrane filtration and washed with 3 × 1 mL of 1% HCl (aq) and with 1 mL of deionized water. The product was then dried in vacuo to afford 12.7 mg (80%) of a green powder contaminated by Cu^2+^. The green powder was dissolved in 1 mL of 5% Na_2_CO_3_ (aq). A solution of Na_2_S·9H_2_O (23.9 mg, 10 equiv) in 0.5 mL of deionized water was added, upon which a black precipitate appeared. The mixture was left for 30 min without stirring. It was then filtered through a syringe filter (pore diameter: 0.22 μm). The pH was adjusted to pH = 2 using concentrated HCl (aq). After 10 min, the precipitate was separated by membrane filtration and washed with 3 × 1 mL of 1% HCl (aq) and 5 × 1 mL of deionized water. The product was then dried in vacuo (60 Pa) to afford **1** (9.2 mg, 61%) of as a yellow powder (<0.5 mol % Cu^2+^, measured by ICP-AES).

^1^H NMR (400 MHz, DMSO*-d*_6_) δ 8.09 (s, 6H), 5.24 (s, 12H), 4.51–4.73 (m, 27H), 3.59 (d, ^3^ *J*_HH_ = 20.2 Hz, 3H).

^13^C­{^1^H} NMR (101 MHz, DMSO*-d*_6_) δ: 168.6, 149.3, 147.1, 144.0, 133.0, 125.4, 67.1, 62.9, 50.4.

^1^H DOSY NMR (400 MHz, DMSO*-d*_6_) *D* 9.82 × 10^–11^ m^2^·s^–1^.

MALDI-HRMS *m*/*z* calcd. for C_57_H_54_N_18_O_18_ [M-H]^−^ 1277.3785; found 1277.3791.

#### 2,2’,2’’,2’’’,2’’’’,2’’’’’-(4,4’,4’’,4’’’,4’’’’,4’’’’’-((4,7-Dihydro-1H-tricyclopenta­[def,jkl,pqr]­triphenylene-1,1,4,4,7,7-hexayl)­hexakis­(methylene))­hexakis­(1H-1,2,3-triazole-4,1-diyl))­hexaacetic acid, **2**

**2** was prepared and isolated analogously to **1**. 9.2 mg of alkyne **8** was used. The product (8.2 mg, 40%) was obtained as an off-white powder (<0.5 mol % Cu^2+^, measured by ICP-AES).

^1^H NMR (400 MHz, DMSO*-d*_6_) δ: 7.98 (s, 3H), 6.69 (s, 6H), 6.65 (s, 3H), 5.26 (s, 6H), 4.93 (s, 6H), 3.80 (s, 6H), 2.78 (s, 6H).

^13^C­{^1^H} NMR (101 MHz, DMSO-*d*_6_) δ: 168.8, 168.5, 154.2, 145.2, 143.4, 142.9, 125.3, 124.3, 123.6, 62.4, 50.4, 50.3, 34.1, 31.3.

^1^H DOSY NMR (400 MHz, DMSO*-d*_6_) *D* 2.04 × 10^–10^ m^2^·s^–1^.

MALDI-HRMS *m*/*z* calcd. for C_51_H_42_N_18_O_12_ [M-H]^−^ 1097.3157; found 1097.3127.

#### 2,2’,2’’,2’’’,2’’’’,2’’’’’-(4,4’,4’’,4’’’,4’’’’,4’’’’’-(((Triphenylene-2,3,6,7,10,11-hexaylhexakis­(methylene))­hexakis­(oxy))­hexakis­(methylene))­hexakis­(1H-1,2,3-triazole-4,1-diyl))­hexaacetic acid, **3**

**3** was prepared and isolated analogously to **1**. 4.0 mg portion of alkyne **11** was used. The product (2.1 mg, 26%) was obtained as a beige powder (<0.5 mol % Cu^2+^, measured by ICP-AES).

^1^H NMR (400 MHz, DMSO*-d*_6_) δ 8.81 (s, 6H), 8.18 (s, 6H), 5.29 (s, 12H), 4.87 (s, 12H), 4.71 (s, 12H).

^13^C­{^1^H} NMR (101 MHz, DMSO-*d*_6_) δ: 169.2, 144.4, 136.6, 128.8, 126.1, 124.3, 69.9, 63.7, 51.0.

^1^H DOSY NMR (400 MHz, DMSO*-d*_6_) *D* 9.82 × 10^–11^ m^2^·s^–1^.

MALDI-HRMS *m*/*z* calcd. for C_54_H_54_N_18_O_18_ [M]^+^ 1242.3869; found 1242.3870.

#### 2,2’,2’’,2’’’,2’’’’,2’’’’’-(4,4’,4’’,4’’’,4’’’’,4’’’’’-((Triphenylene-2,3,6,7,10,11-hexaylhexakis­(oxy))­hexakis­(methylene))­hexakis­(1H-1,2,3-triazole-4,1-diyl))­hexaacetic acid, **4**

**4** was prepared and isolated analogously to **1**. 20.7 mg of alkyne **11** was used. The product (42.6 mg, 98%) was obtained as a gray powder (<0.5 mol % Cu^2+^, measured by ICP-AES).

^1^H NMR (400 MHz, DMSO-*d*_6_) δ 8.35 (s, 6H), 8.33 (s, 6H), 5.51 (s, 12H), 5.29 (s, 12H).

^13^C­{^1^H} NMR (101 MHz, DMSO-*d*_6_) δ: 168.6, 147.6, 142.7, 126.4, 123.0, 107.2, 61.7, 50.7.

^1^H DOSY NMR (400 MHz, DMSO*-d*_6_) *D* 8.81 × 10^–11^ m^2^·s^–1^.

MALDI-HRMS *m*/*z* calcd. for C_48_H_42_N_18_O_18_ [M-H]^+^ 1157.2841; found 1157.2838.

#### 2,3,5,6,8,9-Hexakis­(bromomethyl)-4,7-dihydro-1H-tricyclopenta­[def,jkl,pqr]­triphenylene, **6**

**6** was prepared from pristine sumanene following the procedure reported by Sakurai et al.^37^ **5** was obtained as a yellow powder (309.0 mg, 99%).

^1^H NMR (400 MHz, CDCl_3_) δ 4.79 (ABq-d, *J*_AB_ = 14.1 Hz, ^2^ *J*_HH_ = 11.6 Hz, 12H), 4.70 (d, ^2^ *J*_HH_ = 19.4 Hz, 3H), 3.80 (d, ^2^ *J*_HH_ = 19.4 Hz, 3H).

#### 2,3,5,6,8,9-Hexakis­((prop-2-yn-1-yloxy)­methyl)-4,7-dihydro-1H-tricyclopenta­[def,jkl,pqr]­triphenylene, **7**

A mixture of **5** (100.0 mg, 1.0 equiv), *n*-Bu_2_SnO (200 mg, 6.6 equiv), and propargyl alcohol (4.0 mL) was heated in 80 °C for 3 h in a N_2_ atmosphere. The mixture was then cooled down, quenched with 30 mL H_2_O, and extracted with 3 × 30 mL of DCM. It was then desiccated with Na_2_SO_4_ and filtered. Solvent and volatile fractions were removed in vacuo. The mixture was purified by PTLC (ethyl acetate: hexane 1:2) to afford **6** (65.7 mg, 82%) as a yellow solid.

^1^H NMR (400 MHz, CDCl_3_) δ 4.69–4.86 (m, 15H), 4.20 (ABq-d, *J*_AB_ = 33.5 Hz, ^2^ *J*_HH_ = 16.0 Hz, ^4^ *J*_HH_ = 2.3 Hz, 12H) 3.74 (d, ^2^ *J*_HH_ = 19.6 Hz, 3H), 2.50 (t, ^4^ *J*_HH_ = 2.3 Hz, 6H).

^13^C­{^1^H} NMR (101 MHz, CDCl_3_) δ: 150.2, 148.4, 132.2, 80.1, 74.9, 67.2, 57.3, 41.1.

EI-HRMS *m*/*z* calcd. for C_45_H_36_O_6_ [M]^+^ 672.2512; found 672.2528.

#### 1,1,4,4,7,7-Hexa­(prop-2-yn-1-yl)-4,7-dihydro-1H-tricyclopenta­[def,jkl,pqr]­triphenylene, **9**

**9** was prepared using a procedure similar to the one reported by Hirao et al.^38^ Pristine sumanene (**7**, 30.0 mg, 1 equiv), tetrabutylammonium bromide (43.8 mg, 1.2 equiv), and propargyl bromide (102 μL, 12 equiv) were suspended in THF (1.0 mL). Four ml of 30% NaOH (*aq*) was added. The mixture was stirred for 46 h in RT in a N_2_ atmosphere. The mixture was quenched with 20 mL of H_2_O, extracted with 3 × 20 mL of DCM, desiccated with Na_2_SO_4_, and filtered. Solvent and volatile fractions were removed in vacuo. The mixture was purified by PTLC (CHCl_3_: hexane 3:2) to afford **9** (49.0 mg, 88%) as a yellow solid.

^1^H NMR (400 MHz, CDCl_3_) δ 7.32 (s, 6H), 3.45 (d, ^4^ *J*_HH_ = 2.5 Hz, 6H), 2.50 (d, ^4^ *J*_HH_ = 2.5 Hz, 6H), 2.22 (t, ^4^ *J*_HH_ = 2.5 Hz, 3H), 2.07 (t, ^4^ *J*_HH_ = 2.5 Hz, 3H).

^13^C­{^1^H} NMR (101 MHz, CDCl_3_) δ: 154.9, 146.0, 122.9, 81.20, 81.1, 71.6, 71.3, 59.5, 28.8, 24.6.

EI-HRMS *m*/*z* calcd. for C_39_H_24_ [M]^+^ 492.1878; found 492.1875.

#### Hexamethyl 1,4,5,8,9,12-hexahydrotriphenylene-2,3,6,7,10,11-hexacarboxylate

Prepared from hexakis­(bromomethyl)­benzene (2.00 g) following the procedure reported by Fukushima et al.,^39^ hexamethyl 1,4,5,8,9,12-hexahydrotriphenylene-2,3,6,7,10,11-hexacarboxylate (123.9 mg, 6.8%) was obtained as a white solid and used in the next step without further purification.

^1^H NMR (400 MHz, CDCl_3_) δ 3.85 (s, 18H), 3.55 (s, 12H).

#### Hexamethyl triphenylene-2,3,6,7,10,11-hexacarboxylate, **10**

Prepared from hexamethyl 1,4,5,8,9,12-hexahydrotriphenylene-2,3,6,7,10,11-hexacarboxylate following the procedure similar to the one reported by Fukushima et al.,^39^ hexamethyl 1,4,5,8,9,12-hexahydrotriphenylene-2,3,6,7,10,11-hexacarboxylate (123.9 mg, 1.0 equiv) and MnO_2_ (495.6 mg, 27 equiv) were suspended in toluene (31 mL). The mixture was stirred at 65 °C for 16 h. It was then cooled and filtered through a SiO_2_ plug. The plug was washed with 5% MeOH in CHCl_3_. Volatile fractions were removed from the filtrate in vacuo, yielding a solid residue. The residue was purified by PTLC (MeOH: CHCl_3_ 2:98) to afford **10** (80.2 mg, 65%) as a white solid.

^1^H NMR (400 MHz, CDCl_3_) δ 9.02 (s, 6H), 4.05 (s, 18H).

#### Triphenylene-2,3,6,7,10,11-hexaylhexamethanol, **11**

**9** (20.2 mg, 1.0 equiv) was put in a flame-dried microwave vial. The vial was then evacuated and filled with N_2_. Dry THF (1 mL) was added, and the vial was cooled to 0 °C. A solution of LiAlH_4_ (16.0 mg, 12 equiv) in dry THF (2 mL) was then slowly added. The mixture was heated in 80 °C for 2 h using microwave radiation. The mixture was quenched with 5 mL of 10% HCl (*aq*), upon which a white precipitate formed. The precipitate was separated using a membrane filter and washed with 10 mL of 10% NaOH (aq) and with 20 mL of deionized H_2_O. It was then dried in vacuo (60 Pa) to afford **11** (8.0 mg, 56%) as a white solid.

^1^H NMR (400 MHz, DMSO-*d*_6_) δ 8.75 (s, 6H), 5.37 (t, ^3^ *J*_HH_ = 5.4 Hz, 6H), 4.81 (d, ^3^ *J*_HH_ = 5.4 Hz, 12H).

^13^C­{^1^H} NMR (101 MHz, DMSO-*d*_6_) δ: 138.7, 127.7, 121.1, 60.6.

MALDI-HRMS *m*/*z* calcd. for C_24_H_24_O_6_ [M + Na]^+^ 431.1465; found 431.1465.

#### 2,3,6,7,10,11-Hexakis­((prop-2-yn-1-yloxy)­methyl)­triphenylene, **12**

**12** was prepared using a procedure similar to the one reported by Sakamoto et al.^40^ Compound **11** (8.0 mg, 1 equiv), KOH (13.2 mg, 12 equiv), and propargyl bromide (26.5 μL, 18 equiv) were suspended in DMSO (0.5 mL). The mixture was stirred for 2 h in RT in a N_2_ atmosphere, after which TLC analysis revealed that the reaction was not complete. Propargyl bromide (26.5 μL, 18 equiv) was added, and the reaction was continued for 1 h in RT in a N_2_ atmosphere. The reaction was then quenched with 10 mL of H_2_O. The precipitate was separated by membrane filtration, washed on the filter using 20 mL of deionized H_2_O, and dried in vacuo. It was then purified by PTLC (MeOH: CHCl_3_ 2:98) to yield **12** (4.0 mg, 32%) as a yellow solid.

^1^H NMR (400 MHz, CDCl_3_) δ 8.68 (s, 6H), 4.96 (s, 12H), 4.32 (d, ^4^ *J*_HH_ = 2.3 Hz, 12H), 2.55 (t, ^4^ *J*_HH_ = 2.3 Hz, 6H).

^13^C­{^1^H} NMR (101 MHz, CDCl_3_) δ: 135.1, 129.4, 124.6, 79.9, 75.1, 69.7, 57.7.

ESI-HRMS *m*/*z* calcd. for C_42_H_36_O_6_ [M + Na]^+^ 659.2404; found 659.2403.

#### 2,3,6,7,10,11-Hexamethoxytriphenylene

Prepared from veratrole following the procedure reported by Guillon et al.,^107^ 2,3,6,7,10,11-hexamethoxytriphenylene was obtained as a white solid (1096 mg, 81%).

^1^H NMR (400 MHz, CDCl_3_) δ: 7.83 (s, 6H), 4.14 (s, 18H).

#### 2,3,6,7,10,11-Hexahydroxytriphenylene, **13**

Prepared from 2,3,6,7,10,11-hexamethoxytriphenylene following the procedure reported by Zhang et al.,^108^ **13** was obtained as a purple solid (407.3 mg, 99%).

^1^H NMR (400 MHz, DMSO-*d*_6_) δ 9.28 (s, 6H), 7.60 (s, 6H).

#### 2,3,6,7,10,11-Hexakis­(prop-2-yn-1-yloxy)­triphenylene, **14**

A Schlenk vial was flame-dried, evacuated, and filled with N_2_. Compound **13** (100.0 mg, 1 equiv), K_2_CO_3_ (383.6 mg, 9 equiv), and dry DMF (5 mL) were introduced into the flask. Propargyl bromide (210.3 μL, 9 equiv) was then added. The mixture was stirred for 8 days in RT in a N_2_ atmosphere. H_2_O (30 mL), AcOEt (15 mL), and hexane (15 mL) were then added to the mixture, upon which a white precipitate of **14** appeared. The precipitate was separated on a membrane filter and washed with H_2_O (30 mL), AcOEt (10 mL), and hexane (30 mL). It was then dried in vacuo to afford **14** (140.0 mg, 82%) as an off-white solid.

^1^H NMR (400 MHz, DMSO-*d*_6_) δ 8.15 (s, 6H), 5.10 (d, ^4^ *J*_HH_ = 2.3 Hz, 12H), 3.61 (t, ^4^ *J*_HH_ = 2.3 Hz, 6H).

^13^C­{^1^H} NMR (101 MHz, DMSO-*d*_6_) δ: 146.9, 123.23, 107.9, 79.1, 78.7, 56.5.

EI-HRMS *m*/*z* calcd. for C_36_H_24_O_6_ [M]^+^ 552.1573; found 552.1579.

#### Prop-2-ynoxymethylbenzene, **16**

**16** was prepared using a procedure similar to the one reported by Boger et al.^109^ Benzyl alcohol (104 μL, 1.0 equiv), KOH (100 mg, 1.8 equiv), and propargyl bromide (91 μL, 1.2 equiv) were dissolved in DMSO (1.0 mL). The mixture was stirred for 16 h in RT in a N_2_ atmosphere. It was then quenched with 20 mL of H_2_O and extracted with 3 × 20 mL of diethyl ether. The extract was washed with brine three times, desiccated over Na_2_SO_4_, and carefully evaporated in vacuo. It was then purified by PTLC (hexane: AcOEt 1:1) to afford prop-2-ynoxymethylbenzene (118.4 mg, 81%) as a yellow oil. Prop-2-ynoxymethylbenzene was used in the next step without further purification.

^1^H NMR (400 MHz, CDCl_3_) δ 8.15 (s, 6H), 5.10 (d, ^4^ *J*_HH_ = 2.3 Hz, 12H), 3.61 (t, ^4^ *J*_HH_ = 2.3 Hz, 2H), 2.47 (t, ^4^ *J*_HH_ = 2.3 Hz, 1H).

#### 2-(4-((Benzyloxy)­methyl)-1H-1,2,3-triazol-1-yl)­acetic acid, **5**

Prop-2-ynoxymethylbenzene (61.0 mg, 1.0 equiv), CuTC (8.0 mg, 0.1 equiv), DMSO (2.0 mL), azidoacetic acid (62.5 μL, 2.0 equiv), and triethylamine (128 μL, 2.2 equiv) were sealed in a microwave vial under a nitrogen flow. The mixture was heated in 80 °C for 20 min using microwave radiation. When the mixture cooled down, 10 mL of 5% Na_2_CO_3_ (aq) was added. The mixture was washed with 5 × 10 mL of AcOEt. The pH of the water phase was then adjusted to pH = 2 using concentrated HCl (*aq*). No precipitate appeared. The mixture was extracted with 3 × 10 mL of CHCl_3_. The extract was desiccated over Na_2_SO_4_. Volatile fractions were removed in vacuo. The obtained residue was recrystallized from 4.0 mL of deionized water with hot filtration, washed with cold deionized H_2_O, and dried to afford **5** (48.3 mg, 47%) as colorless prismatic crystals (mp = 128.5–129.5 °C; <0.5 mol % Cu^2+^, measured by ICP-AES).

^1^H NMR (400 MHz, DMSO-*d*_6_) δ: 8.11 (s, 1H), 7.25–7.40 (m, 5H), 5.26 (s, 2H), 4.58 (s, 2H), 4.53 (s, 2H).

^13^C­{^1^H} NMR (101 MHz, DMSO-*d*_6_) δ: 168.7, 143.9, 138.2, 128.3, 127.7, 127.6, 125.4, 71.3, 62.8, 50.5.

^1^H DOSY NMR (400 MHz, DMSO*-d*_6_) *D* 2.48 × 10^–10^ m^2^·s^–1^.

ESI-HRMS *m*/*z* calcd. for C_12_H_13_N_3_O_3_Na [M + Na]^+^ 270.0849; found 270.0854.

#### Ammonium 2-(4-((benzyloxy)­methyl)-1H-1,2,3-triazol-1-yl)­acetate, **5b**

2-(4-((Benzyloxy)­methyl)-1H-1,2,3-triazol-1-yl)­acetic acid **5** was dissolved in MeOH (1 mL). 14.8 M NH_3_ (14.8 M, aq; 10.0 μL, 4.0 equiv) was added. The mixture was stirred in RT for 5 min. Volatile fractions were removed in vacuo. **5b** was obtained as a white solid (10.5 mg, 100%); pH of water solution: 7.

^1^H NMR (400 MHz, D_2_O, PreSAT water suppression) δ 8.02 (s, 1H), 7.43–7.52 (m, 5H), 5.10 (s, 2H), 4.82 (s, 2H), 4.70 (s, 2H).

^13^C­{^1^H} NMR (101 MHz, D_2_O) δ: 173.3, 143.9, 137.0, 128.8, 128.8, 128.5, 126.2, 72.8, 62.4, 53.2.

## Supplementary Material



## Data Availability

The data underlying this study are available in the published article and its Supporting Information.

## Notes

Materials and methods, experimental section, compound characterization data, details and additional data on cation-binding experiments including data on selectivity of **2** and **4**, MD and QM computational details, single-crystal structure of **9**, and single-crystal structure analysis report (PDF)
