# Supplementary material for: Structure-Dependent Water Solubility and Receptor Properties of C3‑Symmetric Dendrimers Bearing Sumanene or Triphenylene Cores
Source: ACS Org Inorg Au. 2025 Jul 22;5(5):347–59. doi: 10.1021/acsorginorgau.5c00048 (PMC12492046; doi:10.1021/acsorginorgau.5c00048)
Supplement: Supplementary file 1 [file gg5c00048_si_001.pdf]

Supporting Information (SI) for:

Structure-dependent water solubility and receptor properties of C_3-symmetric dendrimers bearing sumanene or triphenylene cores

Stanisław Kulczyk[a,b], Yumi Yakiyama[b,c], Mariola Koszytkowska-Stawińska[a], Hidehiro Sakurai[b,c]*, Artur Kasprzak[a]*

[a] Faculty of Chemistry, Warsaw University of Technology, Noakowskiego Str. 3, 00-664 Warsaw, Poland

[b] Division of Applied Chemistry, Graduate School of Engineering, The University of Osaka, 2-1 Yamadaoka, Suita, 565-0871 Osaka, Japan

[c] Innovative Catalysis Science Division, Institute for Open and Transdisciplinary Research Initiatives (ICS-OTRI), The University of Osaka, Suita, Osaka 565-0871, Japan

* corresponding authors e-mails: hsakurai@chem.eng.osaka-u.ac.jp (H.S), e-mail: artur.kasprzak@pw.edu.pl (A.K.)

Table of contents

S1. Methods 3

S2. Synthesis 8

S3. Spectral characterization data for compounds 1-14 17

S4. Thermal ellipsoid plot of 9 38

S5. Determination of molar absorption coefficient 40

S5. Cation binding experiments 41

S6. Real-life water sample characterization 43

S7. Free energy of dimerization profiles 45

S8. Literature survey 49

S9. Supporting references 51

S1. Methods

Chemical reagents and solvents were commercially purchased and purified according to the standard methods, if necessary. Sumanene[1] and azidoacetic acid[2] were synthesized following the literature procedures. Structural assignments were made with additional information from gCOSY, gHSQC, and gHMBC experiments.

Microwave experiments

Biotage Initiator+ reactor was used in microwave experiments. Reactions were conducted in sealed 2 ml vials. Reaction times and temperatures were specified in relevant preparative procedures.

NMR experiments

The experiments were carried out using a Varian JEOL JNM-ECZS400 spectrometer (^{1}H at 400 MHz, $^{13}C\{^{1}H\}$ NMR at 101 MHz). Unless indicated otherwise, spectra were recorded at 25 °C and standard 5 mm NMR tubes were used. ^{1}H and ^{13}C chemical shifts (δ) were reported in parts per million (ppm) relative to the solvent signal, *i.e.*, Chloroform-d: δH (residual chloroform) 7.26 ppm, δC (residual chloroform) 77.16 ppm; DMSO-d_6: δH (residual DMSO) 2.50 ppm, δC (residual DMSO) 40.45 ppm; D_2O: δH (residual D_2O) 4.79 ppm.

^{1}H DOSY NMR (bpp_led_dosy_pfg JEOL pulse sequence) parameters were as follows: 16 scans, gradients 10 - 300 mT $\cdot$ m^{-1} in equal logarithmic increments, τ = 2 ms, diffusion time = 100 ms, Δ = 6.5 ms. In each case, sample concentration was 10 mM, temperature was 30 °C and 3 mm NMR tubes were used to suppress diffusion. NMR spectra were analyzed with the MestReNova v14.1software (Mestrelab Research S.L). The hydrodynamic radius from ^{1}H DOSY NMR experiment was estimated using unmodified Stokes-Einstein equation:[3,4]

$$r_{H,solv} = \frac{k_B T}{6\pi\eta D}$$

Where: $r_{H,solv}$ – hydrodynamic radius, k_B – Boltzmann constant, T – temperature of ^{1}H DOSY NMR spectrum acquisition (303 K), η – viscosity of the solvent (DMSO) at temperature T (0.001808 kg $\cdot$ m^{-1} $\cdot$ s^{-1}).

Absorption spectra

Measurements were performed with Jasco V-670 spectrometer, with the following parameters: UV/Vis bandwidth – 2 nm, data interval – 1.0 nm, response – fast. A quartz measurement cell was used.

Emission spectra

Measurements were performed with Jasco FP-8550 spectrometer, with the following parameters: excitation bandwidth – 5 nm, emission bandwidth – 5 nm, data interval – 0.5 nm, response – 0.1 s. A quartz measurement cell was used.

Single crystal preparation

Monocrystal of **9** was obtained by evaporating a solution of 1 mg of **9** in 0.5 mL of diethyl ketone in room temperature.

Single crystal diffraction

The diffraction data were recorded on a XtaLAB Synergy with a Cu-target (λ = 1.54184 Å) equipped with a Rigaku HyPix-6000HE as the detector at 123 K in house. The diffraction images were processed by using CrysAlisPro.[5] The structures were solved by a direct methods (SHELXT-2015, 2018/2),[6] and refined by full-matrix least squares calculations on F2 (SHELXL-2018/3)[7] using the Olex2[8] program package.

Atomic emission spectroscopy

Shimadzu ICPS-8100 emission spectrometer was used to perform induced coupling plasma-atomic emission spectroscopy (ICP/AES). Samples of **1-5** were prepared by dissolving 1-2 mg of each compound in 150 µL of aqua regia, heating in 90 °C for 5 minutes, diluting the sample to 10.00 mL with deionized water and filtering it through a 0.5 µm syringe filter. Cu^{2+} content in **1-5** was then calculated based on initial mass of the compound and Cu^{2+} content in ICP/AES sample. Real-life water samples were subjected to ICP/AES as-they-were, after filtration through a 0.5 µm syringe filter.

High resolution mass spectroscopy (HRMS)

ESI-HRMS and MALDI-HRMS spectra were measured on JEOL JMS-T100LP spectrometer. EI-HRMS spectra were measured on JEOL JMS-700 spectrometer.

Melting point

Melting point of **5** was determined on an Optimelt MPA100 automated melting point apparatus (Stanford Research Systems, Inc.), and expressed without correction.

Molar absorption coefficient

Stock solutions of **1-4** (0.1 mM or 0.01mM concentration) were used to prepare a series of solutions of a given compound, in pure water, in at least five different concentrations between 0 µM and 10 µM. Absorbance of the solutions at their respective absorption maxima was measured (**1** – 290 nm, **2** – 278 nm, **3** – 275 nm, **4** – 269 nm). Molar absorption coefficient was determined by fitting the obtained data to Lambert-Beer equation.

Solubility

1.0 mg of the compound was placed in a small vial, 0.5 mL of deionized H_2O was added and the vial was closed. The vial and its contents were sonicated for 5 minutes at room temperature, vigorously shaken and left for one hour at room temperature. This was repeated six times. The vial was then left for next 12 hours at room temperature. Supernatant was then collected and filtered through a 0.22 µm syringe filter. The solution obtained this way was considered saturated. It was diluted 10 times (**4**), 50 times (**1**) or 100 times (**2**, **3**). Absorbance at the respective absorption maximum was measured (**1** – 290 nm, **2** -278 nm, **3** – 275 nm, **4** – 269 nm). Solubility (concentration of the saturated solution) was calculated based on the applied dilution, Lambert-Beer equation and molar absorption coefficient obtained beforehand.

Cation binding experiments (qualitive)

Cation binding experiments between compounds **1-4** (chemosensors) and cations (analytes; Li^{+}, Na^{+}, Cs^{+}, NH_4^{+}, Mg^{2+}, Zn^{2+}, Cu^{2+}, Ni^{2+}, Co^{2+}, La^{3+}, Eu^{3+}, Al^{3+}, Ga^{3+}, In^{3+}, Sn^{4+}, Cr^{3+}, Sc^{3+}) were performed employing the emission spectra measurements. Cations were introduced in the form of their corresponding chloride salts. The experiments were performed as follows. In a 5 mL volumetric flask, stock solutions of **1-4** (10 μM) were diluted with adequate volume of deionized H_2O to reach volume of approximately 3 mL. 0.5 mL of solution of AcOH/Tris buffer (10 mM) was added. Appropriate volume of stock solution of cation (0.1 mM) was added. H_2O was added to reach the final volume of 5 mL. Final concentration of **1-4** in each sample equaled 0.10 μM. Final concentration of cation in each sample equaled 5.0 μM. Excitation wavelength (λ_{ex}) for corresponding receptors were as follows: 289 nm (**1**), 278 nm (**2**), 271 nm (**3**), 266 nm (**4**). Fluorescence intensity data was collected at the following emission wavelengths: 390 nm (**1**), 375 nm (**2**), 376 nm (**3**), 383 nm (**4**).

Cation binding experiments (quantitive)

Cation binding experiments between compounds **1**, **3** and **4** (chemosensors) and chosen cations (analytes; Al^{3+}, Ga^{3+}, In^{3+}) were performed employing the emission spectra measurements. Cations were introduced in the form of their corresponding chloride salts. The experiments were performed as follows. In a 5 mL volumetric flask, stock solutions of **1**, **3** or **4** (10 μM) was diluted with adequate volume of deionized H_2O to reach volume of approximately 3 mL. 0.5 mL of solution of AcOH/Tris buffer (10 mM) was added. Appropriate volume of stock solution of cation (0.01 mM, 0.1 mM or 1 mM) was added. H_2O was added to reach the final volume of 5 mL. Final concentration of **1-4** in each sample equaled 0.10 μM. Excitation wavelength (λ_{ex}) for corresponding receptors were as follows: 289 nm (**1**), 278 nm (**2**), 271 nm (**3**), 266 nm (**4**). Fluorescence intensity data was collected at the following emission wavelengths: 390 nm (**1**), 375 nm (**2**), 376 nm (**3**), 383 nm (**4**). For chosen receptor-cation pairs, modified Stern–Volmer plots were used for the evaluation of detection parameters, namely Stern–Volmer constants (K_{SV}), average numbers of binders (n) and limit of detection (LOD) values.[9] This was done by fitting the binding data to the modified Stern-Volmer equation (**Equation S1**).[10–12] Fitting was done using "Simple Fit" functionality of Origin2024 software.

Equation S1. Modified Stern-Volmer equation used to describe the quenching. I_0 – fluorescence of the receptor before adding the cation, I – fluorescence after adding the cation, K_{SV} – Stern-Volmer constant, n – number of molecules bound to one receptor, c_{ion} – ion concentration.

$$log\left(\frac{I_0 - I}{I}\right) = log(K_{SV}) + n \cdot log(c_{ion})$$

Cation binding experiments (binding site determination)

20 mM stock solutions of **5b** and $AlCl_3$ in D_2O were prepared by dissolving appropriate quantities of these compounds in D_2O. Two NMR samples were then prepared. Sample 1 was prepared by mixing 250 μL **5b** stock solution and 250 μL of D_2O. Sample 2 was prepared by mixing 250 μL of **5b** stock solution and 250 μL **5b** $AlCl_3$ stock solution. $^{13}C\{^1H\}$ NMR spectra of both solutions were then measured.

Cation binding experiments (real-life samples)

Samples were collected on August 15th, 2024 in the locations indicated below (Table S1). After being collected, samples were filtered through a 0.5 μm syringe filter and subjected to analyzes.

Absorption and fluorescence spectra of each sample were measured as follows. To a 5 mL volumetric flask, 500 μL of 10 mM AcOH/Tris pH 5.0 buffer was added. 100 μL of distilled water was also added. The flask was filled with the respective water sample to obtain the total volume of 5 mL. Absorption spectra of each sample were then measured (Plot S4). Fluorescence spectra of each sample at 289 nm excitation wavelength were also measured (Plot S5).

Fluorescence of **1** in real-life water samples was measured as follows. To a 5 mL volumetric flask, 500 μL of 10 mM AcOH/Tris pH 5.0 buffer was added. 50 μL of 10 μM stock solution of **1** was also added. 50 μL of distilled water was then added. The flask was filled with the respective water sample to obtain the total volume of 5 mL. Fluorescence intensity of the sample at 289 nm excitation wavelength and 390 nm emission wavelength was then measured. Fluorescence of the sample without **1** (Plot S5) was subtracted from the obtained value.

Fluorescence of **1** in real-life water samples in presence of 5 μM Al^{3+} was measured as follows. To a 5 mL volumetric flask, 500 μL of 10 mM AcOH/Tris pH 5.0 buffer was added. 50 μL of 10 μM stock solution of **1** was also added. 50 μL of 0.5 mM $AlCl_3$ stock solution in water was then added. The flask was filled with the respective water sample to obtain the total volume of 5 mL. Fluorescence intensity of the sample at 289 nm excitation wavelength and 390 nm emission wavelength was then measured. Fluorescence of the sample without **1** (Plot S5) was subtracted from the obtained value.

Table S1. Locations where real-life samples were collected.

Sample number	Geographic coordinates	Location name	Location description
**1**	34°49'21.0"N 135°31'06.1"E	Zuion'ike Pond	urban retention pond
**2**	35°00'04.8"N 135°53'32.7"E	Biwa Lake	large lake
**3**	35°12'14.3"N 135°52'40.3"E	Kojorougaike Pond	eutrophic mountain pond
**4**	35°11'35.2"N 135°53'22.7"E	Kusushinotaki Waterfall	mountain waterfall
**5**	35°11'57.7"N 135°52'54.2"E	-	mountain spring
**6**	35°12'04.5"N 135°53'15.6"E	Nohanarene River	mountain stream near its source

Molecular dynamics – general methodology

GROMACS software version 2023.4 was used in the simulations.[13,14] Starting geometries of molecules were constructed using Avogadro software, version 1.2.0.[15] Sobtop tool[16] was used to parametrize compounds **1-4** (GAFF forcefield[17], EEM charges[18]). SPC/E water model was used.[19] Short-range electrostatic interactions cut-off was applied at 1.0 nm. Particle-mesh Ewald method was applied for long-range electrostatic interactions.[20,21] Short-range dispersion interactions cut-off was applied at 1.0 nm and long range dispersion corrections for energy and pressure were applied. The temperature was set to 298 K. Bussi-Donadio-Parinello (v-rescale) thermostat with 0.1 ps time constant was used.[22] Parrinello-Rahman barostat was used with isotropic coupling, 2.0 ps time constant, reference pressure set to 1.0 bar and compressibility set to $4.5\cdot10^{-5}\cdot bar^{-1}$.[23] Periodic boundary conditions were used in all directions. Simulation step was set to 2 fs, and leap-frog integrator was used in the simulations.

Free energy of dimerization calculations

For sumanene derivatives (**1** and **2**), geometries of three types of dimers were independently constructed (a: convex-to-concave, b: convex-to-convex, c: concave-to-concave). For triphenylene derivatives (**3** and **4**) geometry of one type of dimer was constructred. In each case, a dimer was placed in a rectangular box (5x5x7 nm). Solvent was added. Energy of the system was minimized. Reaction coordinate was defined as the distance between molecular cores (only carbon atoms) of monomers, in Z direction only. Additionally, the distance between the monomers was constrained in X and Y directions by an umbrella potential (500 $kJ\cdot mol^{-1}\cdot nm^{-2}$) in each simulation. Initial pulling was performed (0.6 ns, 0.0 -> 3.0 nm, 500 $kJ\cdot mol^{-1}\cdot nm^{-2}$). Simulation frames were extracted from the initial trajectory (one frame every 0.1 nm, from 0.0 nm to 3.0 nm). Independent simulations were performer for each of these frames (umbrella potential constant: 2000 $kJ\cdot mol^{-1}\cdot nm^{-2}$, 11 ns, first 1 ns treated as equilibration time and discarded). Free energy landscape was calculated from the sampling data using WHAM method.[24,25] Error was estimated using bootstrapping. Dimerization energy was determined as the value of the lowest minimum on the free energy of dimerization profile. For sumanene derivatives, lowest value obtained for all three dimers was used. Calculated free energy profiles were provided in the SI section "S7. Free energy of dimerization profiles".

Cation structure modelling

Two Al^{3+} cations and one anion of **1** (fully ionized, hexaanion) was placed in a 5x5x5 nm rectangular box. The following nonbonded parameters were used for Al^{3+} ions: charge: 3.0, mass: 26.98, sigma: $1.94126\cdot 10^{-1}$, epsilon: $9.77218\cdot 10^{-3}$.[26] 32 independent MD simulations of the system were performed. The structures resulting from the simulations were visually inspected. They could be grouped in 3 groups: a) both Al^{3+} cations on the same (convex) side of the sumanene bowl, b) both Al^{3+} cations on the same (concave) side of the sumanene bowl, c) Al^{3+} cations on the different sides of the sumanene bowl. For each of these groups, a representative structure was chosen. Water molecules were deleted so that only 3 water molecules closest to each Al^{3+} cation remained. Each of the 3 structures was then optimized using 3-corrected Hartree-Fock method (HF-3c)[27] with CPCM water model.[28] Orca 6.0.0 software was used.[29] The energies of the complexes were compared. Lowest-energy structure was assumed to be the prevalent complex structure.

S2. Synthesis

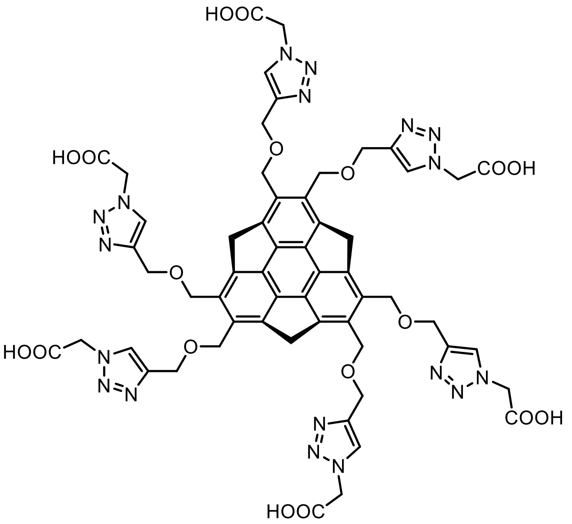

2,2',2",2"',2"",2""'-(4,4',4",4"',4"",4""'-((((4,7-dihydro-1H-Tricyclopenta[def,jkl,pqr]triphenylene-2,3,5,6,8,9-hexayl)hexakis(methylene))hexakis(oxy))hexakis(methylene))hexakis(1H-1,2,3-triazole-4,1-diyl))hexaacetic acid 1

Alkyne **6** (8.4 mg, 1.0 eq), copper(I) thiophene-2-carboxylate (1.4 mg, 0.6 eq), DMSO (1.0 ml), azidoacetic acid (11.2 µl, 12 eq) and triethylamine (22.6 µl, 13 eq) were sealed in a microwave vial under nitrogen flow. The mixture was heated at 80 °C for 20 minutes using microwave radiation. When the mixture cooled down, 5 mL of 5% Na_2CO_3 (aq) was added. The mixture was washed with 5 x 10 ml of AcOEt. The pH of water phase was then adjusted to pH = 2 using concentrated HCl (aq). After 10 minutes, the precipitate was separated by membrane filtration, washed with 3 x 1 ml of 1 % HCl (aq) and with 1 ml of deionized water. The product was then dried *in vacuo* to afford 12.7 mg (80%) of a green powder contaminated by Cu^{2+}. The green powder was dissolved in 1 ml of 5% Na_2CO_3 (aq). A solution of $Na_2S \cdot 9\ H_2O$ (23.9 mg, 10 eq) in 0.5 ml of deionized water was added, upon which a black precipitate appeared. The mixture was left for 30 minutes without stirring. It was then filtered through a syringe filter (pore diameter: 0.22 µm). The pH was adjusted to pH = 2 using concentrated HCl (aq). After 10 minutes, the precipitate was separated by membrane filtration, washed with 3 x 1 ml of 1 % HCl (aq) and 5 x 1 ml of deionized water. The product was then dried *in vacuo* (60 Pa) to afford **1** (9.2 mg, 61%) of as a yellow powder (< 0.5 mol % Cu^{2+}, measured by ICP-AES).

^{1}H NMR (400 MHz, DMSO-*d*$_6$) δ 8.09 (s, 6H), 5.24 (s, 12H), 4.51-4.73 (m, 27H), 3.59 (d, $^{3}J_{HH}$ = 20.2 Hz, 3H).

^{13}C{^{1}H} NMR (101 MHz, DMSO-*d*$_6$) δ 168.6, 149.3, 147.1, 144.0, 133.0, 125.4, 67.1, 62.9, 50.4.

^{1}H DOSY NMR (400 MHz, DMSO-*d*$_6$) *D* $9.82 \cdot 10^{-11}$ m^2/s .

MALDI-HRMS m/z calcd. for $C_{57}H_{54}N_{18}O_{18}$ $[M\text{-}H]^-$ 1277.3785; found 1277.3791

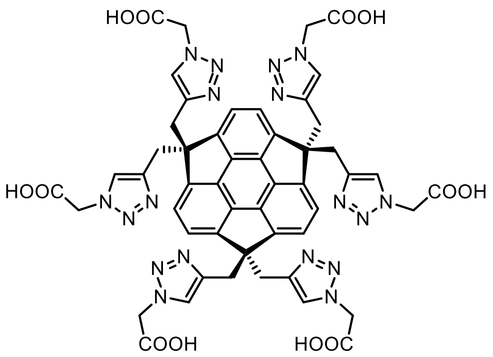

2,2',2'',2''',2'''',2'''''-(4,4',4'',4''',4'''',4'''''-((4,7-Dihydro-1H-tricyclopenta[def,jkl,pqr]triphenylene-1,1,4,4,7,7-hexayl)hexakis(methylene))hexakis(1H-1,2,3-triazole-4,1-diyl))hexaacetic acid 2

Prepared and isolated analogously to **1**. 9.2 mg of alkyne **8** was used. The product (8.2 mg, 40%) was obtained as an off-white powder (< 0.5 mol % Cu^{2+}, measured by ICP-AES).

^{1}H NMR (400 MHz, DMSO-*d6*) δ 7.98 (s, 3H), 6.69 (s, 6H), 6.65 (s, 3H), 5.26 (s, 6H), 4.93 (s, 6H), 3.80 (s, 6H), 2.78 (s, 6H).

$^{13}C\{^{1}H\}$ NMR (101 MHz, DMSO-*d6*) δ 168.8, 168.5, 154.2, 145.2, 143.4, 142.9, 125.3, 124.3, 123.6, 62.4, 50.4, 50.3, 34.1, 31.3.

^{1}H DOSY NMR (400 MHz, DMSO-*d6*) D $2.04 \cdot 10^{-10}$ m^2/s .

MALDI-HRMS m/z calcd. for $C_{51}H_{42}N_{18}O_{12}$ $[M-H]^{-}$ 1097.3157; found 1097.3127

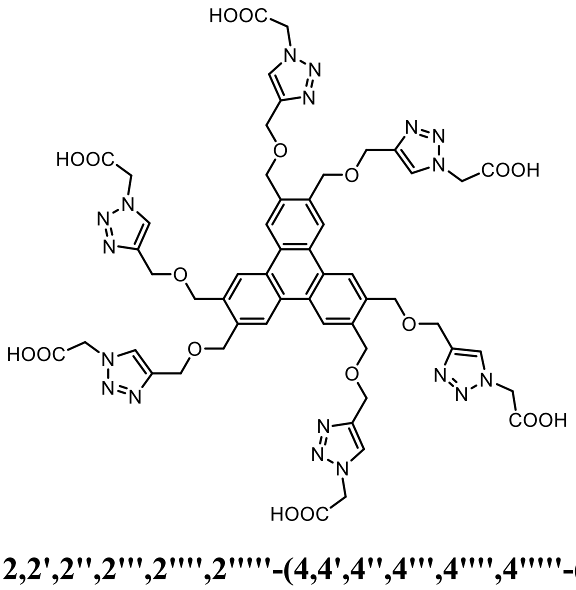

2,2',2'',2''',2'''',2'''''-(4,4',4'',4''',4'''',4'''''-(((Triphenylene-2,3,6,7,10,11-hexaylhexakis(methylene))hexakis(oxy))hexakis(methylene))hexakis(1H-1,2,3-triazole-4,1-diyl))hexaacetic acid 3

Prepared and isolated analogously to **1**. 4.0 mg of alkyne **11** was used. The product (2.1 mg, 26%) was obtained as a beige powder (< 0.5 mol % Cu^{2+}, measured by ICP-AES).

^{1}H NMR (400 MHz, DMSO-*d6*) δ 8.81 (s, 6H), 8.18 (s, 6H), 5.29 (s, 12H), 4.87 (s, 12H), 4.71 (s, 12H).

$^{13}C\{^1H\}$ NMR (101 MHz, DMSO-d_6) δ 169.2, 144.4, 136.6, 128.8, 126.1, 124.3, 69.9, 63.7, 51.0.

^1H DOSY NMR (400 MHz, DMSO-d_6) D $9.82 \cdot 10^{-11}$ m^2/s.

MALDI-HRMS m/z calcd. for $C_{54}H_{54}N_{18}O_{18}$ $[M]^+$ 1242.3869; found 1242.3870

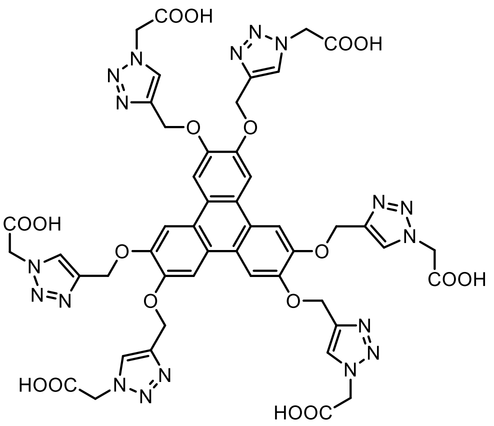

2,2',2'',2''',2'''',2'''''-(4,4',4'',4''',4'''',4'''''-((Triphenylene-2,3,6,7,10,11-hexaylhexakis(oxy))hexakis(methylene))hexakis(1H-1,2,3-triazole-4,1-diyl))hexaacetic acid 4

Prepared and isolated analogously to **1**. 20.7 mg of alkyne **11** was used. The product (42.6 mg, 98%) was obtained as a grey powder (< 0.5 mol % Cu^{2+}, measured by ICP-AES).

^1H NMR (400 MHz, DMSO-d_6) δ 8.35 (s, 6H), 8.33 (s, 6H), 5.51 (s, 12H), 5.29 (s, 12H).

$^{13}C\{^1H\}$ NMR (101 MHz, DMSO-d_6) δ 168.6, 147.6, 142.7, 126.4, 123.0, 107.2, 61.7, 50.7.

^1H DOSY NMR (400 MHz, DMSO-d_6) D $8.81 \cdot 10^{-11}$ m^2/s.

MALDI-HRMS m/z calcd. for $C_{48}H_{42}N_{18}O_{18}$ $[M-H]^+$ 1157.2841; found 1157.2838

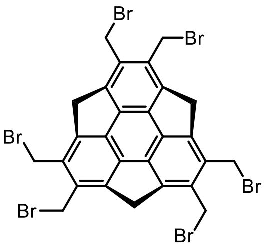

2,3,5,6,8,9-Hexakis(bromomethyl)-4,7-dihydro-1H-tricyclopenta[def,jkl,pqr]triphenylene 6

Prepared from pristine sumanene following the procedure reported by Sakurai *et al.*[30] **5** was obtained as a yellow powder (309.0 mg, 99%).

^1H NMR (400 MHz, $CDCl_3$) δ 4.79 (ABq-d, J_{AB} = 14.1 Hz, $^2J_{HH}$ = 11.6 Hz, 12H), 4.70 (d, $^2J_{HH}$ = 19.4 Hz, 3H), 3.80 (d, $^2J_{HH}$ = 19.4 Hz, 3H).

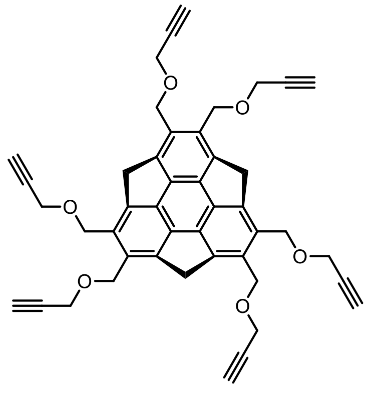

2,3,5,6,8,9-Hexakis((prop-2-yn-1-yloxy)methyl)-4,7-dihydro-1H-tricyclopenta[def,jkl,pqr]triphenylene 7

A mixture of **5** (100.0 mg, 1.0 eq), *n*-Bu_2SnO (200 mg, 6.6 eq) and propargyl alcohol (4.0 ml) was heated in 80 °C for 3 hours in N_2 atmosphere. The mixture was then cooled down, quenched with 30 ml H_2O and extracted with 3 x 30 ml DCM. It was then desiccated with Na_2SO_4 and filtered. Solvent and volatile fractions were removed *in vacuo*. The mixture was purified by PTLC (ethyl acetate : hexane 1:2) to afford **6** (65.7 mg, 82%) as a yellow solid.

^{1}H NMR (400 MHz, $CDCl_3$) δ 4.69-4.86 (m, 15H), 4.20 (ABq-d, J_{AB} = 33.5 Hz, $^2J_{HH}$ = 16.0 Hz, $^4J_{HH}$ = 2.3 Hz, 12H) 3.74 (d, $^2J_{HH}$ = 19.6 Hz, 3H), 2.50 (t, $^4J_{HH}$ = 2.3 Hz, 6H).

^{13}C{^{1}H} NMR (101 MHz, $CDCl_3$) δ 150.2, 148.4, 132.2, 80.1, 74.9, 67.2, 57.3, 41.1.

EI-HRMS m/z calcd. for $C_{45}H_{36}O_6$ $[M]^+$ 672.2512; found 672.2528

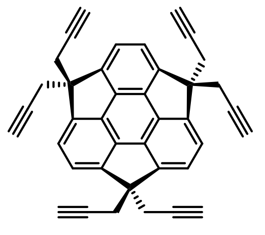

1,1,4,4,7,7-Hexa(prop-2-yn-1-yl)-4,7-dihydro-1H-tricyclopenta[def,jkl,pqr]triphenylene 9

Prepared using a procedure similar to the one reported by Hirao *et al.*[31] Pristine sumanene (**7**, 30.0 mg, 1 eq), tetrabutylammonium bromide (43.8 mg, 1.2 eq) and propargyl bromide (102 µl, 12 eq) were suspended in THF (1.0 ml). 4 ml of 30% NaOH (*aq*) was added. The mixture was stirred for 46 hours in RT in N_2 atmosphere. The mixture was quenched with 20 ml H_2O, extracted with 3 x 20 ml DCM, desiccated with Na_2SO_4 and filtered. Solvent and volatile fractions were removed *in vacuo*. The mixture was purified by PTLC ($CHCl_3$: hexane 3:2) to afford **9** (49.0 mg, 88%) as a yellow solid.

^{1}H NMR (400 MHz, $CDCl_3$) δ 7.32 (s, 6H), 3.45 (d, $^4J_{HH}$ = 2.5 Hz, 6H), 2.50 (d, $^4J_{HH}$ = 2.5 Hz, 6H), 2.22 (t, $^4J_{HH}$ = 2.5 Hz, 3H), 2.07 (t, $^4J_{HH}$ = 2.5 Hz, 3H).

$^{13}C\{^1H\}$ NMR (101 MHz, $CDCl_3$) δ 154.9, 146.0, 122.9, 81.20, 81.1, 71.6, 71.3, 59.5, 28.8, 24.6.

EI-HRMS m/z calcd. for $C_{39}H_{24}$ $[M]^+$ 492.1878; found 492.1875

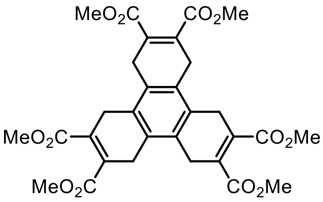

Hexamethyl 1,4,5,8,9,12-hexahydrotriphenylene-2,3,6,7,10,11-hexacarboxylate.

Prepared from hexakis(bromomethyl)benzene (2.00 g) following the procedure reported by Fukushima *et al.*[32] Hexamethyl 1,4,5,8,9,12-hexahydrotriphenylene-2,3,6,7,10,11-hexacarboxylate (123.9 mg, 6.8%) was obtained as a white solid and used in the next step without further purification.

^1H NMR (400 MHz, $CDCl_3$) δ 3.85 (s, 18H), 3.55 (s, 12H).

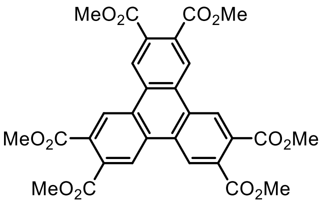

Hexamethyl triphenylene-2,3,6,7,10,11-hexacarboxylate 10

Prepared from hexamethyl 1,4,5,8,9,12-hexahydrotriphenylene-2,3,6,7,10,11-hexacarboxylate following the procedure similar to the one reported by Fukushima *et al.*[32] Hexamethyl 1,4,5,8,9,12-hexahydrotriphenylene-2,3,6,7,10,11-hexacarboxylate (123.9 mg, 1.0 eq) and MnO_2 (495.6 mg, 27 eq) were suspended in toluene (31 ml). The mixture was stirred in 65 °C for 16 h. It was then cooled and filtered through a SiO_2 plug. The plug was washed with 5% MeOH in $CHCl_3$. Volatile fractions were removed from the filtrate *in vacuo*, yielding a solid residue. The residue was purified by PTLC (MeOH : $CHCl_3$ 2:98) to afford **10** (80.2 mg, 65%) as a white solid.

^1H NMR (400 MHz, $CDCl_3$) δ 9.02 (s, 6H), 4.05 (s, 18H).

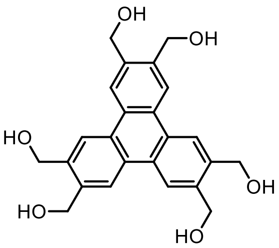

Triphenylene-2,3,6,7,10,11-hexaylhexamethanol 11

9 (20.2 mg, 1.0 eq) was put in a flame-dried microwave vial. The vial was then evacuated and filled with N_2. Dry THF (1 ml) was added, and the vial was cooled to 0 °C. A solution of $LiAlH_4$ (16.0 mg, 12 eq) in dry THF (2 ml) was then slowly added. The mixture was heated in 80 °C for 2 hours using microwave radiation. The mixture was quenched with 5 ml 10% HCl (*aq*), upon which a white precipitate formed. The precipitate was separated on a membrane filter and washed with 10 ml 10 % NaOH (aq) and with 20 ml of deionized H_2O. It was then dried *in vacuo* (60 Pa) to afford **11** (8.0 mg, 56%) as a white solid.

^1H NMR (400 MHz, DMSO-d_6) δ 8.75 (s, 6H), 5.37 (t, $^3J_{HH}$ = 5.4 Hz, 6H), 4.81 (d, $^3J_{HH}$ = 5.4 Hz, 12H).

$^{13}C\{^1H\}$ NMR (101 MHz, DMSO-d_6) δ 138.7, 127.7, 121.1, 60.6.

MALDI-HRMS m/z calcd. for $C_{24}H_{24}O_6$ $[M+Na]^+$ 431.1465; found 431.1465

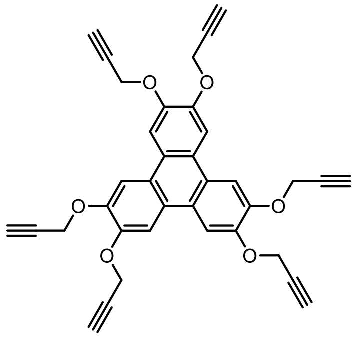

2,3,6,7,10,11-Hexakis((prop-2-yn-1-yloxy)methyl)triphenylene 12

Prepared using a procedure similar to the one reported by Sakamoto *et al.*[33] Compound **11** (8.0 mg, 1 eq), KOH (13.2 mg, 12 eq) and propargyl bromide (26.5 µl, 18 eq) were suspended in DMSO (0.5 ml). The mixture was stirred for 2 hours in RT in N_2 atmosphere, after which TLC analysis revealed that the reaction was not complete. Propargyl bromide (26.5 µl, 18 eq) was added and the reaction was continued for 1 hour in RT in N_2 atmosphere. The reaction was then quenched with 10 ml H_2O. The precipitate was separated by membrane filtration, washed on the filter using 20 ml of deionized H_2O and dried *in vacuo*. It was then purified by PTLC (MeOH : $CHCl_3$ 2:98) to yield **12** (4.0 mg, 32%) as a yellow solid.

^1H NMR (400 MHz, $CDCl_3$) δ 8.68 (s, 6H), 4.96 (s, 12H), 4.32 (d, $^4J_{HH}$ = 2.3 Hz, 12H), 2.55 (t, $^4J_{HH}$ = 2.3 Hz, 6H).

$^{13}C\{^1H\}$ NMR (101 MHz, $CDCl_3$) δ 135.1, 129.4, 124.6, 79.9, 75.1, 69.7, 57.7.

ESI-HRMS m/z calcd. for $C_{42}H_{36}O_6$ $[M+Na]^+$ 659.2404; found 659.2403

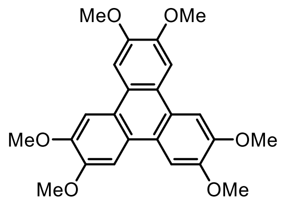

2,3,6,7,10,11-Hexamethoxytriphenylene

Prepared from veratrole following the procedure reported by Guillon *et al.*[34] 2,3,6,7,10,11-Hexamethoxytriphenylene was obtained as a white solid (1096 mg, 81%).

^{1}H NMR (400 MHz, $CDCl_3$) δ 7.83 (s, 6H), 4.14 (s, 18H).

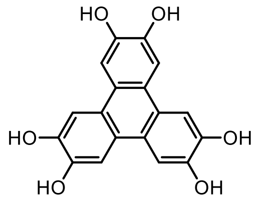

2,3,6,7,10,11-Hexahydroxytriphenylene 13

Prepared from 2,3,6,7,10,11-hexamethoxytriphenylene following the procedure reported by Zhang *et al.*[35] **13** was obtained as a purple solid (407.3 mg, 99%).

^{1}H NMR (400 MHz, DMSO-d_6) δ 9.28 (s, 6H), 7.60 (s, 6H).

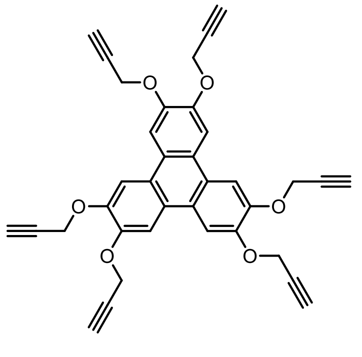

2,3,6,7,10,11-Hexakis(prop-2-yn-1-yloxy)triphenylene 14

A Schlenk vial was flame-dried, evacuated and filled with N_2. Compound **13** (100.0 mg, 1 eq), K_2CO_3 (383.6 mg, 9 eq) and dry DMF (5 ml) were introduced into the flask. Propargyl bromide (210.3 µl, 9 eq) was then added. The mixture was stirred for 8 days in RT in N_2 atmosphere. H_2O (30 ml), AcOEt (15 ml) and hexane (15 ml) were then added to the mixture, upon which a white precipitate of **14** appeared. The precipitate was separated on a membrane filter and washed with H_2O (30 ml), AcOEt (10 ml) and hexane (30 ml). It was then dried *in vacuo* to afford **14** (140.0 mg, 82%) as an off-white solid.

^{1}H NMR (400 MHz, DMSO-d_6) δ 8.15 (s, 6H), 5.10 (d, $^4J_{HH}$ = 2.3 Hz, 12H), 3.61 (t, $^4J_{HH}$ = 2.3 Hz, 6H).

$^{13}C\{^1H\}$ NMR (101 MHz, DMSO-d_6) δ 146.9, 123.23, 107.9, 79.1, 78.7, 56.5.

EI-HRMS m/z calcd. for $C_{36}H_{24}O_6$ $[M]^+$ 552.1573; found 552.1579

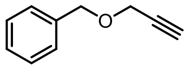

Prop-2-ynoxymethylbenzene 16

Prepared using a procedure similar to the one reported by Boger *et al.*[36] Benzyl alcohol (104 µl, 1.0 eq), KOH (100 mg, 1.8 eq) and propargyl bromide (91 µl, 1.2 eq) were dissolved in DMSO (1.0 ml). The mixture was stirred for 16 hours in RT in N_2 atmosphere. It was then quenched with 20 ml H_2O and extracted with 3 x 20 ml diethyl ether. The extract was washed with brine 3 times, dessicated over Na_2SO_4 and carefully evaporated *in vacuo*. It was then purified by PTLC (hexane : AcOEt 1:1) to afford prop-2-ynoxymethylbenzene (118.4 mg, 81 %) as a yellow oil. prop-2-ynoxymethylbenzene was used in the next step without further purification.

^1H NMR (400 MHz, $CDCl_3$) δ 8.15 (s, 6H), 5.10 (d, $^4J_{HH}$ = 2.3 Hz, 12H), 3.61 (t, $^4J_{HH}$ = 2.3 Hz, 2H), 2.47 (t, $^4J_{HH}$ = 2.3 Hz, 1H).

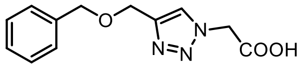

2-(4-((Benzyloxy)methyl)-1H-1,2,3-triazol-1-yl)acetic acid 5

Prop-2-ynoxymethylbenzene (61.0 mg, 1.0 eq), CuTC (8.0 mg, 0.1 eq), DMSO (2.0 ml), azidoacetic acid (62.5 µl, 2.0 eq) and triethylamine (128 µl, 2.2 eq) were sealed in a microwave vial under nitrogen flow. The mixture was heated in 80 °C for 20 minutes using microwave radiation. When the mixture cooled down, 10 mL of 5% Na_2CO_3 (aq) was added. The mixture was washed with 5 x 10 ml of AcOEt. The pH of water phase was then adjusted to pH = 2 using concentrated HCl (*aq*). No precipitate appeared. The mixture was extracted with 3 x 10 ml $CHCl_3$. The extract was dessicated over Na_2SO_4. Volatile fractions were removed *in vacuo*. The obtained residue was recrystallized from 4.0 ml of deionized water with hot filtration, washed with cold deionized H_2O and dried to afford **5** (48.3 mg, 47%) as colorless prismatic crystals (m.p. = 128.5 – 129.5 °C; < 0.5 mol % Cu^{2+}, measured by ICP-AES).

^1H NMR (400 MHz, DMSO-d_6) δ 8.11 (s, 1H), 7.25-7.40 (m, 5H), 5.26 (s, 2H), 4.58 (s, 2H), 4.53 (s, 2H).

$^{13}C\{^1H\}$ NMR (101 MHz, DMSO-d_6) δ 168.7, 143.9, 138.2, 128.3, 127.7, 127.6, 125.4, 71.3, 62.8, 50.5.

^1H DOSY NMR (400 MHz, DMSO-d_6) D $2.48 \cdot 10^{-10}$ m^2/s.

ESI-HRMS m/z calcd. for $C_{12}H_{13}N_3O_3Na$ $[M+Na]^+$ 270.0849; found 270.0854

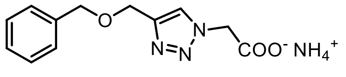

Ammonium 2-(4-((benzyloxy)methyl)-1H-1,2,3-triazol-1-yl)acetate 5b

2-(4-((Benzyloxy)methyl)-1H-1,2,3-triazol-1-yl)acetic acid **5** was dissolved in MeOH (1 ml). 14.8 M NH_3 (aq) (10.0 µl, 4.0 eq) was added. The mixture was stirred in RT for 5 minutes. Volatile fractions were removed *in vacuo*. **5b** was obtained as a white solid (10.5 mg, 100%), pH of water solution: 7.

^{1}H NMR (400 MHz, D_2O, PreSAT water supression) δ 8.02 (s, 1H), 7.43-7.52 (m, 5H), 5.10 (s, 2H), 4.82 (s, 2H), 4.70 (s, 2H).

^{13}C{^{1}H} NMR (101 MHz, D_2O) δ 173.3, 143.9, 137.0, 128.8, 128.8, 128.5, 126.2, 72.8, 62.4, 53.2.

S3. Spectral characterization data for compounds 1-14

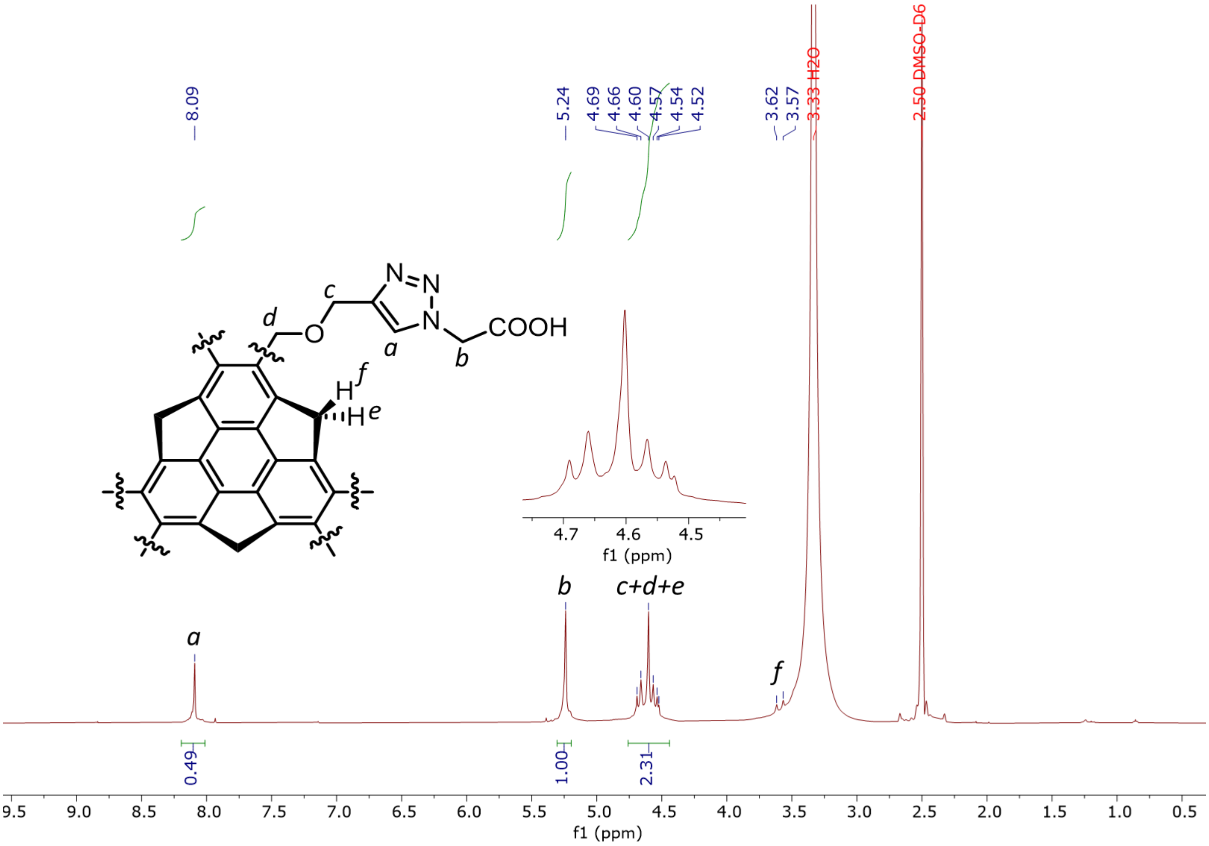

Figure S1. ^{1}H NMR spectrum of compound **1** (400 MHz, DMSO-d_6) with signals assigned.

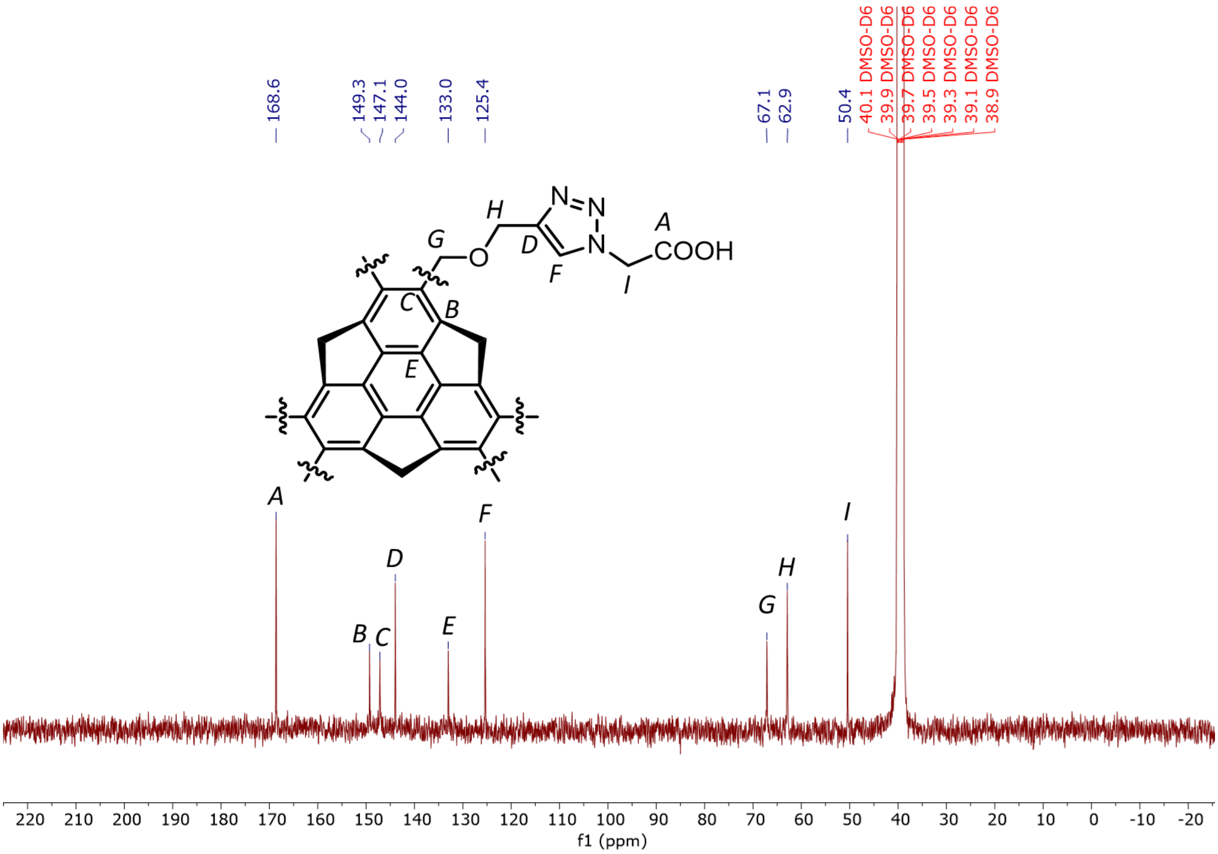

Figure S2. ^{13}C NMR spectrum of compound **1** (101 MHz, DMSO-d_6) with signals assigned.

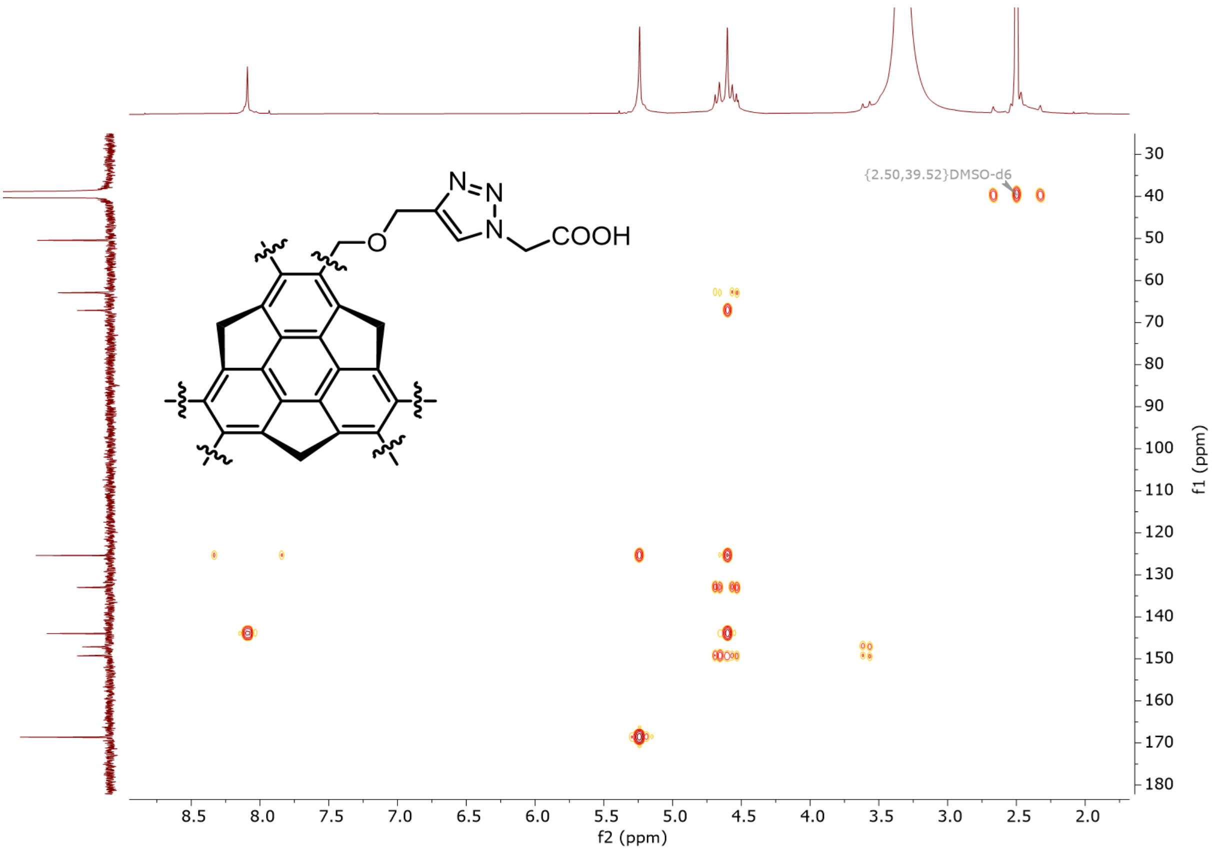

Figure S3 ^{1}H-^{13}C HMBC spectrum of compound **1** (DMSO-d_6).

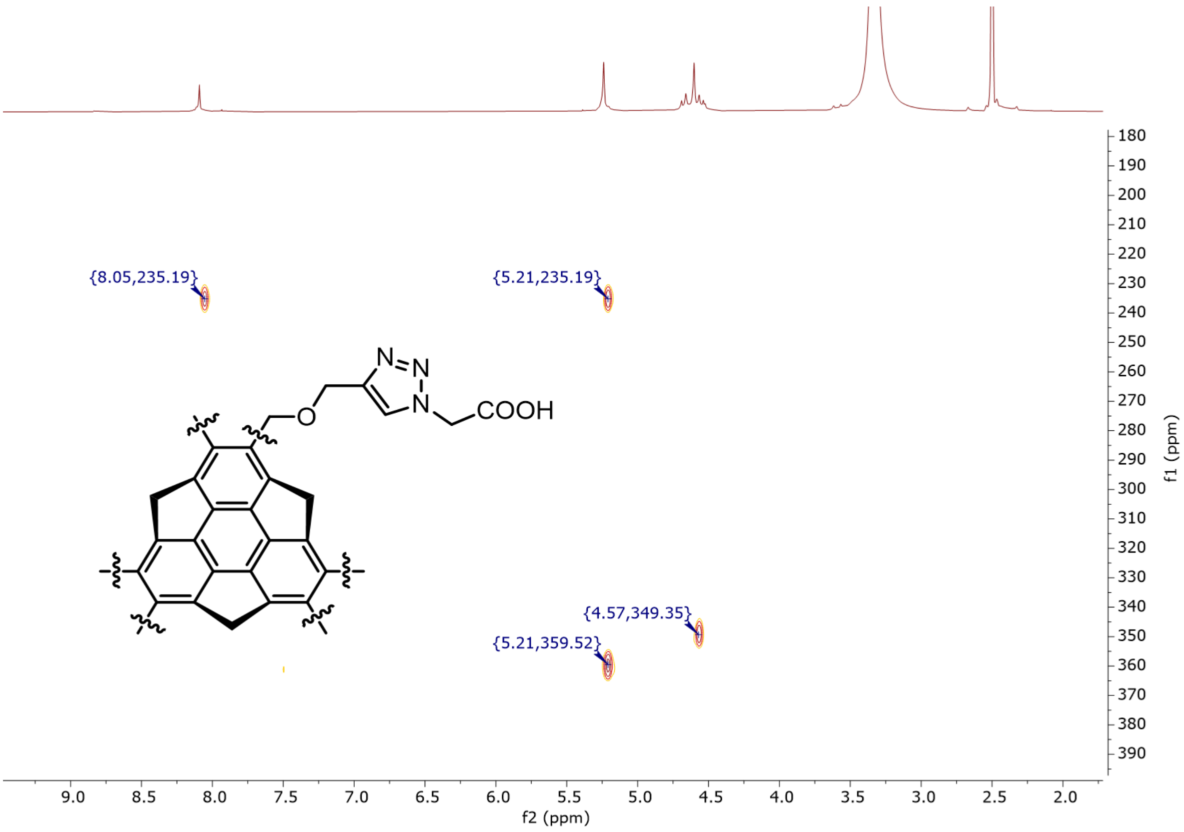

Figure S4. ^{1}H-^{15}N HMBC spectrum of compound **1** (DMSO-d_6).

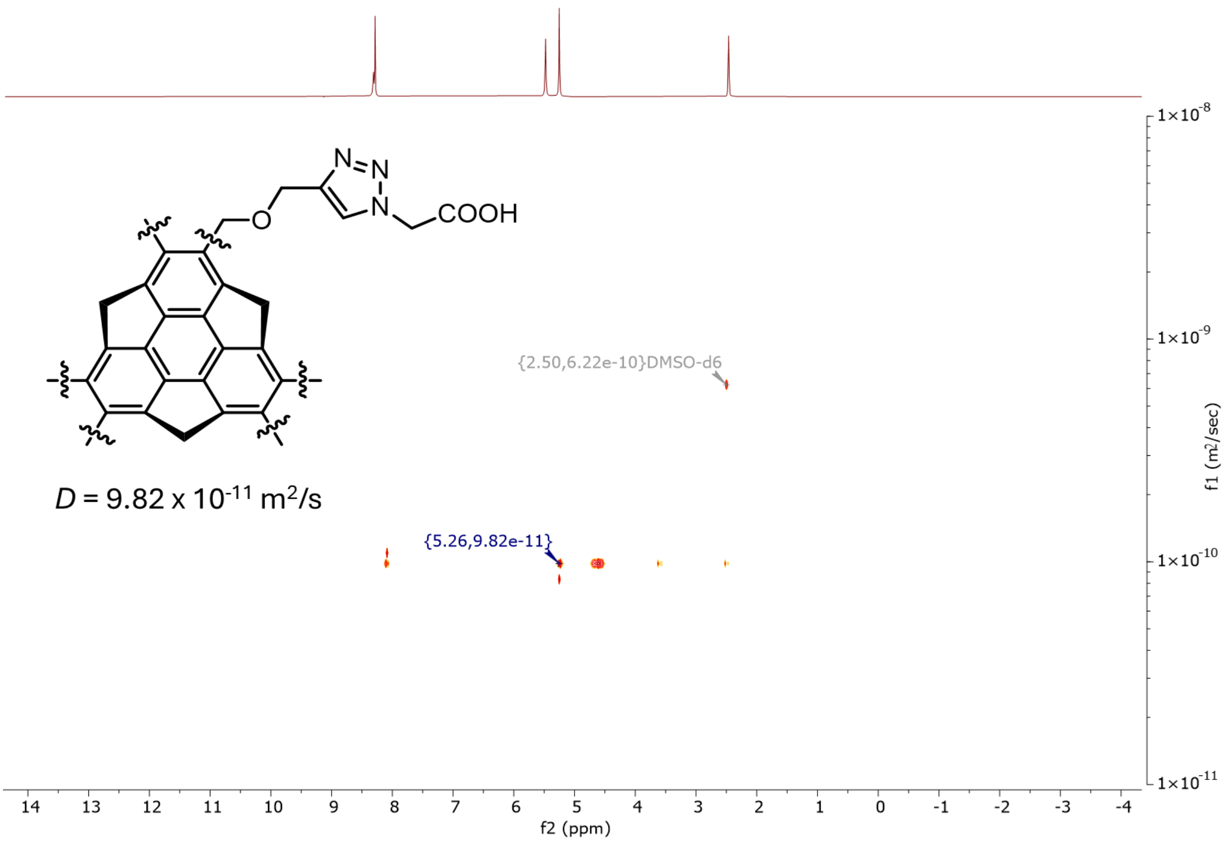

Figure S5. DOSY NMR spectrum of compound **1** (400 MHz, DMSO-d_6, 30 °C, 10 mM).

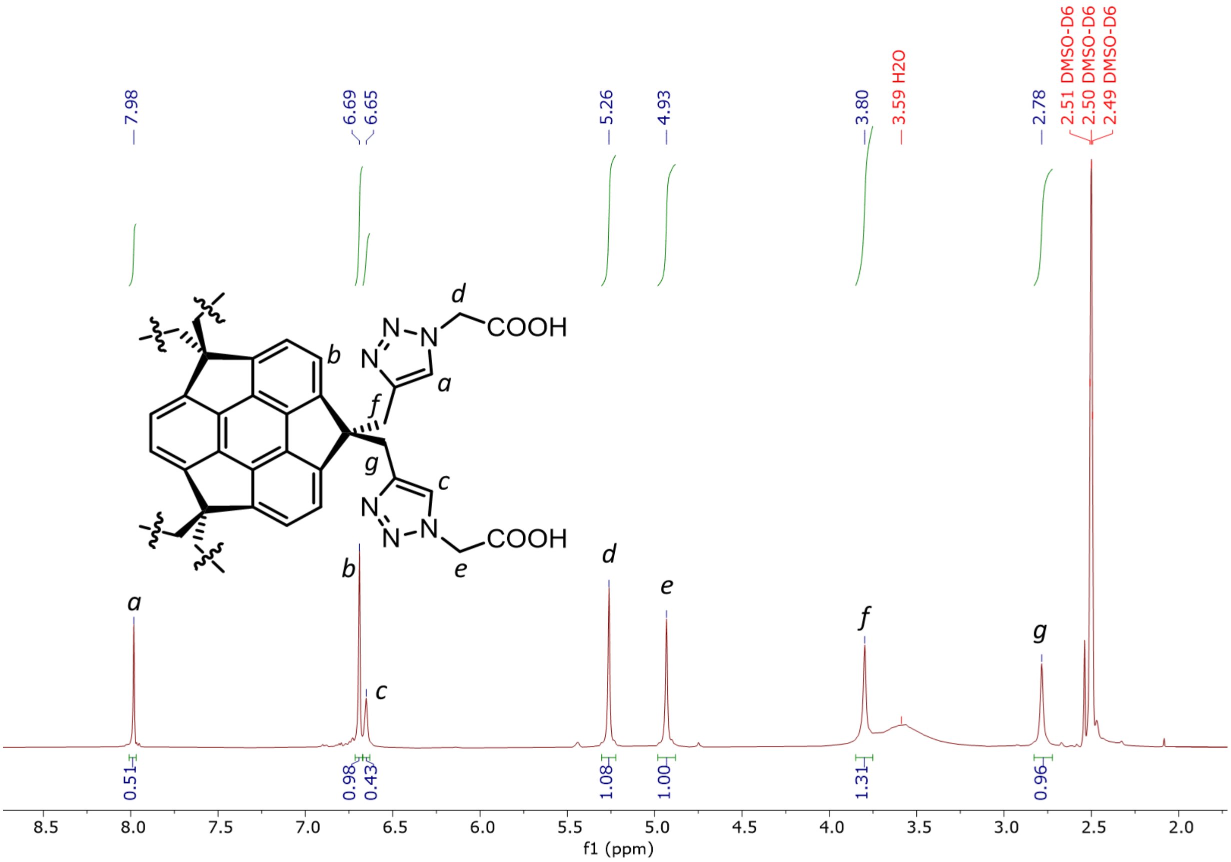

Figure S6. ^{1}H NMR spectrum of compound **2** (400 MHz, DMSO-d_6) with signals assigned.

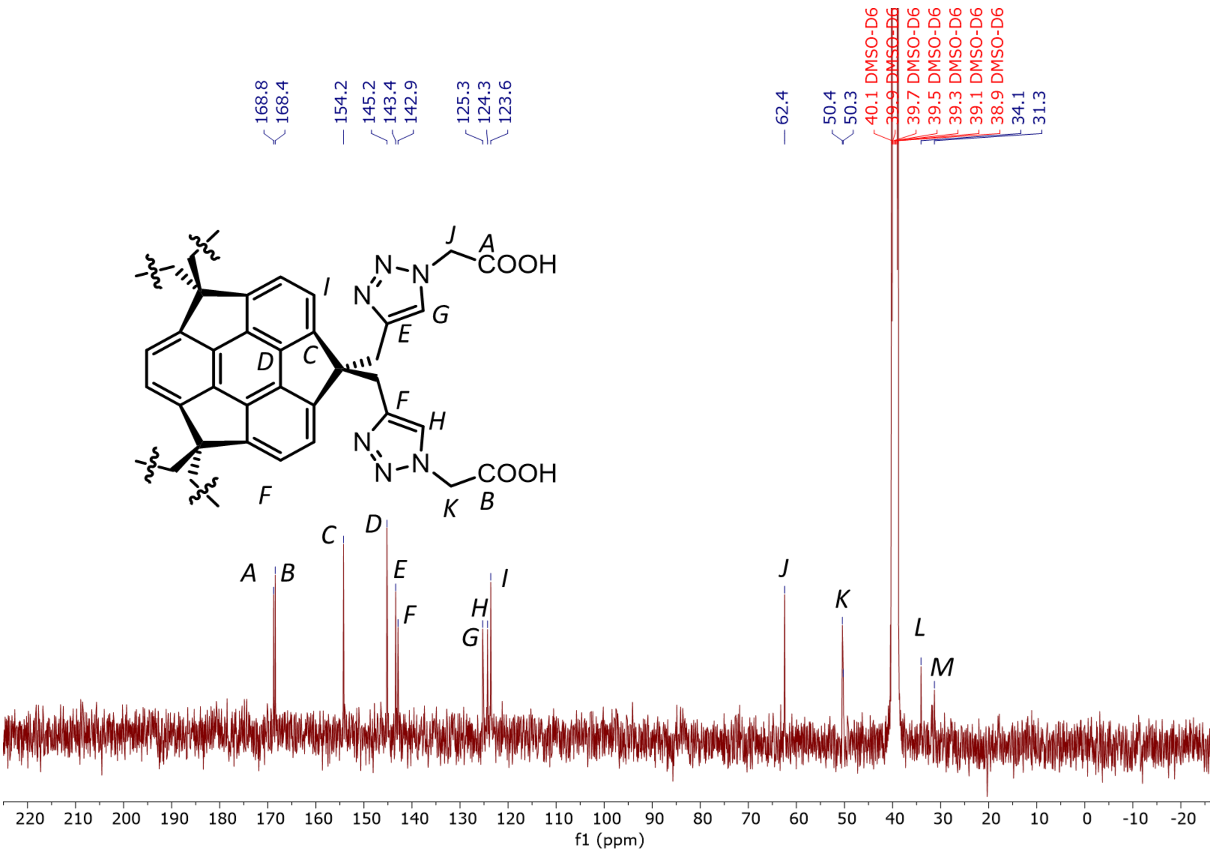

Figure S7. ^{13}C NMR spectrum of compound **2** (101 MHz, DMSO-d_6) with signals assigned.

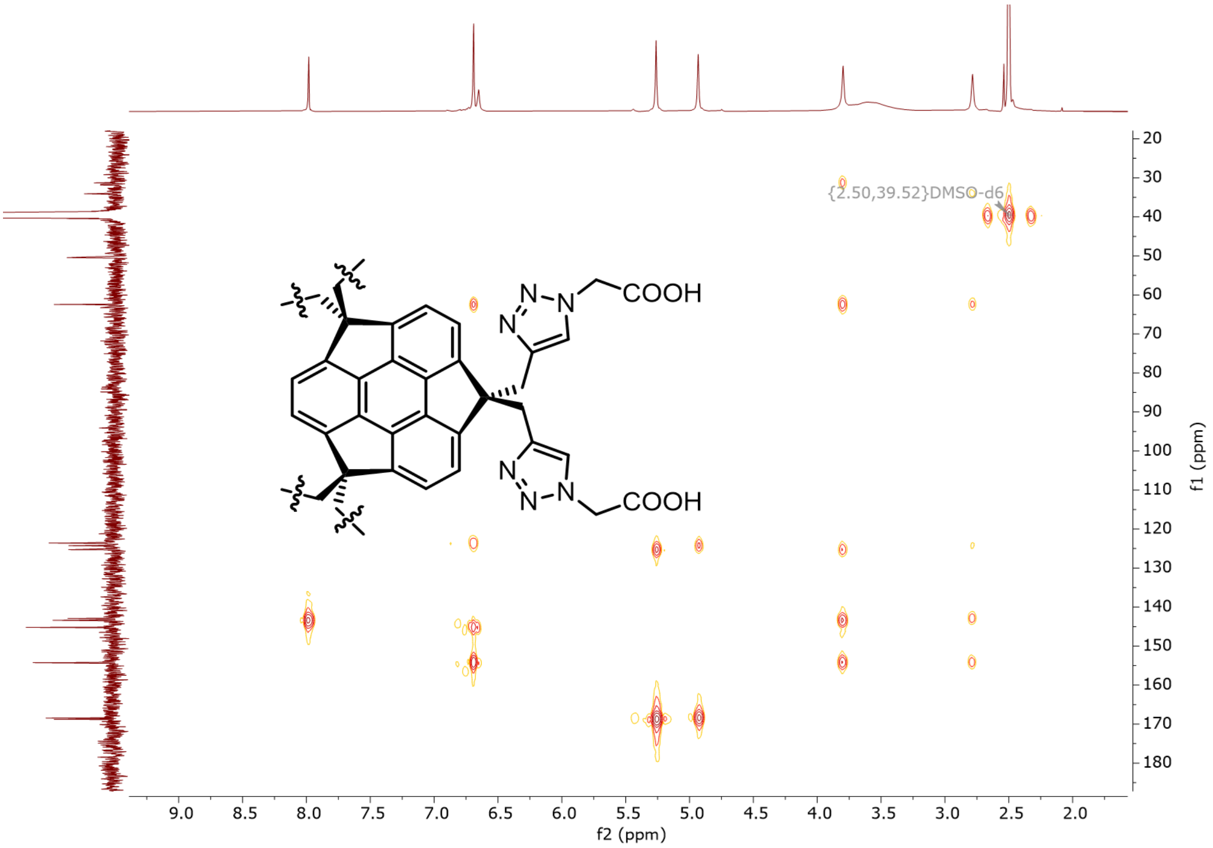

Figure S8. ^1H-^{13}C HMBC spectrum of compound **2** (DMSO-d_6).

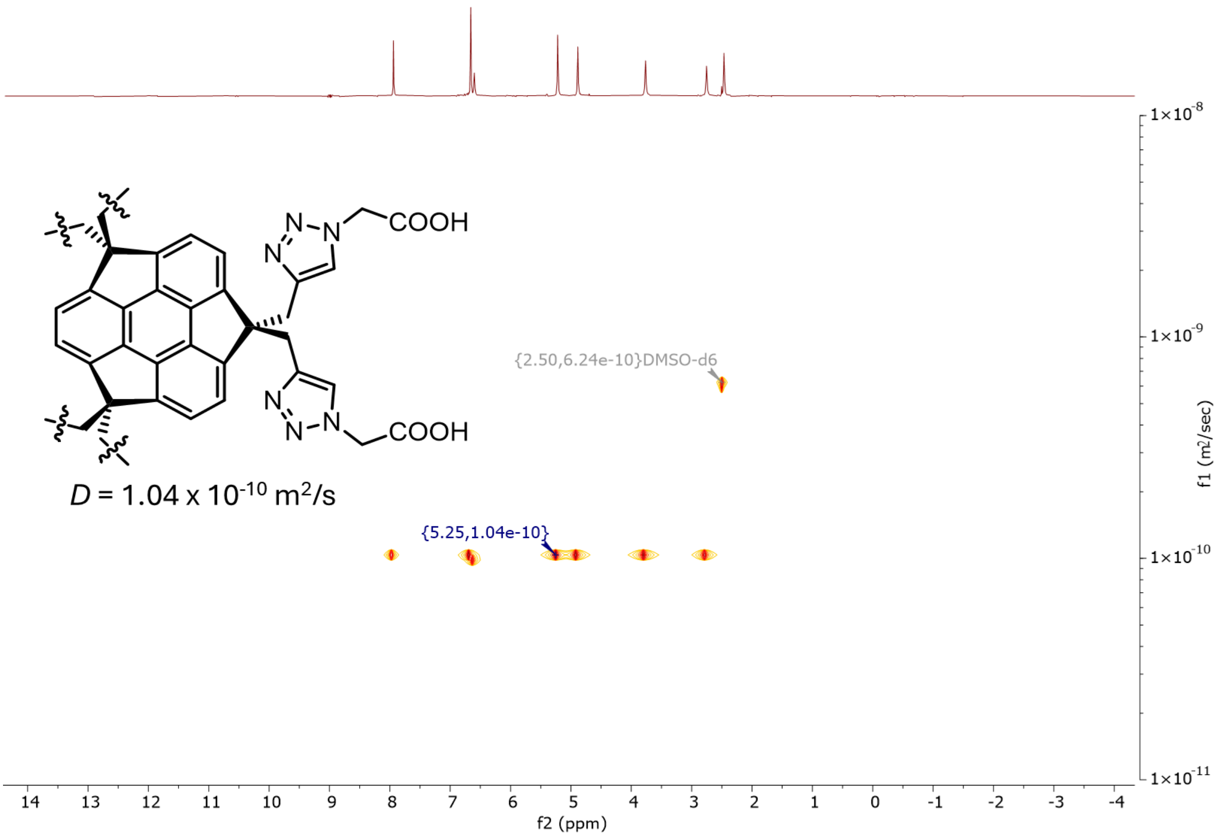

Figure S9. DOSY NMR spectrum of compound **2** (400 MHz, DMSO-d_6, 30 °C, 10 mM).

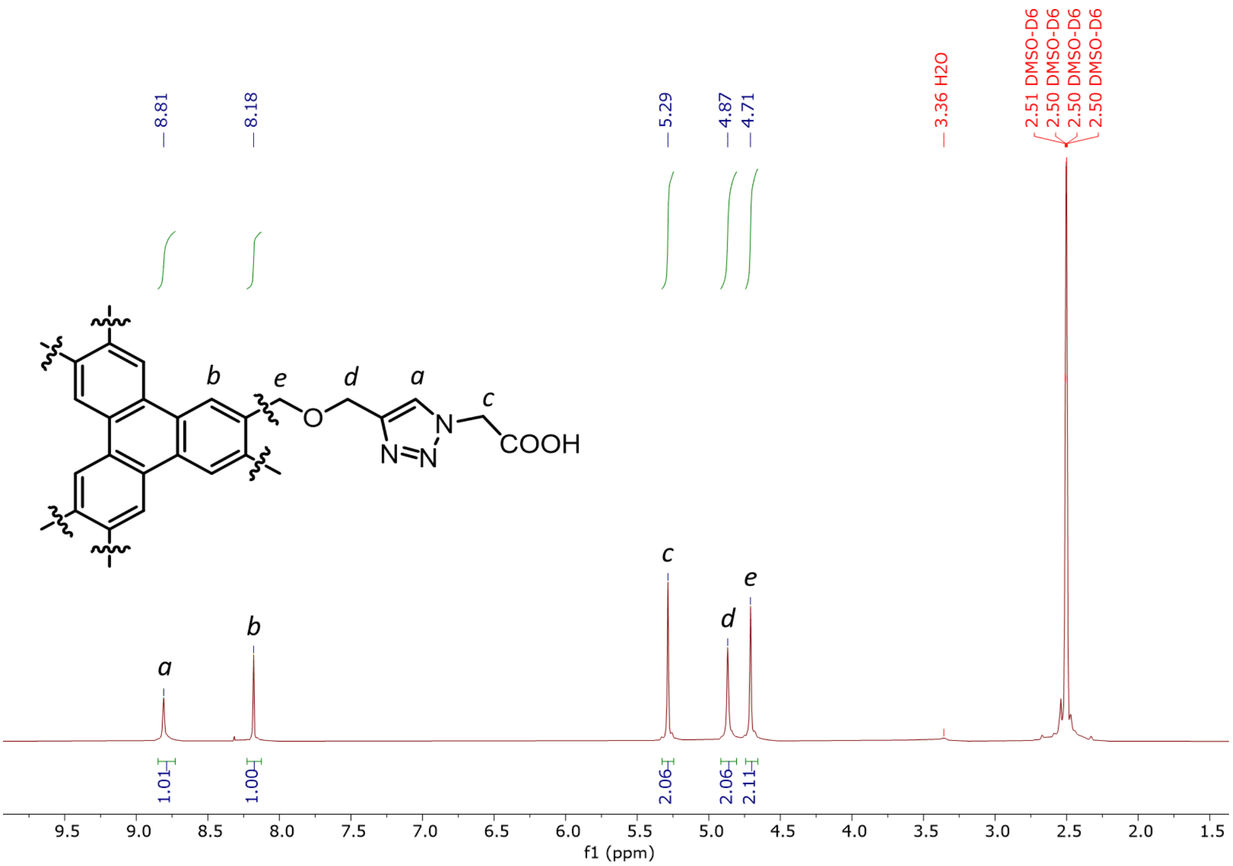

Figure S10. ^{1}H NMR spectrum of compound **3** (400 MHz, DMSO-d_6) with signals assigned.

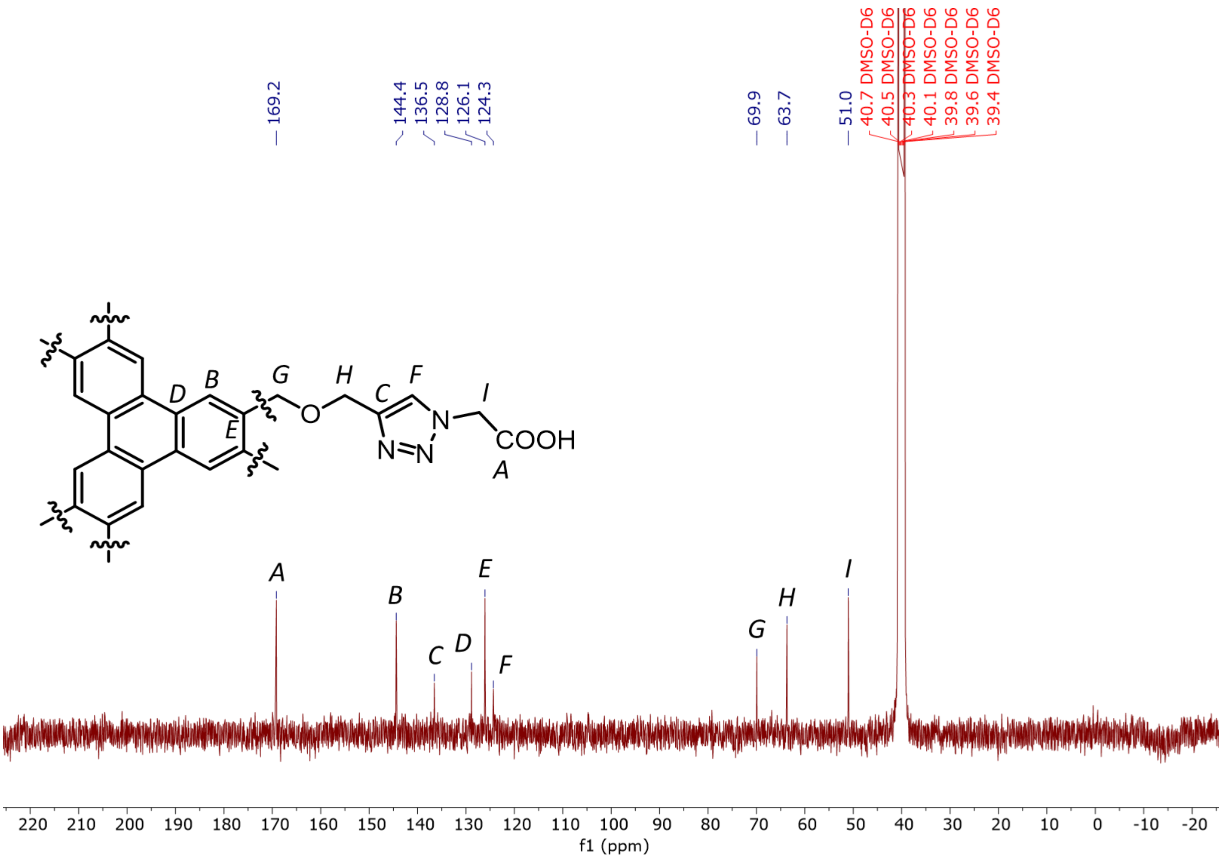

Figure S11. ^{13}C NMR spectrum of compound **3** (101 MHz, DMSO-d_6) with signals assigned.

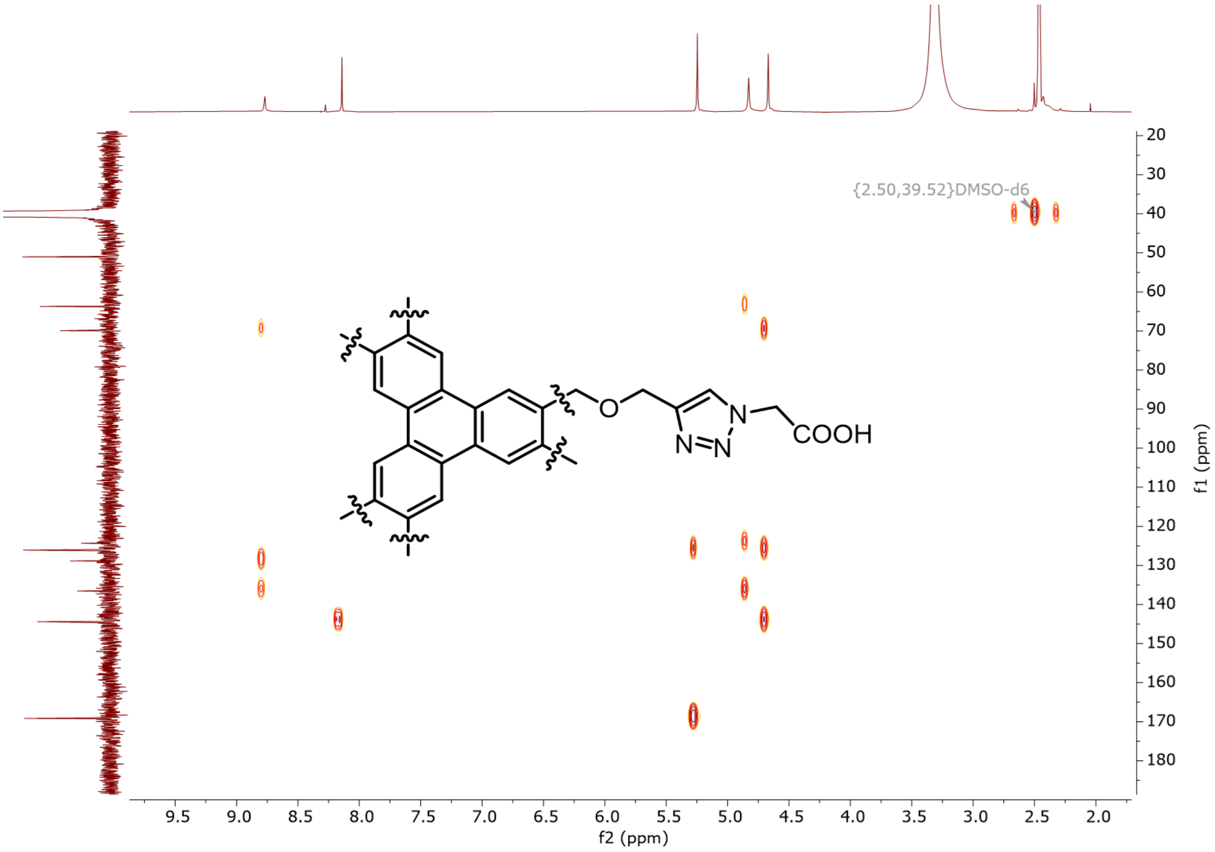

Figure S12. ^1H-^{13}C HMBC spectrum of compound **3** (DMSO-d_6).

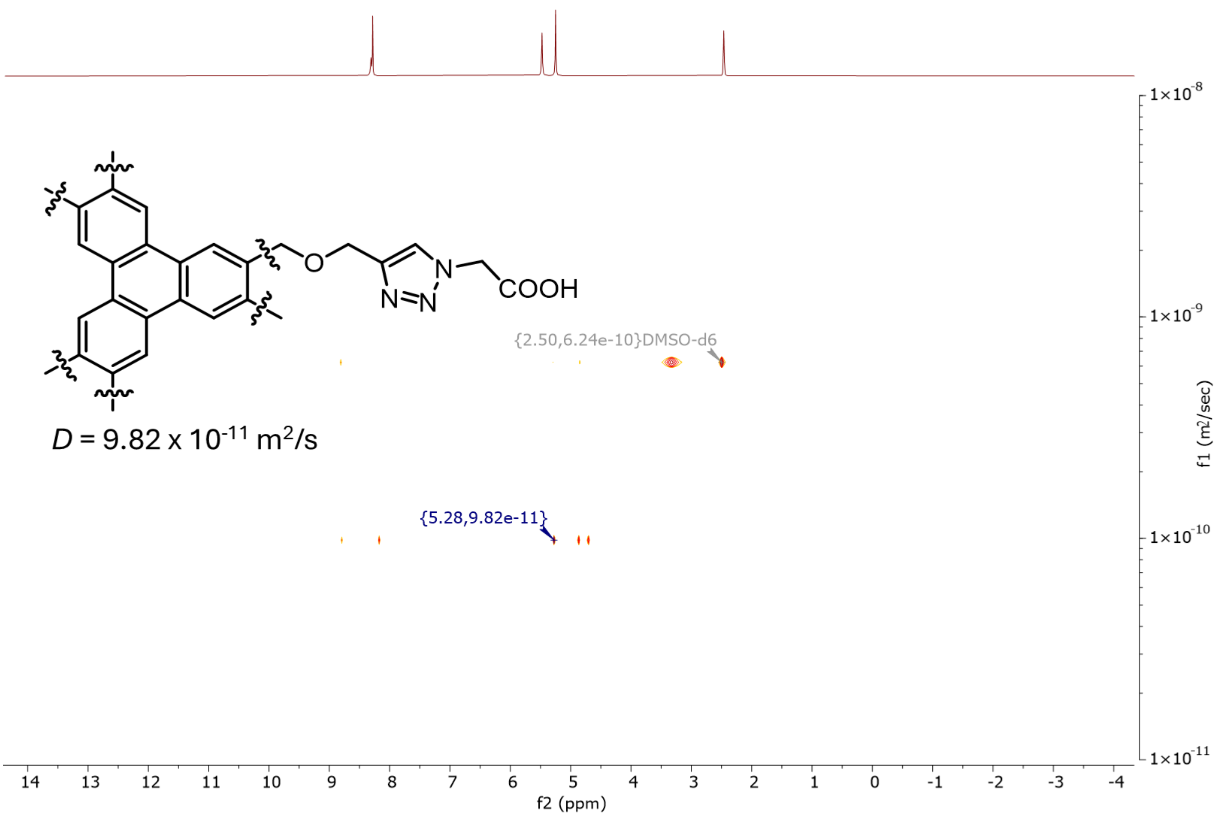

Figure S13. DOSY NMR spectrum of compound **3** (400 MHz, DMSO-*d*6, 30 °C, 10 mM).

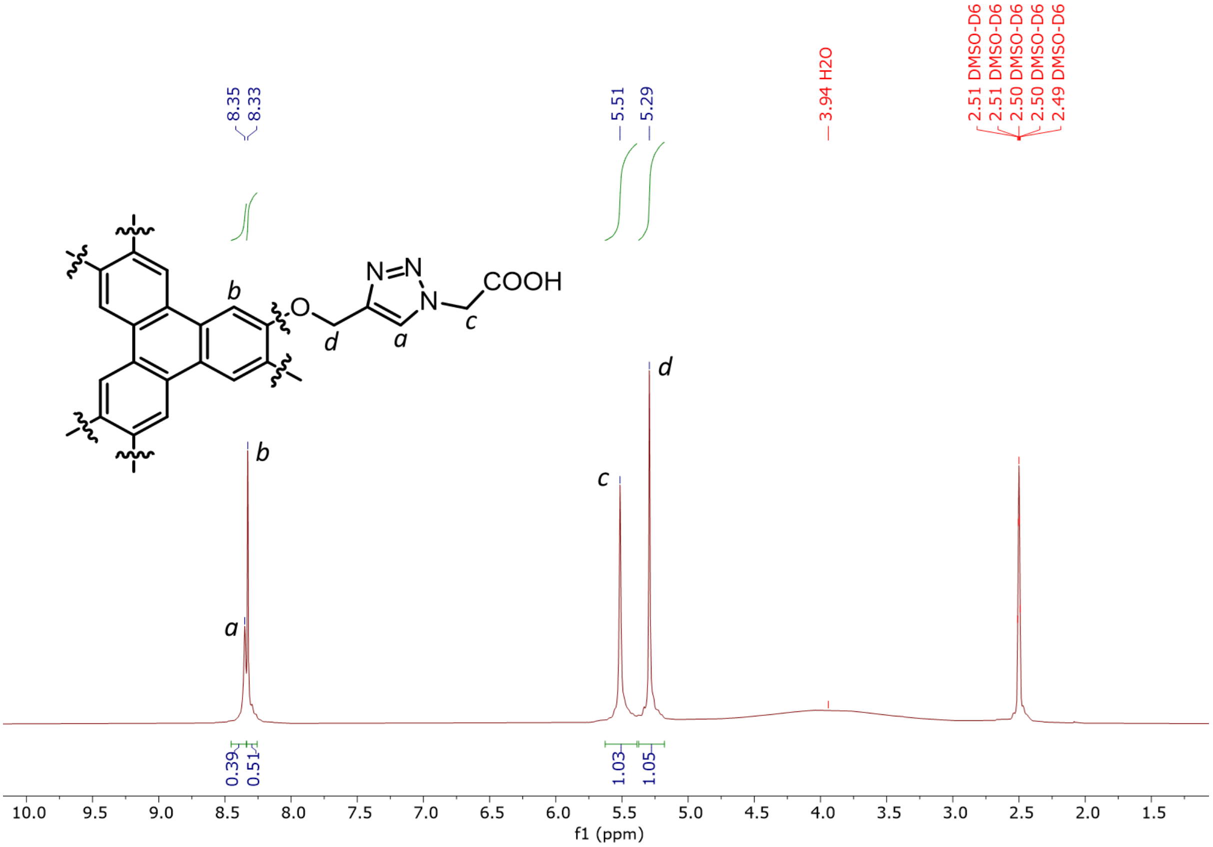

Figure S14. ^{1}H NMR spectrum of compound **4** (400 MHz, DMSO-*d*6) with signals assigned.

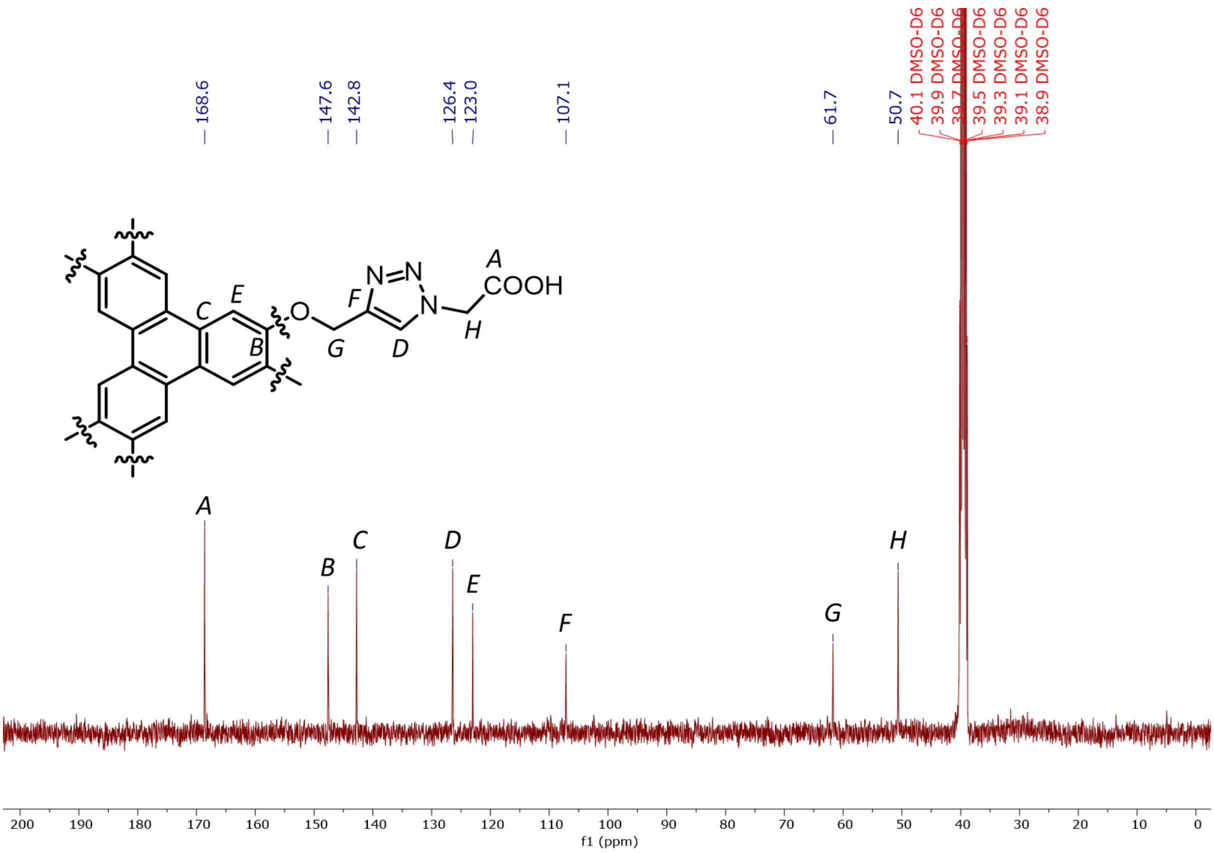

Figure S15. ^{13}C NMR spectrum of compound **4** (101 MHz, DMSO-d_6) with signals assigned.

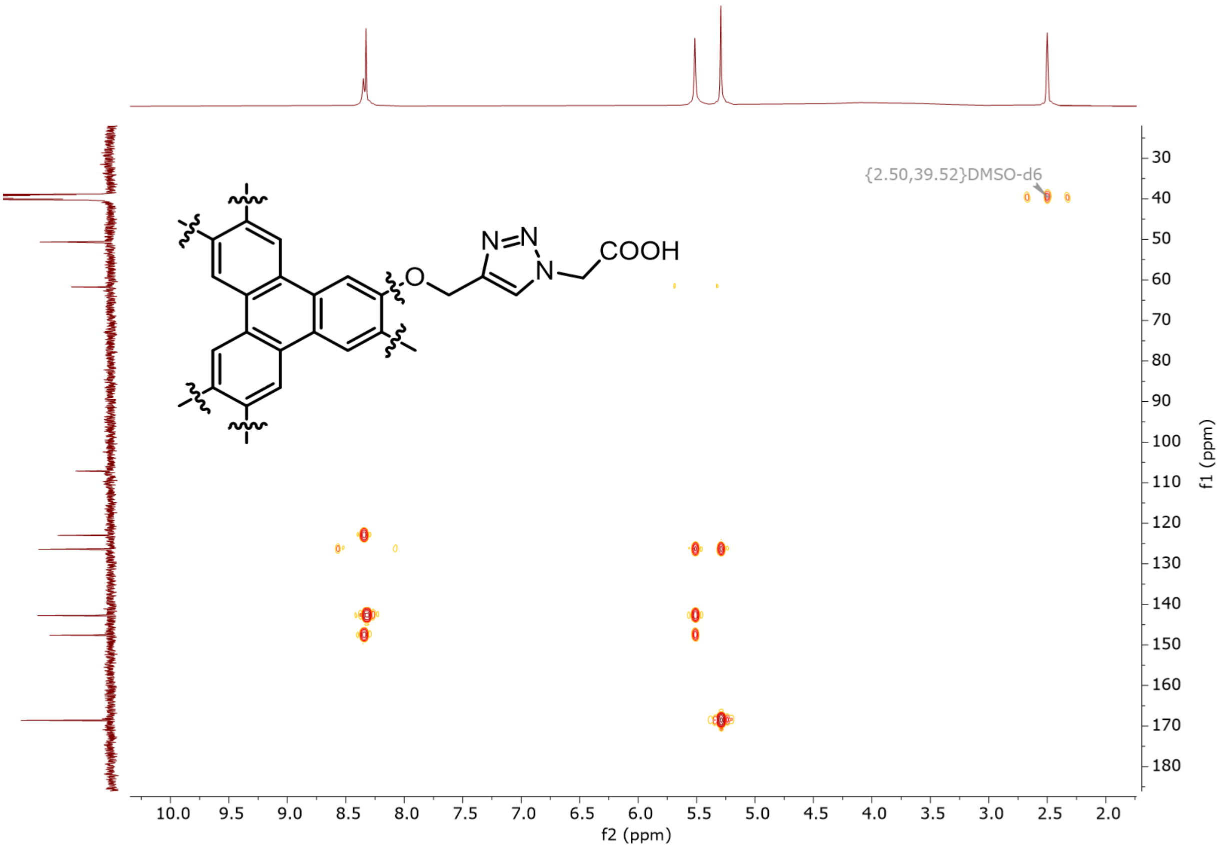

Figure S16. ^{1}H-^{13}C HMBC spectrum of compound **4** (DMSO-d_6).

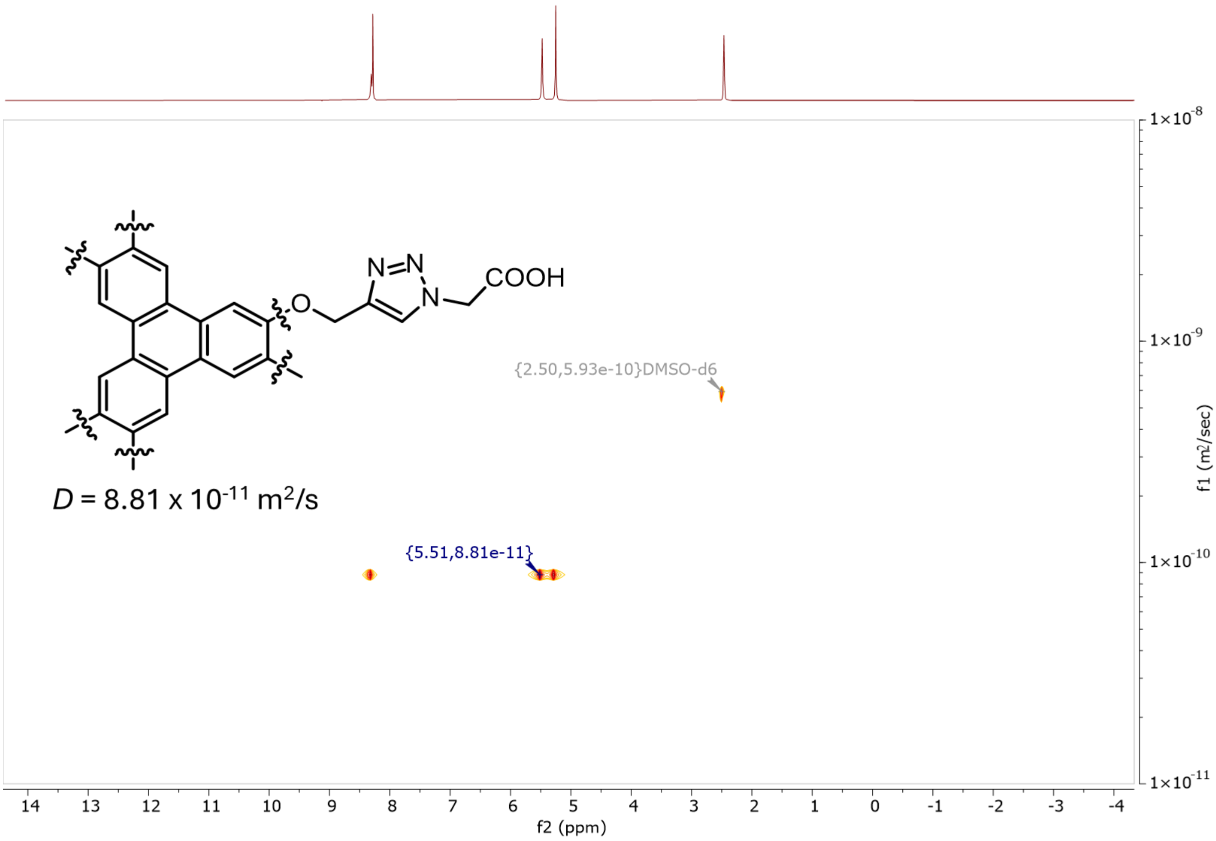

Figure S17. DOSY NMR spectrum of compound **4** (400 MHz, DMSO-d_6, 30 °C, 10 mM).

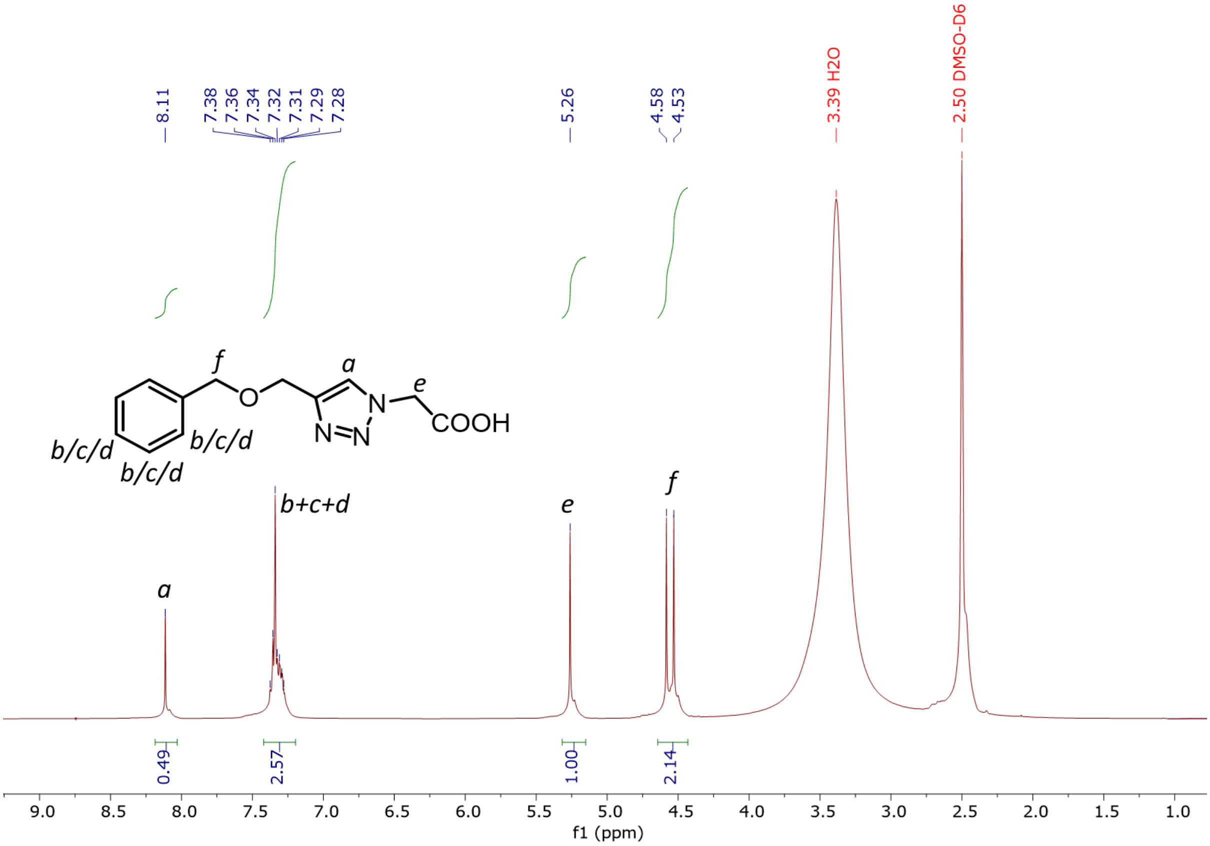

Figure S18. ^{1}H NMR spectrum of compound **5** (400 MHz, DMSO-d_6) with signals assigned.

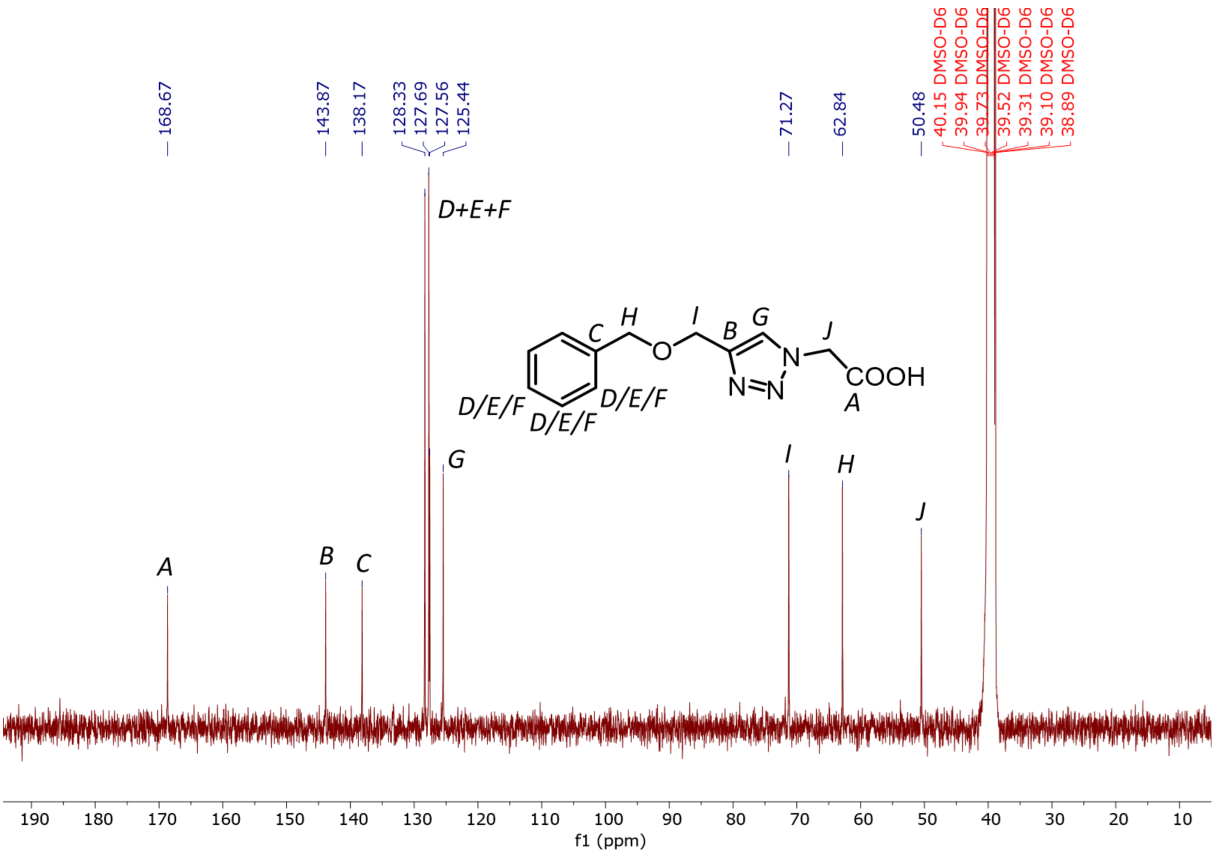

Figure S19. ^{13}C NMR spectrum of compound **5** (101 MHz, DMSO-d_6) with signals assigned.

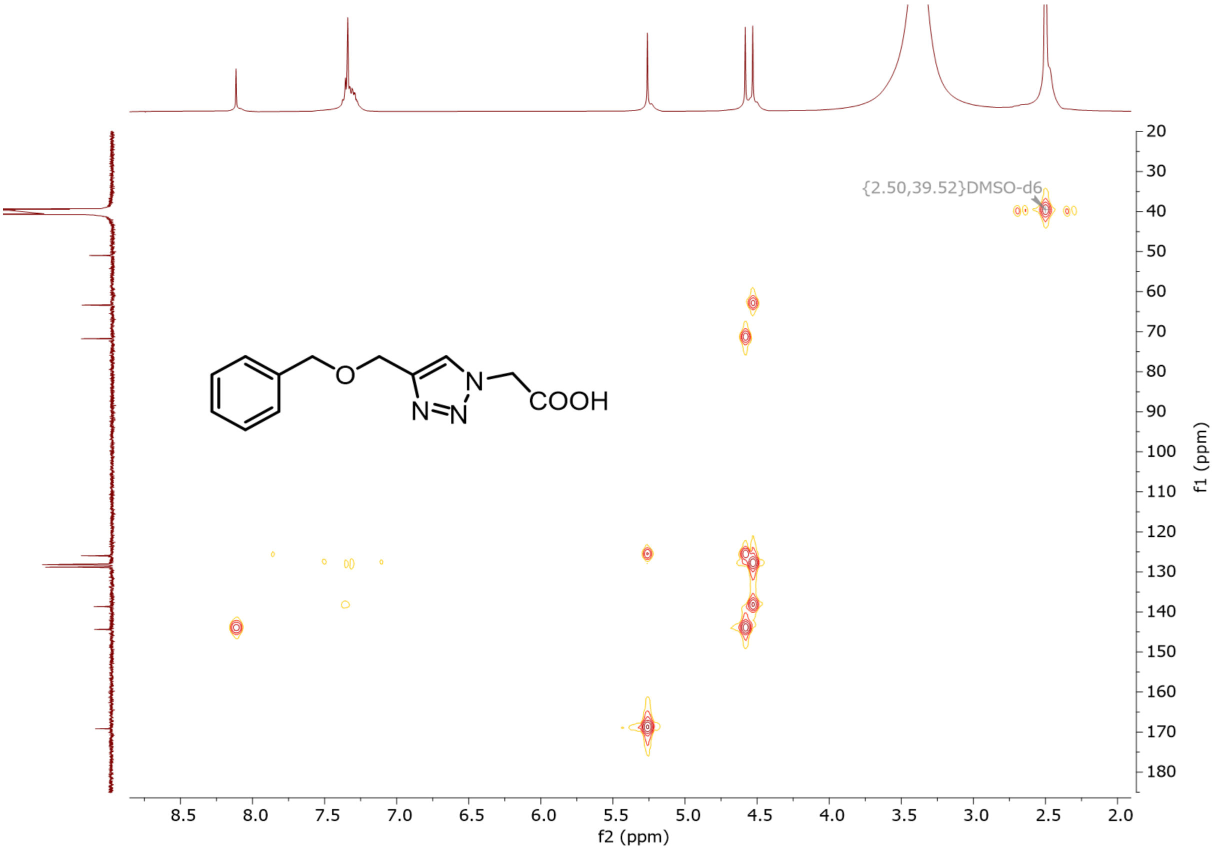

Figure S20. ^{1}H-^{13}C HMBC spectrum of compound **5** (DMSO-d_6).

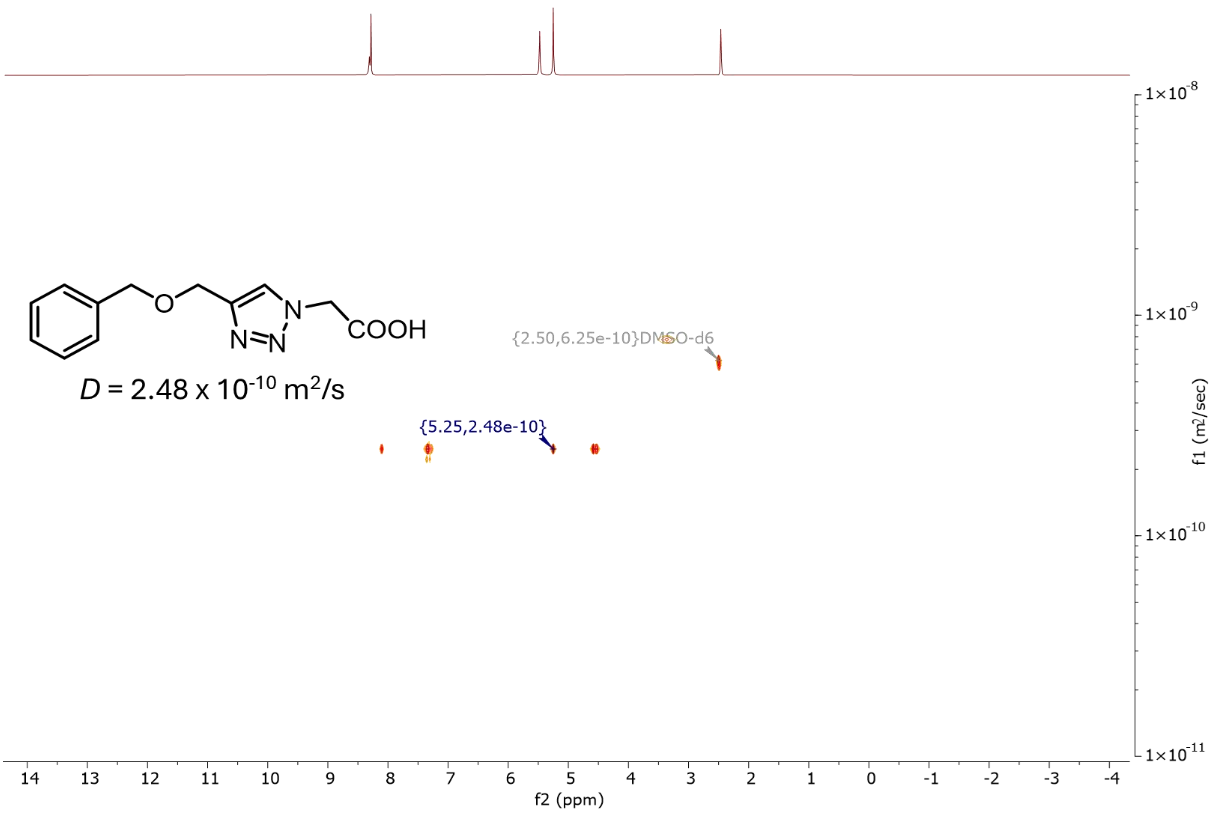

Figure S21. DOSY NMR spectrum of compound **5** (400 MHz, DMSO-d_6, 30 °C, 10 mM).

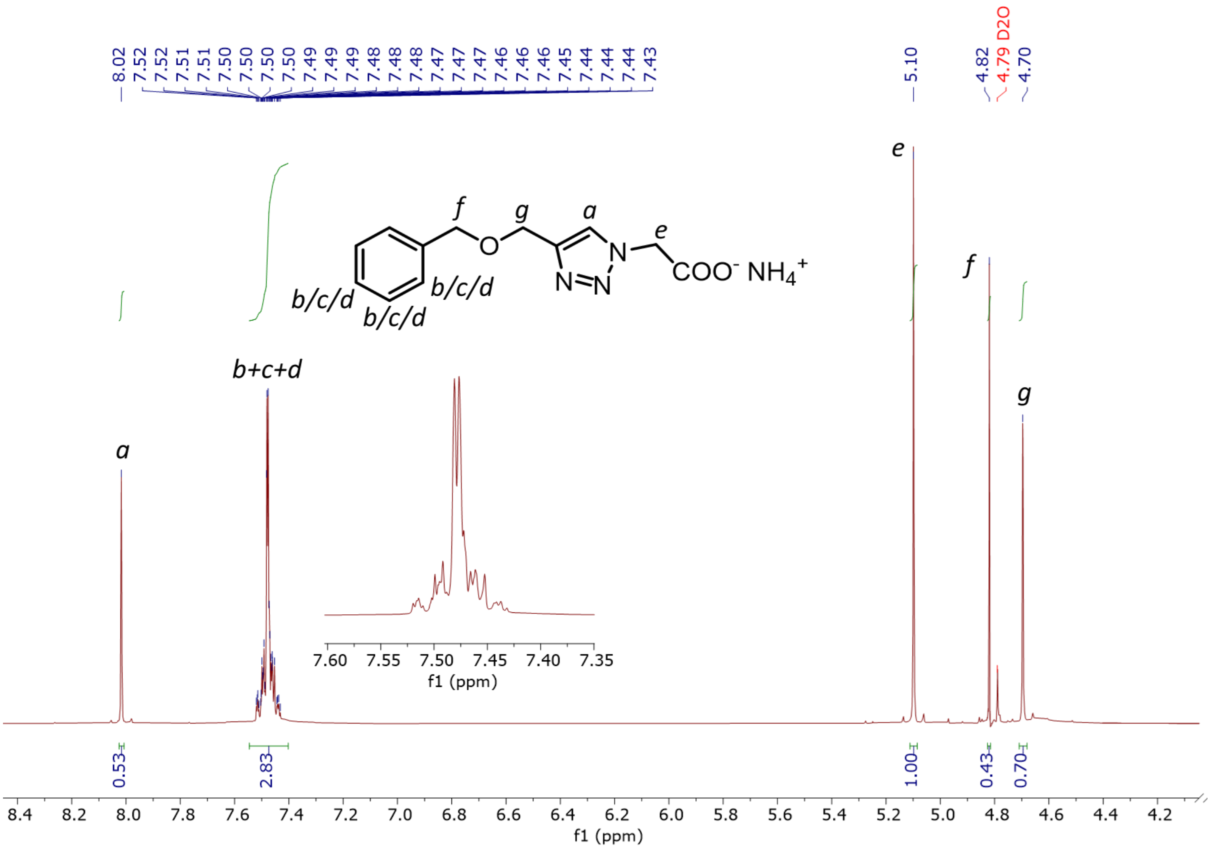

Figure S22. ^{1}H NMR spectrum of compound **5b** (400 MHz, D_2O) with signals assigned.

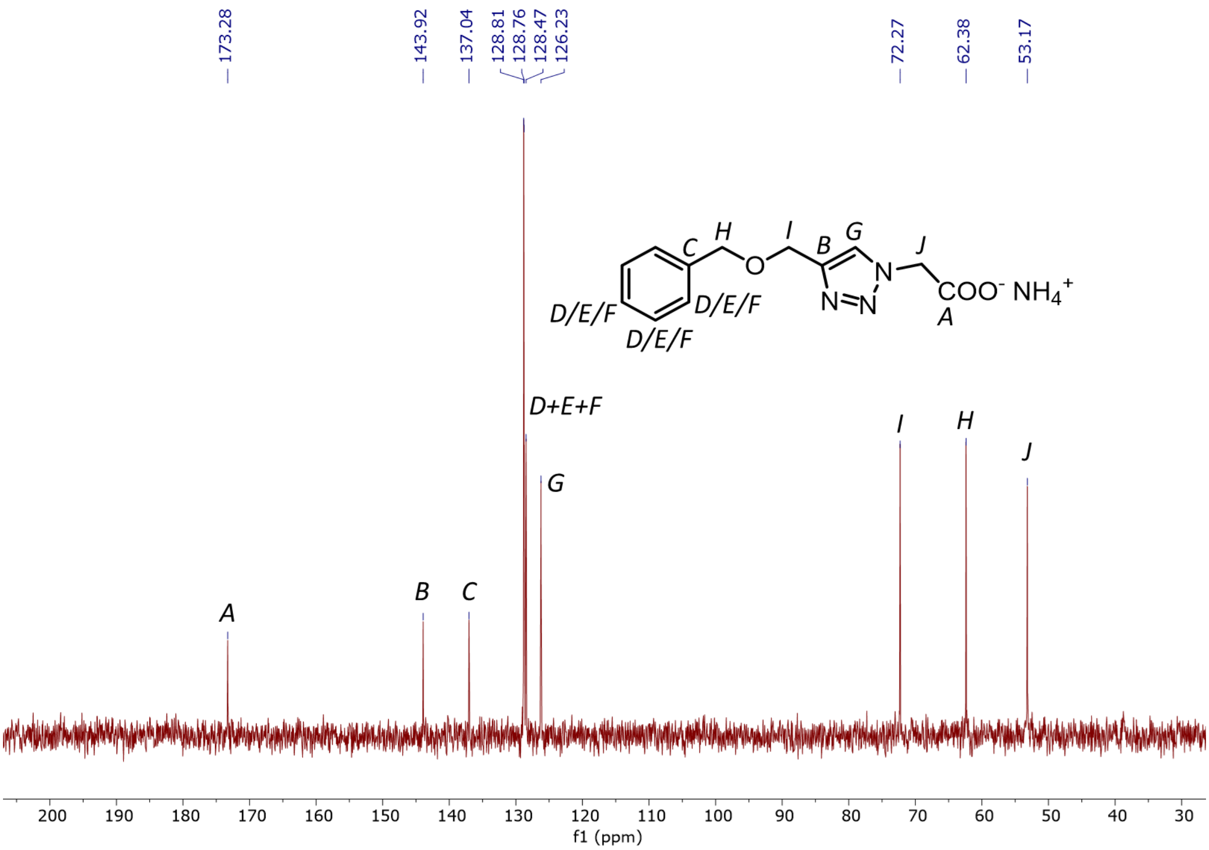

Figure S23. ^{13}C NMR spectrum of compound **5b** (101 MHz, D_2O) with signals assigned.

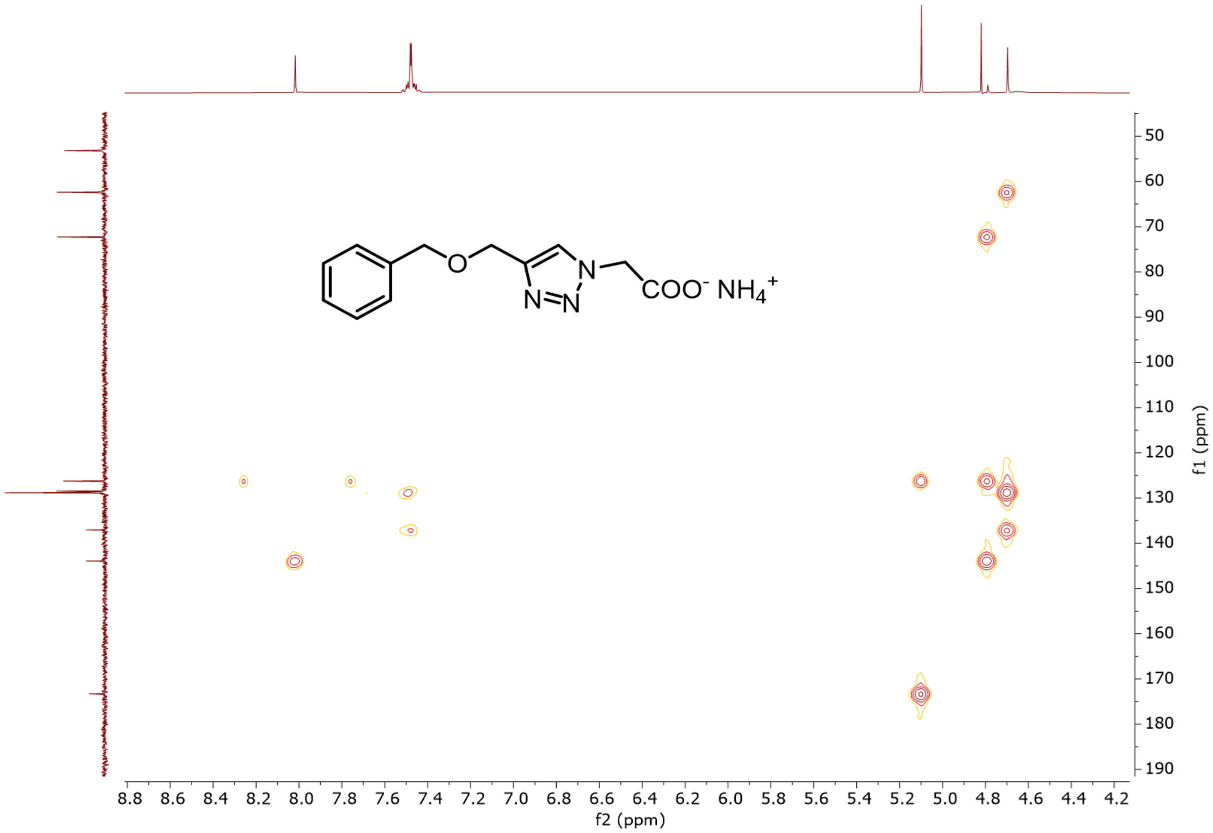

Figure S24. ^{1}H-^{13}C NMR spectrum of compound **5b** (D_2O).

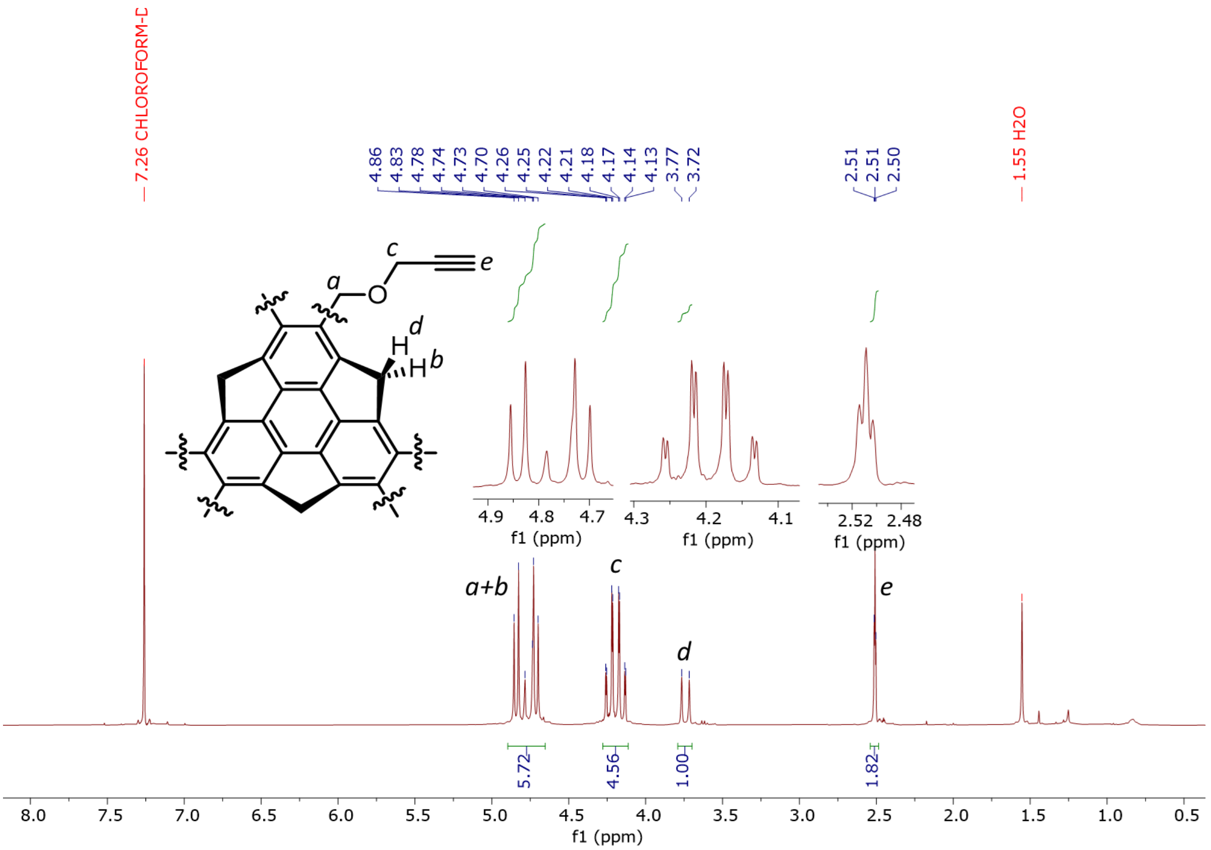

Figure S25. ^{1}H NMR spectrum of compound **7** (400 MHz, $CDCl_3$) with signals assigned.

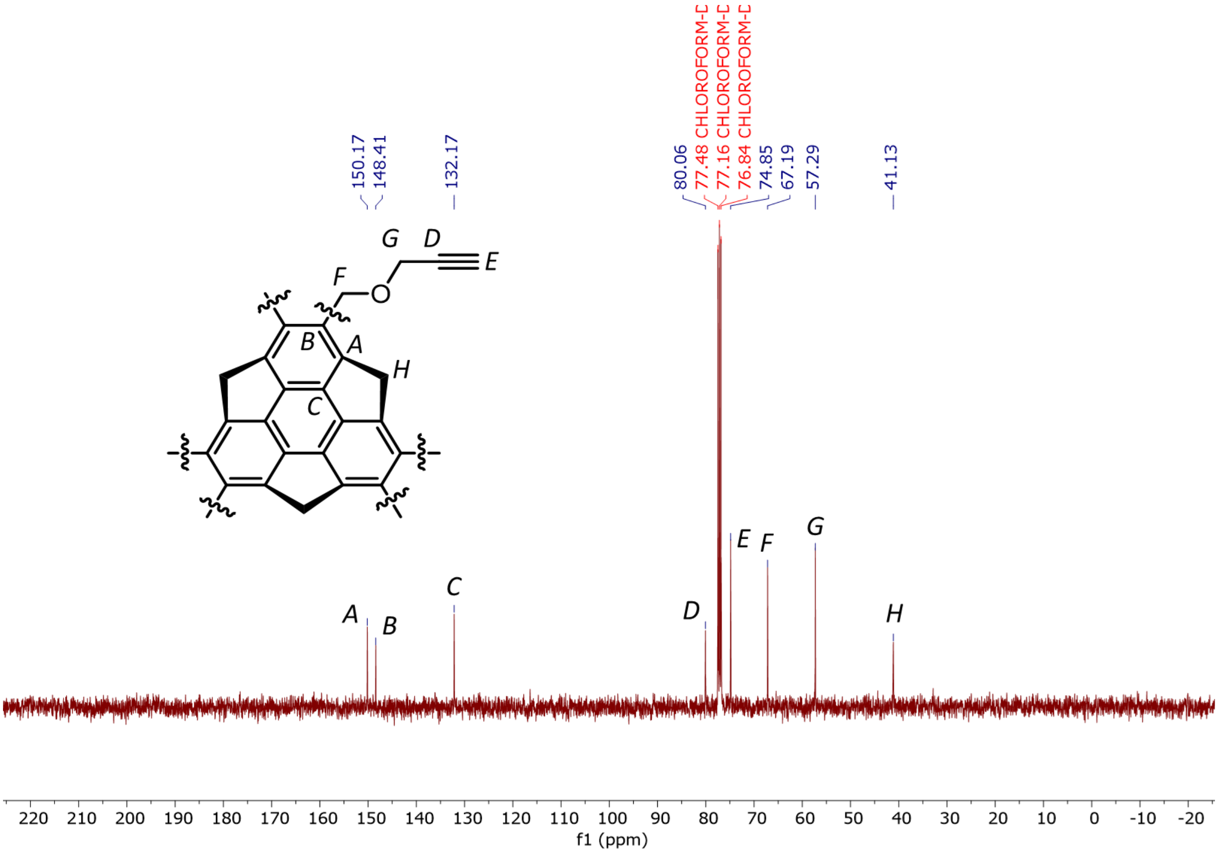

Figure S26. ^{13}C NMR spectrum of compound **7** (101 MHz, $CDCl_3$) with signals assigned.

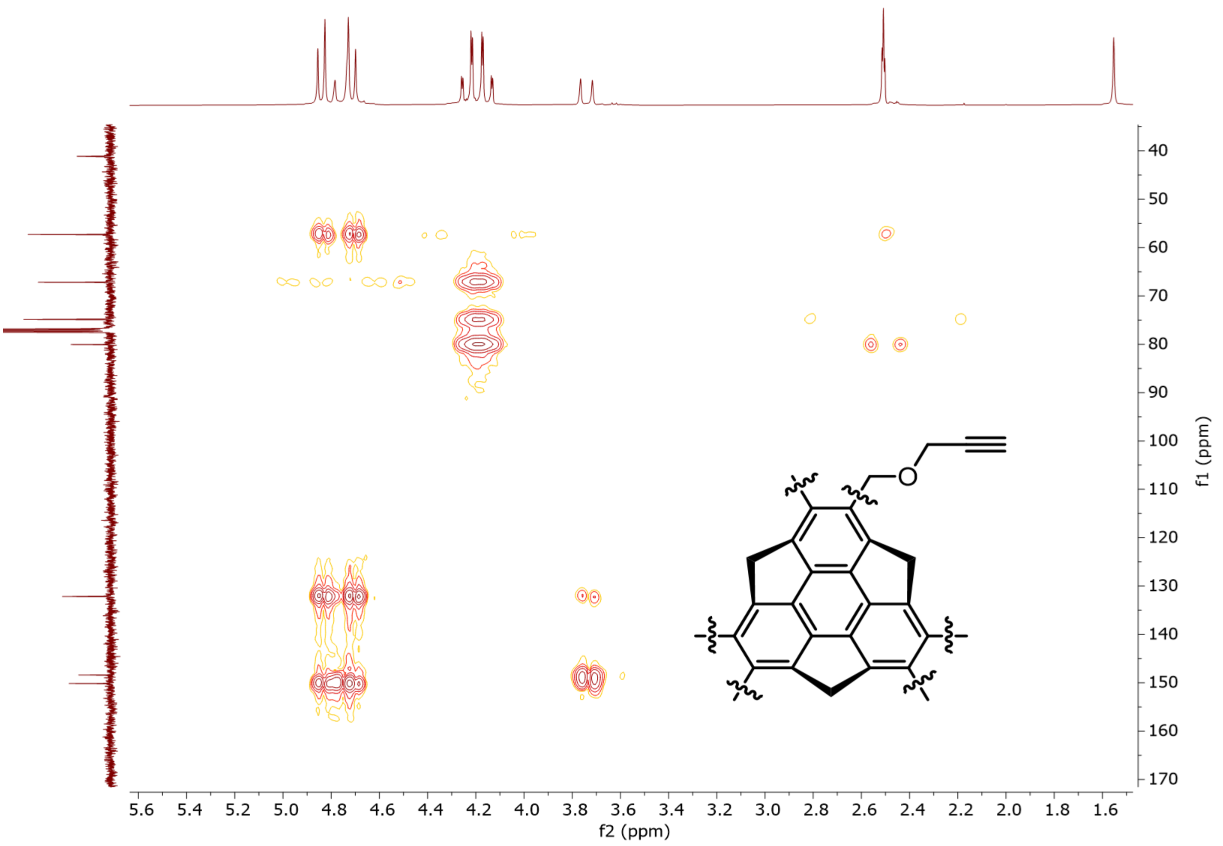

Figure S27. ^{1}H-^{13}C HMBC spectrum of compound **7** ($CDCl_3$).

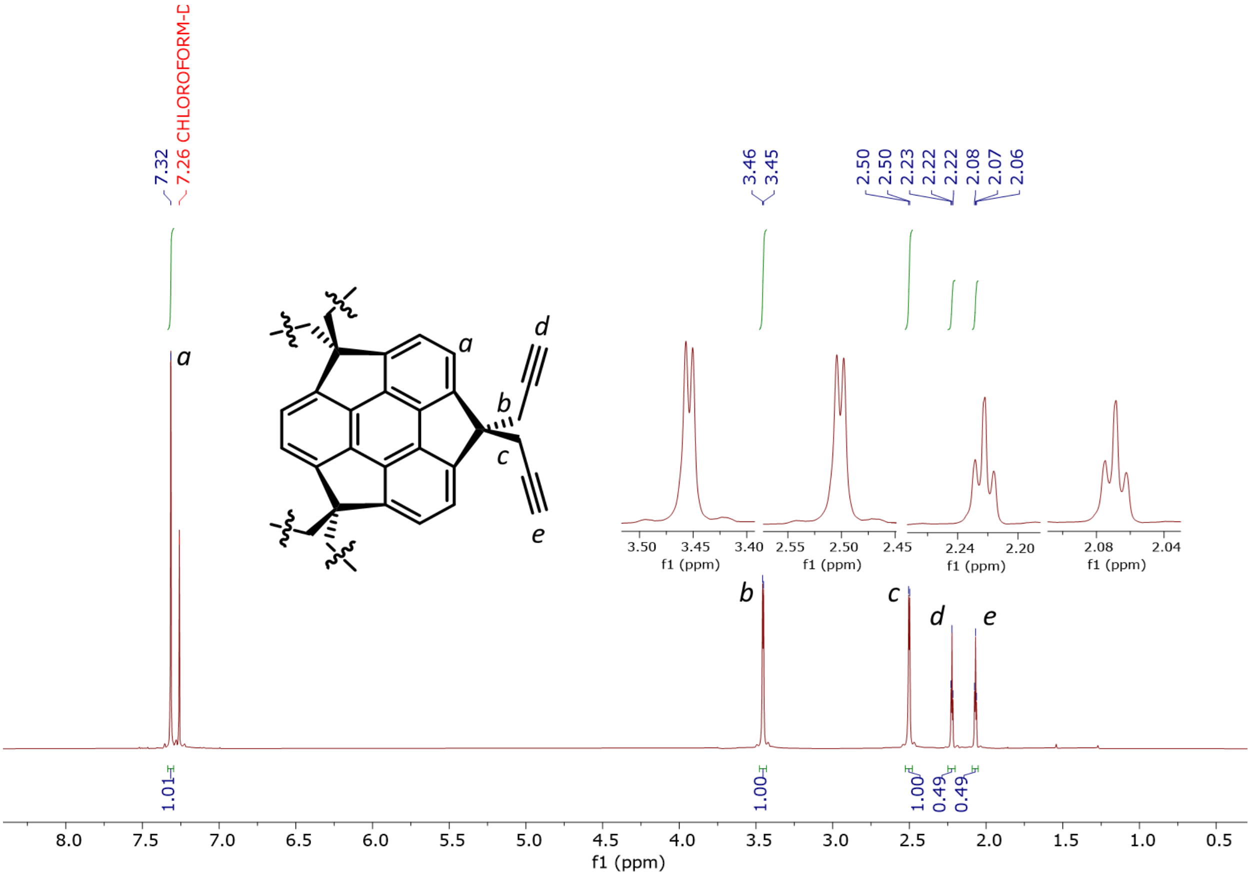

Figure S28. ^{1}H NMR spectrum of compound **9** (400 MHz, $CDCl_3$) with signals assigned.

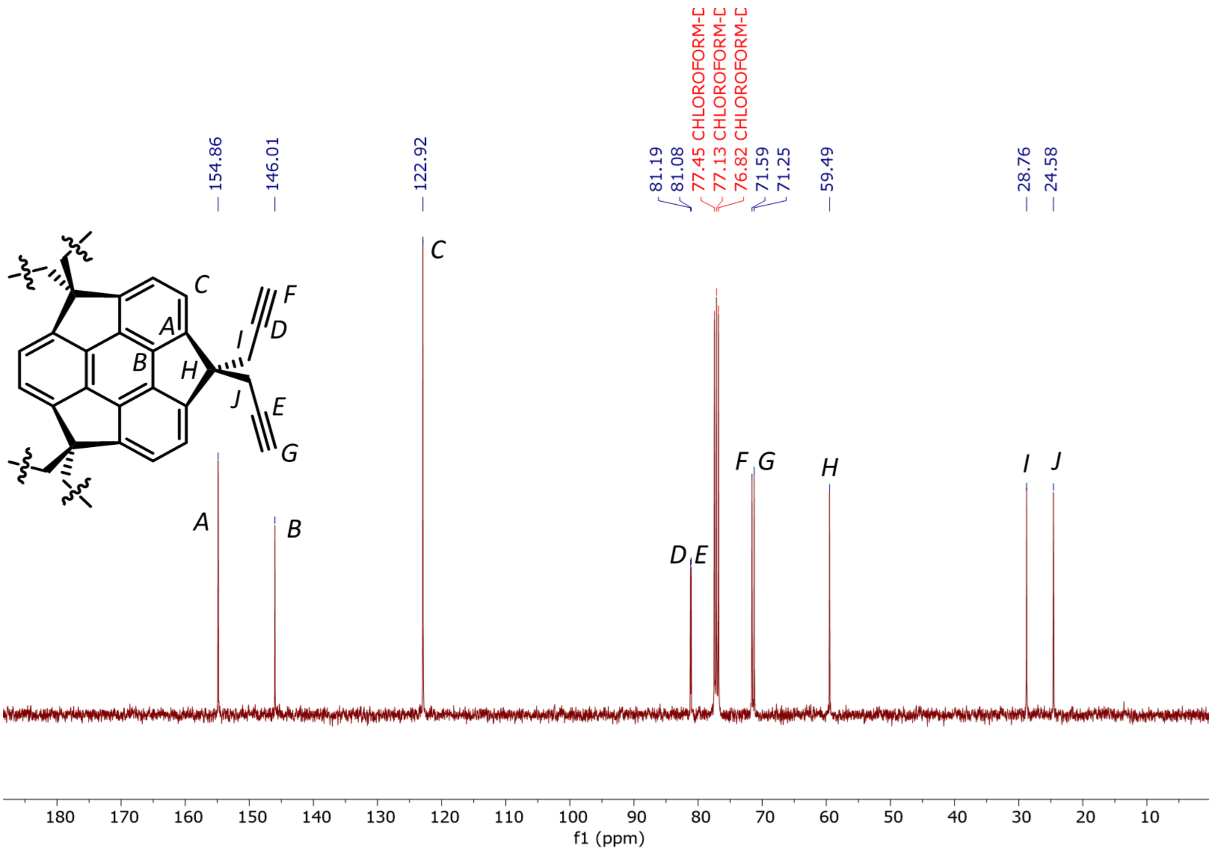

Figure S29. ^{13}C NMR spectrum of compound **9** (101 MHz, $CDCl_3$) with signals assigned.

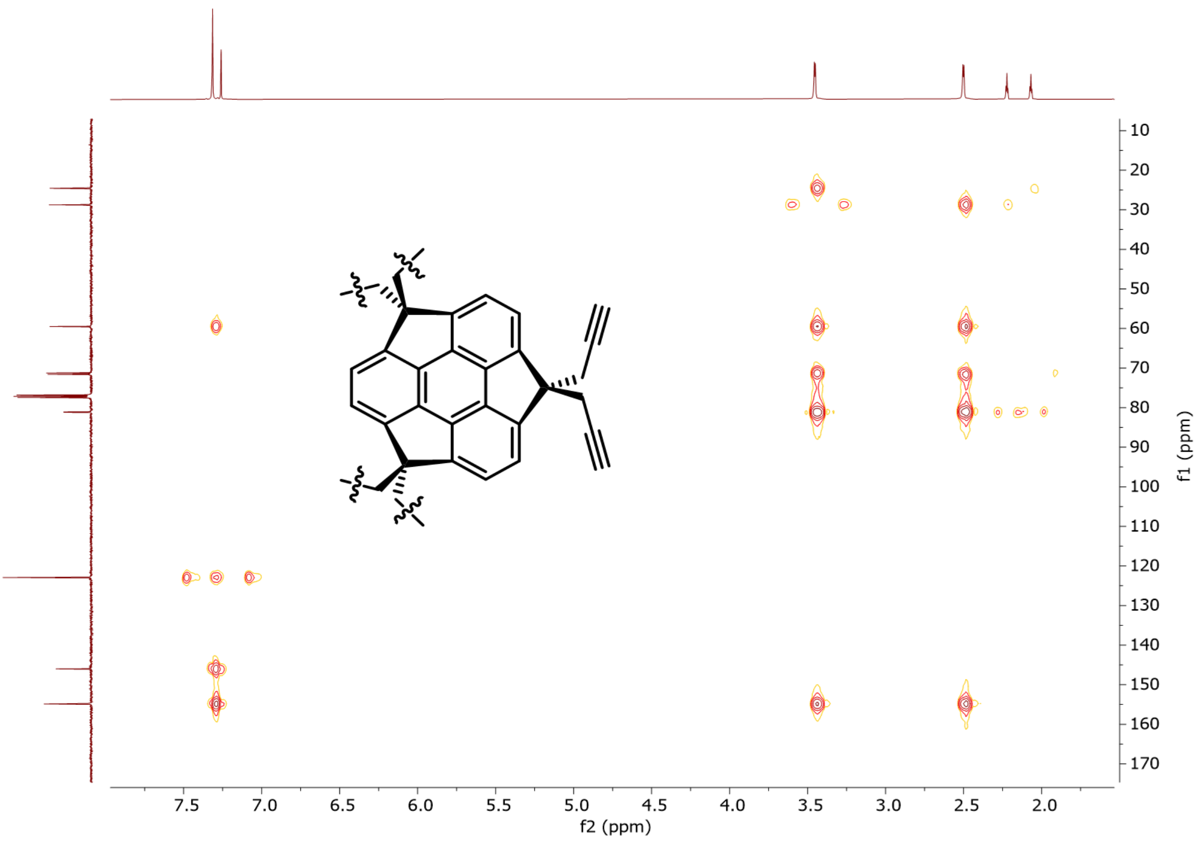

Figure S30. ^{1}H-^{13}C HMBC spectrum of compound **9** ($CDCl_3$).

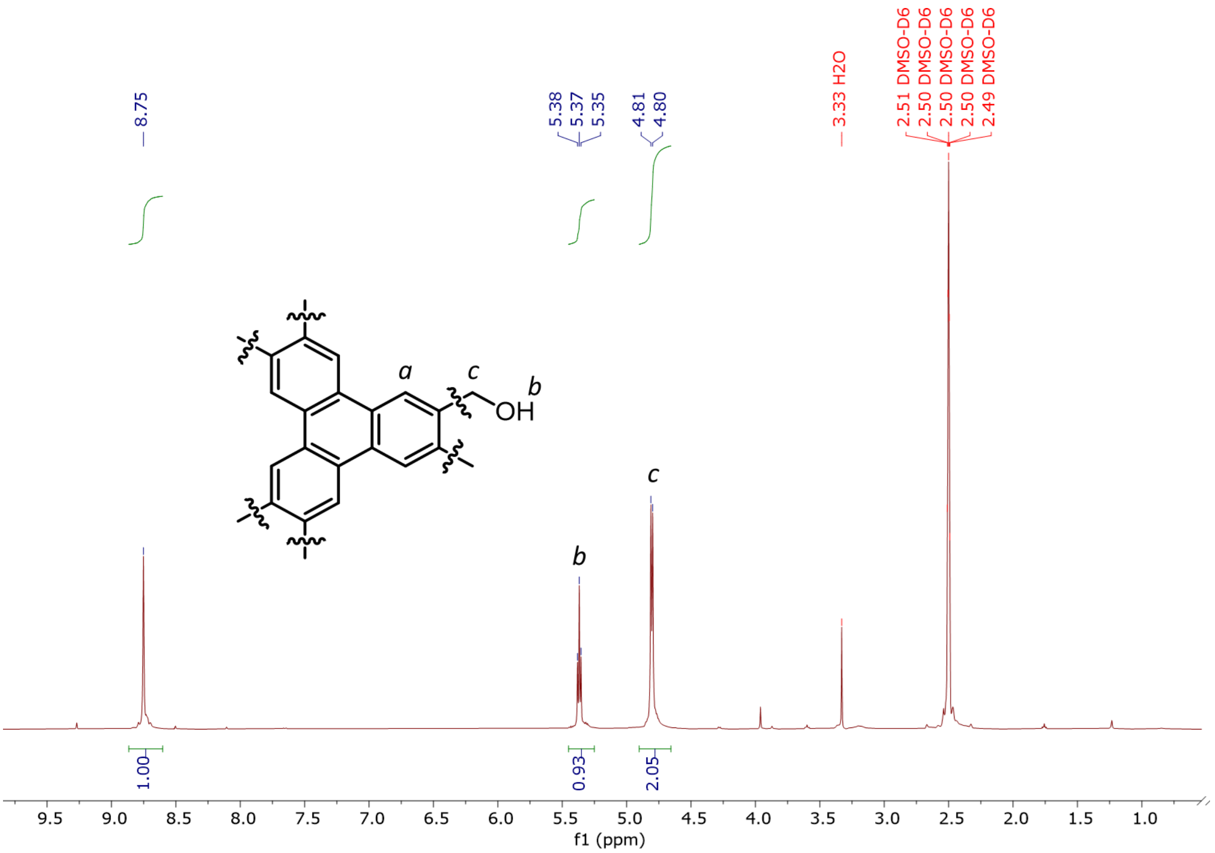

Figure S31. ^{1}H NMR spectrum of compound **11** (400 MHz, DMSO-d_6) with signals assigned.

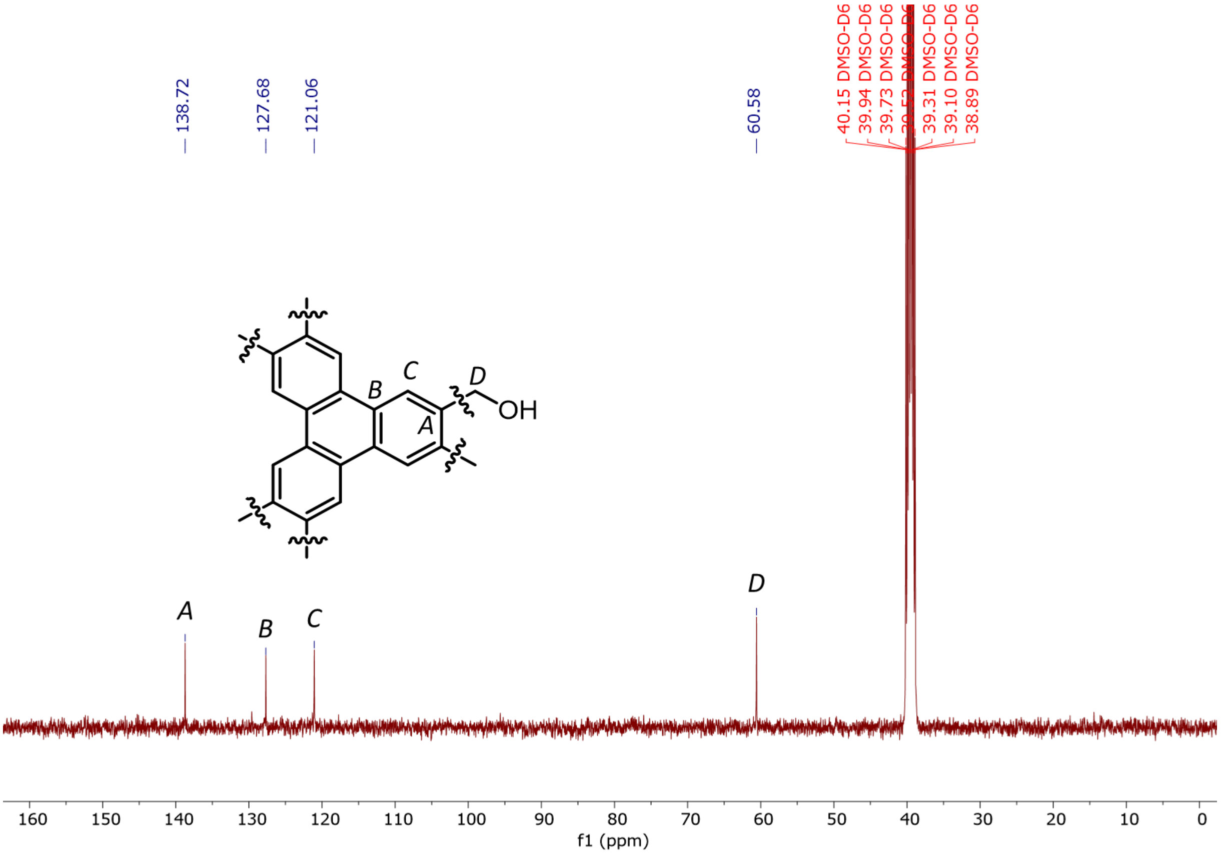

Figure S32. ^{13}C NMR spectrum of compound **11** (101 MHz, DMSO-d_6) with signals assigned.

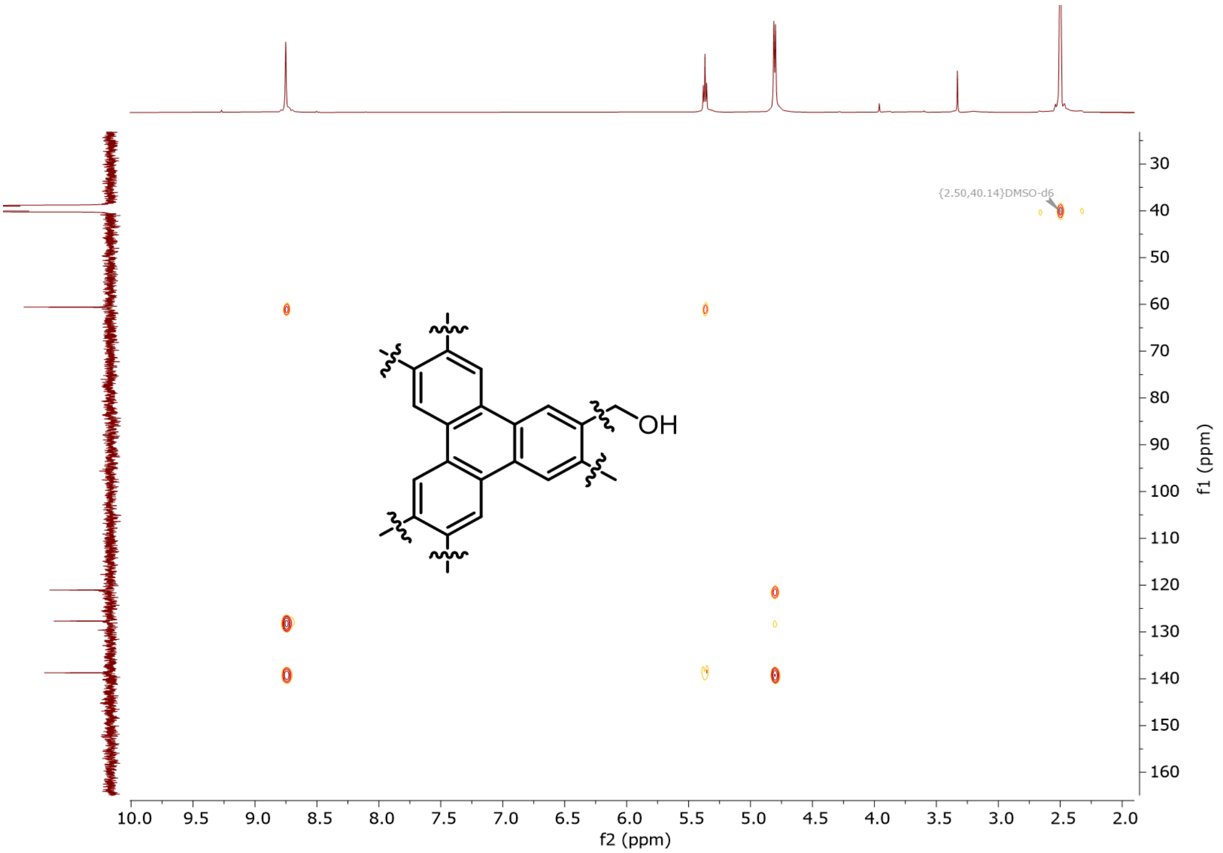

Figure S33. ^{1}H-^{13}C HMBC spectrum of compound **11** (DMSO-d_6).

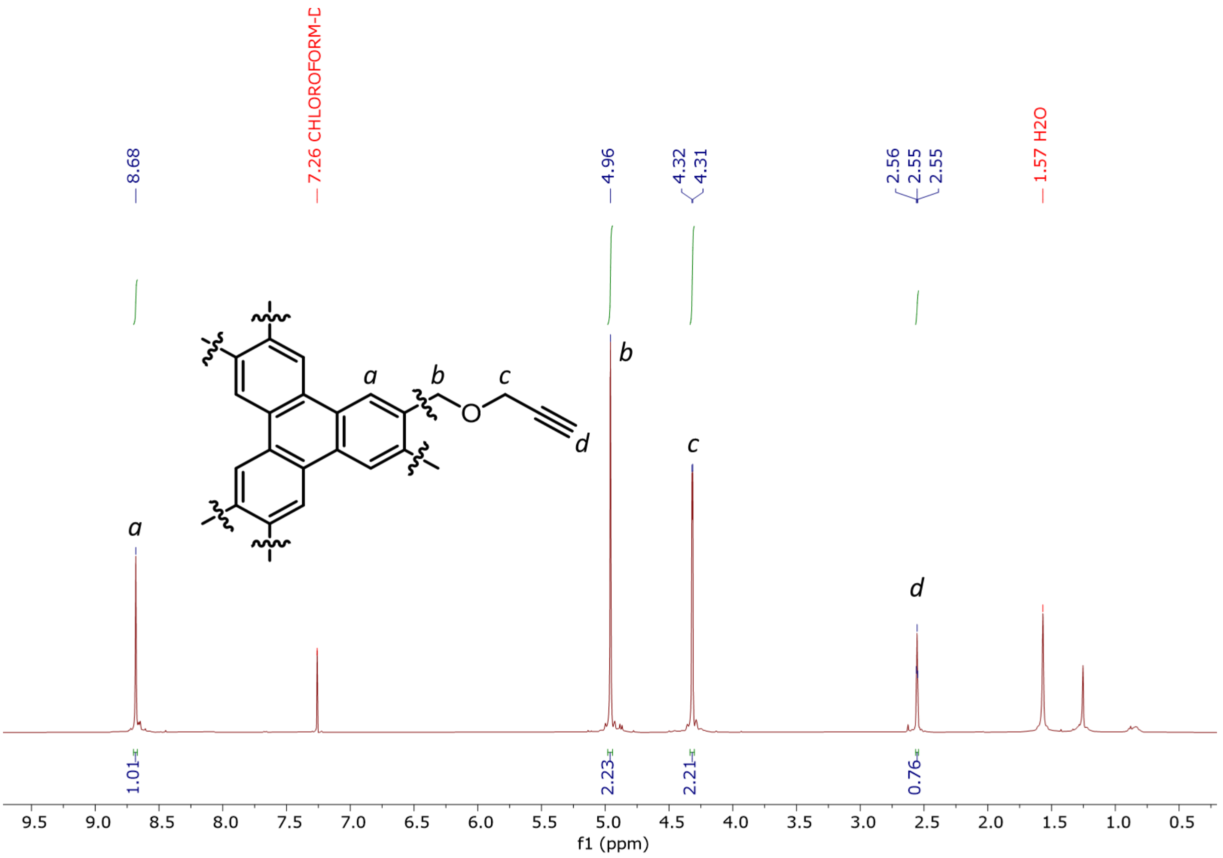

Figure S34. ^{1}H NMR spectrum of compound **12** (400 MHz, $CDCl_3$) with signals assigned.

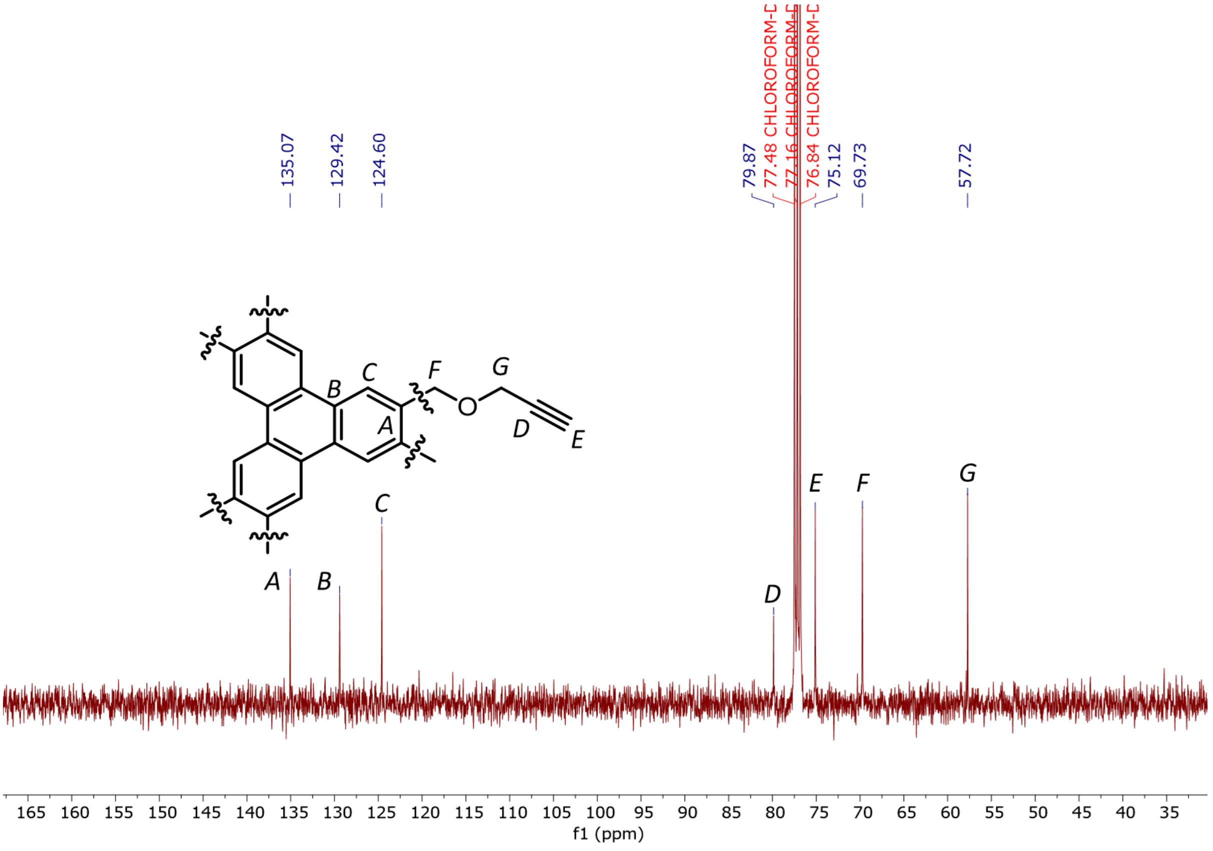

Figure S35. ^{13}C NMR spectrum of compound **12** (101 MHz, $CDCl_3$) with signals assigned.

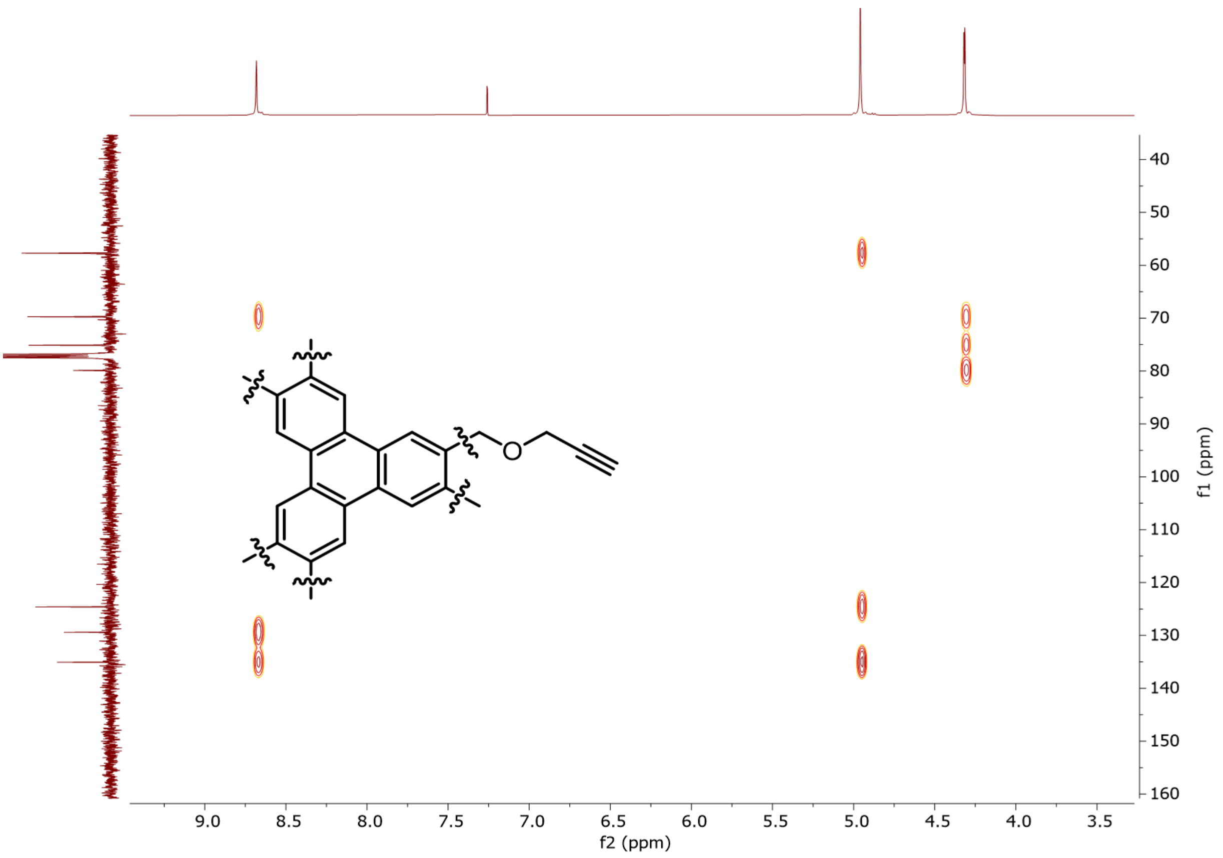

Figure S36. ^{1}H-^{13}C HMBC spectrum of compound **12** ($CDCl_3$).

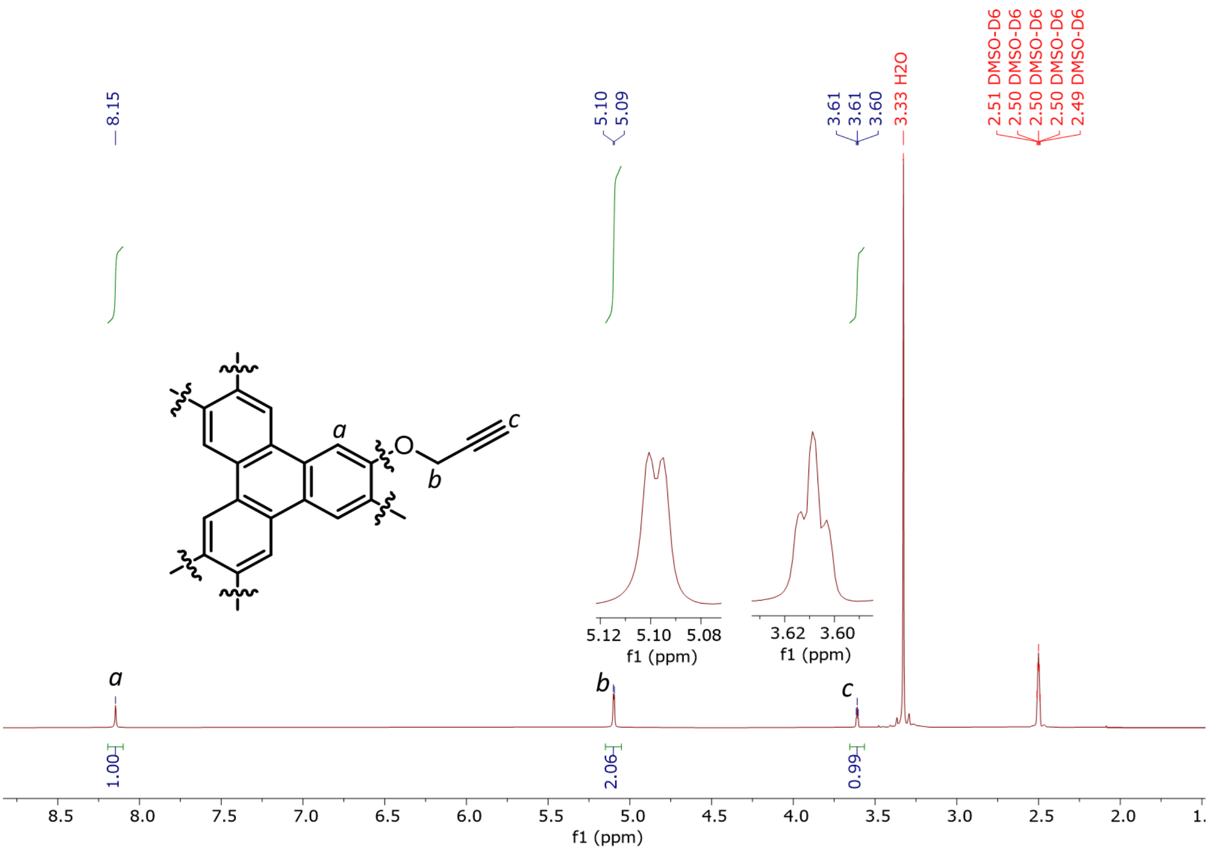

Figure S37. ^{1}H NMR spectrum of compound **14** (400 MHz, DMSO-d_6) with signals assigned.

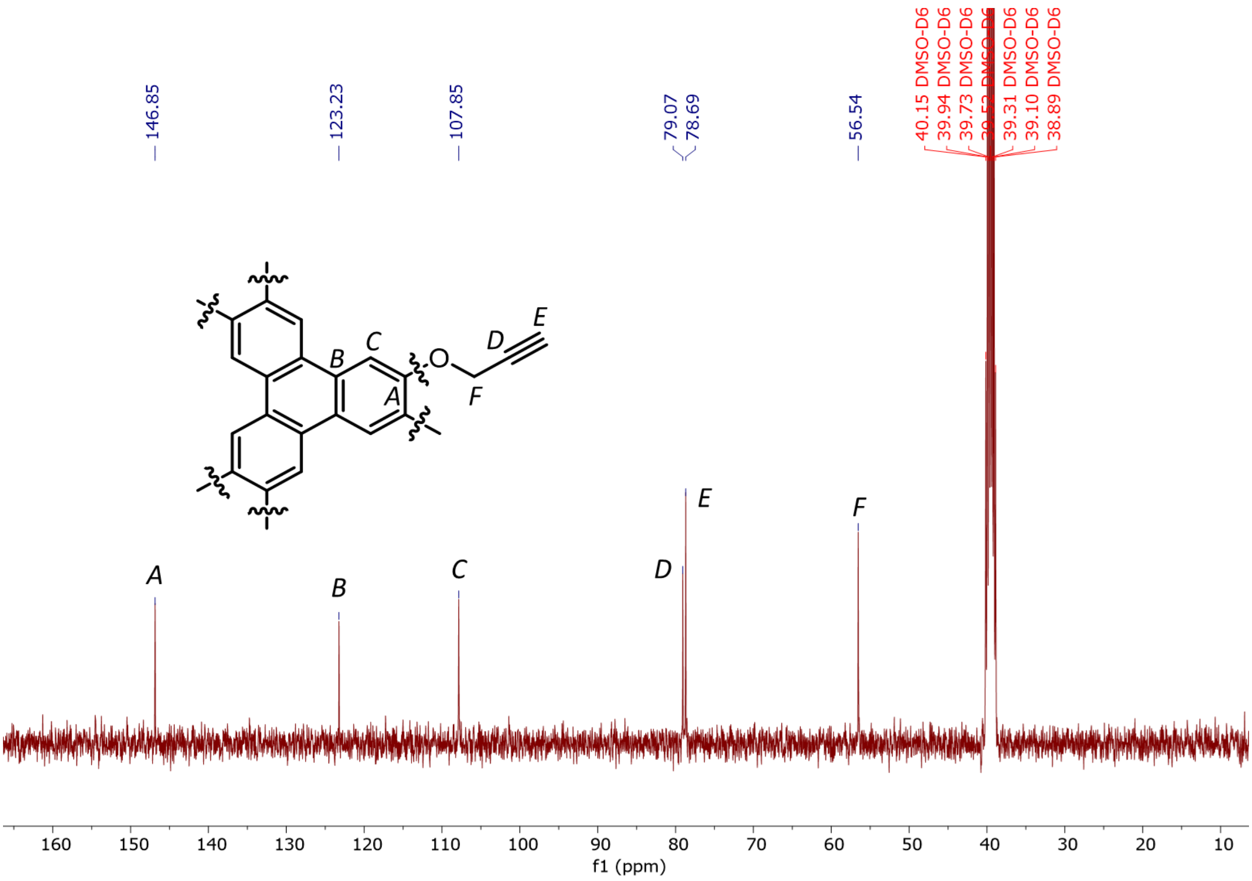

Figure S38. ^{13}C NMR spectrum of compound **14** (101 MHz, DMSO-d_6) with signals assigned.

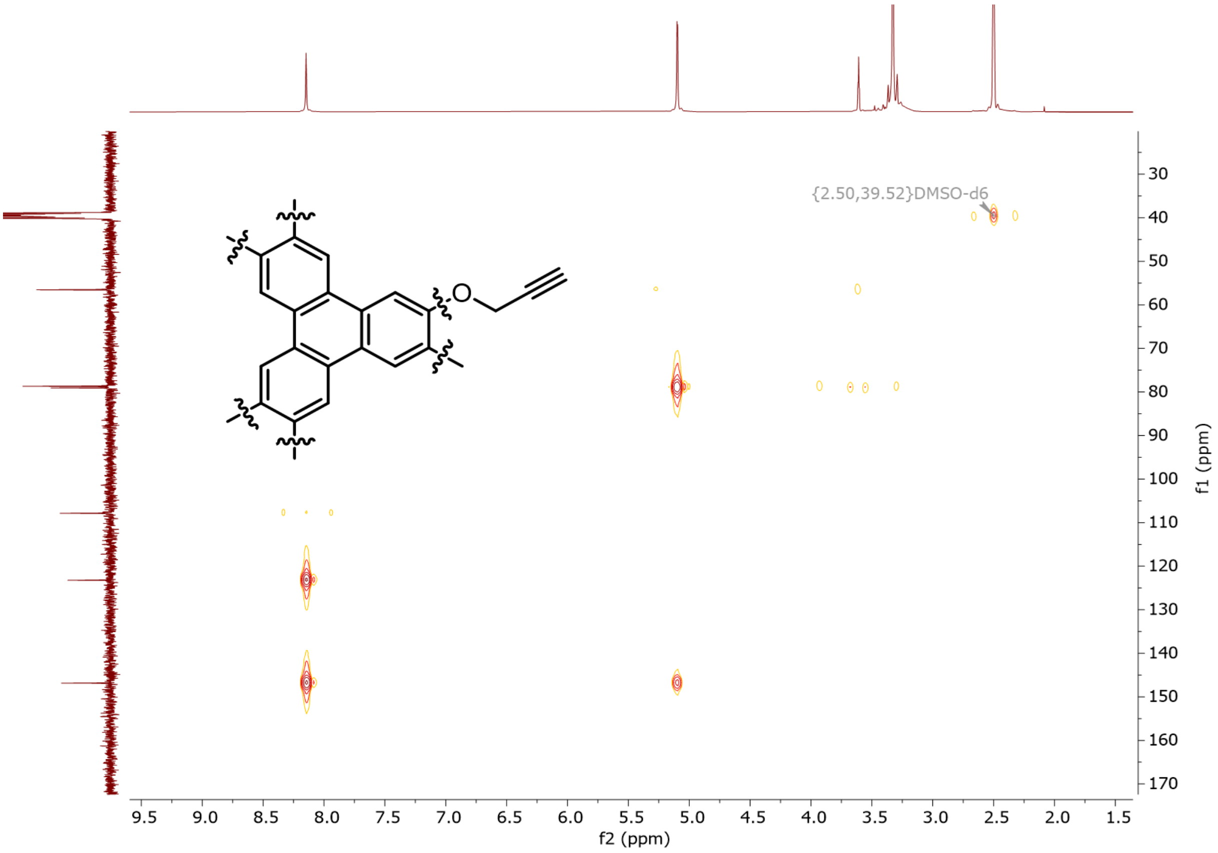

Figure S39. ^1H-^{13}C HMBC spectrum of compound **14** (DMSO-d_6).

S4. Thermal ellipsoid plot of 9

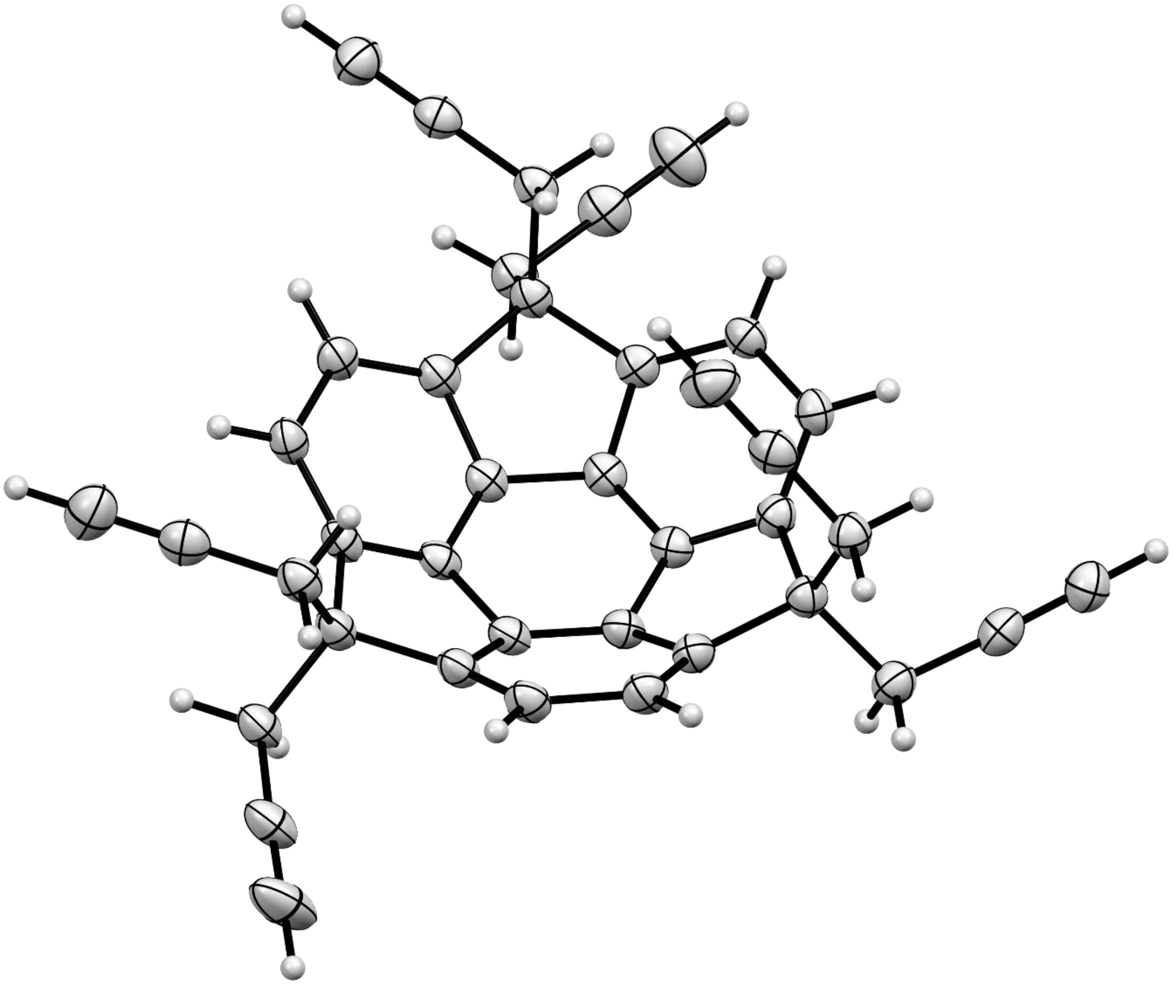

Figure S40. Thermal ellipsoid plot for crystal structure of **9**. Ellipsoids were generated at 50% probability level.

Cell:	a=8.5741(2)	b=12.5352(3)	c=14.2326(3)
	alpha=113.160(2)	beta=106.245(2)	gamma=90.217(2)
Temperature:	123 K		

	Calculated	Reported
Volume	1338.99(6)	1338.99(6)
Space group	P -1	P -1
Hall group	-P 1	-P 1
Moiety formula	C39 H24	C39 H24
Sum formula	C39 H24	C39 H24
Mr	492.58	492.58
Dx,g cm-3	1.222	1.222
Z	2	2
Mu (mm-1)	0.526	0.526
F000	516.0	516.0
F000’	517.35	
h,k,lmax	10,15,17	10,15,17
Nref	5530	5308
Tmin,Tmax	0.945,0.959	0.964,1.000
Tmin’	0.934	

Correction method= # Reported T Limits: Tmin=0.964 Tmax=1.000
AbsCorr = MULTI-SCAN

Data completeness= 0.960 Theta(max)= 75.160

R(reflections)= 0.0397(4566) wR2(reflections)= 0.1100(5308)

S = 1.038 Npar= 352

Figure S41. Selected parameters of crystal structure of **9**.

S5. Determination of molar absorption coefficient

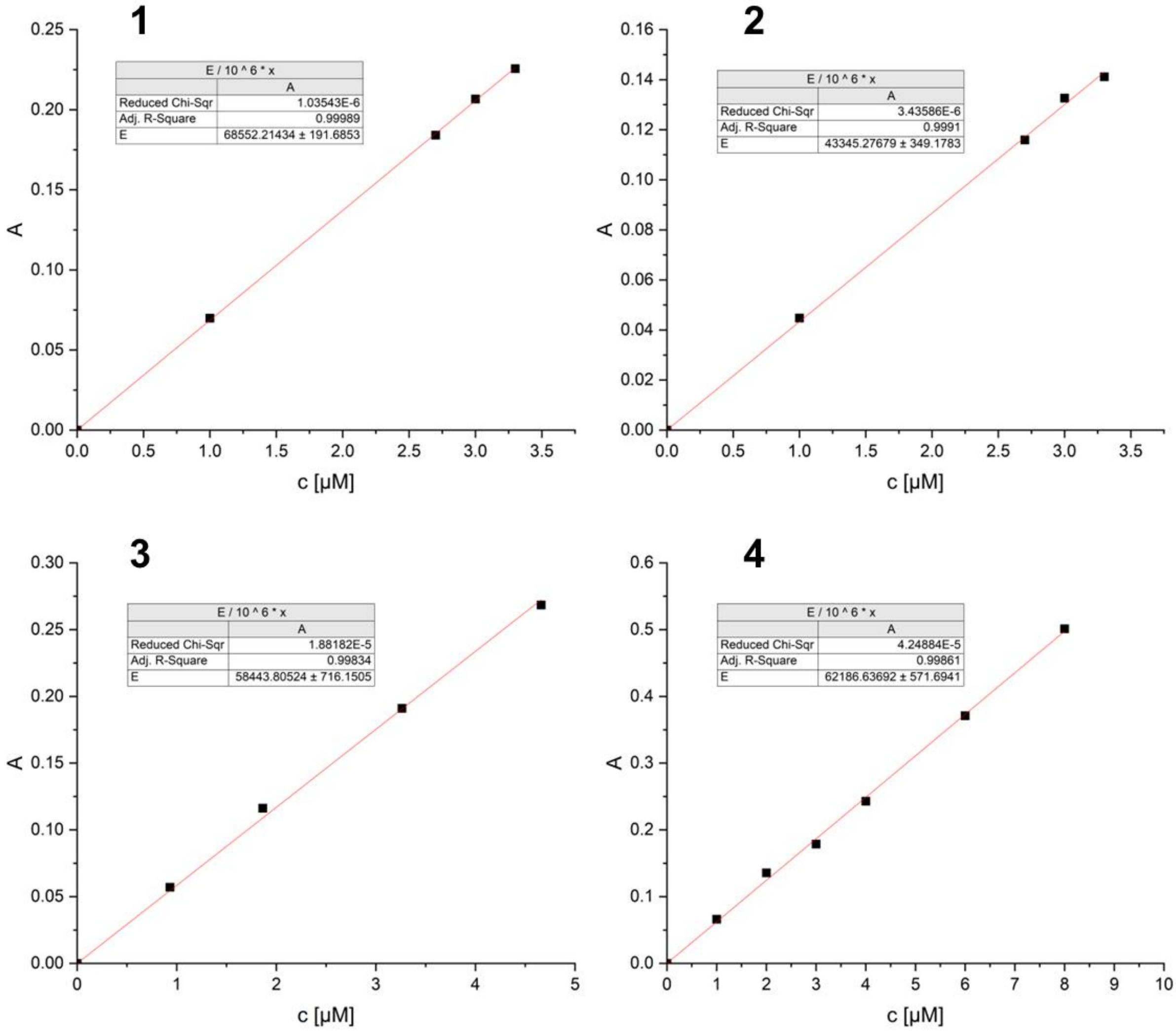

Plot S1. Absorbance in the function of concentration of compounds **1-4** in water. Lambert-Beer equation fitting curves, parameters of the linear regression, as well as calculated molar absorption coefficient values were also shown.

S5. Cation binding experiments

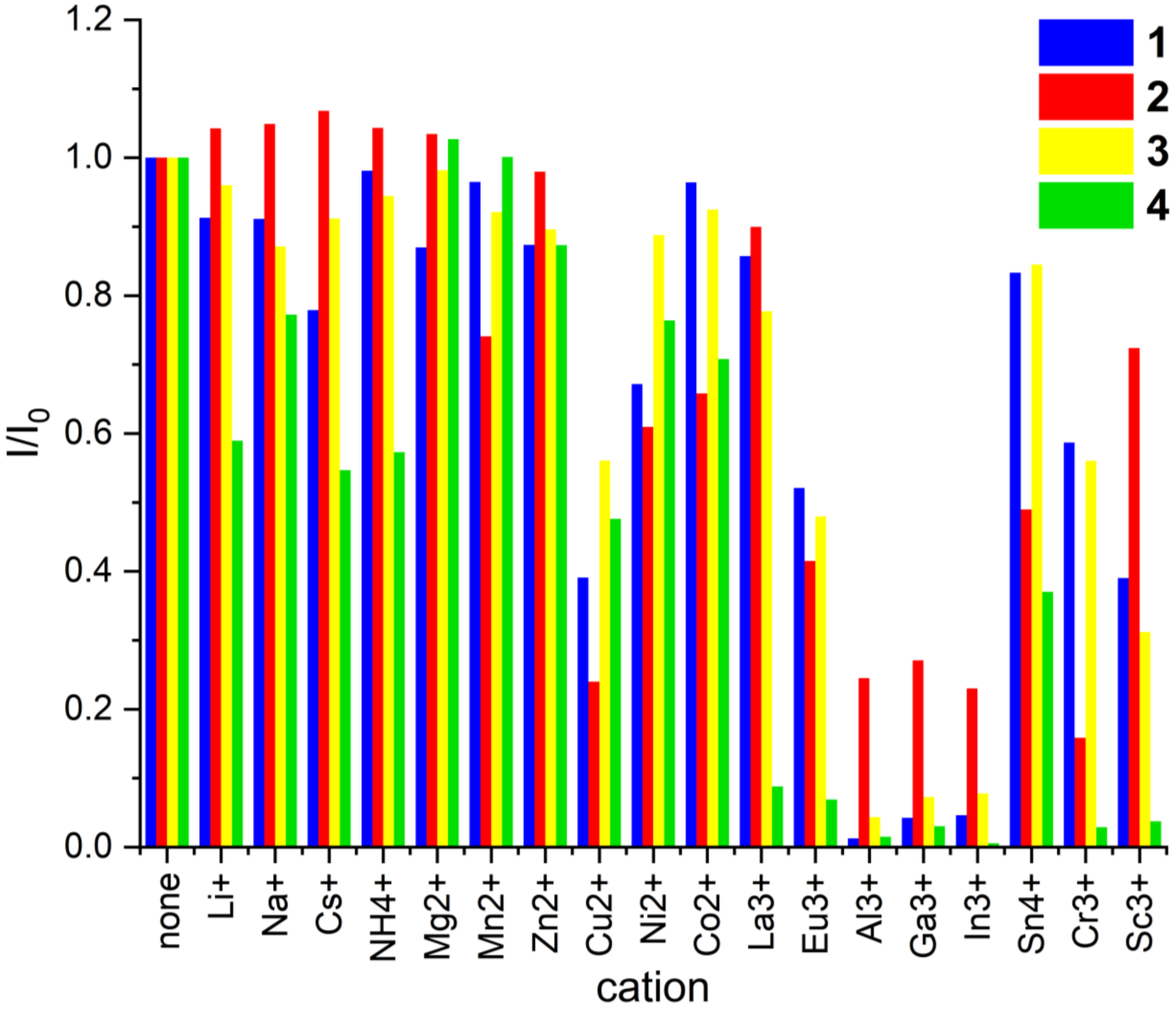

Plot S2. Fluorescence intensity of compounds **1-4** in presence of different cations. Receptor concentration: 0.1 μM, ion concentration: 5.0 μM, solvent: H_2O, buffer: AcOH/Tris pH 5.0 (1 mM).

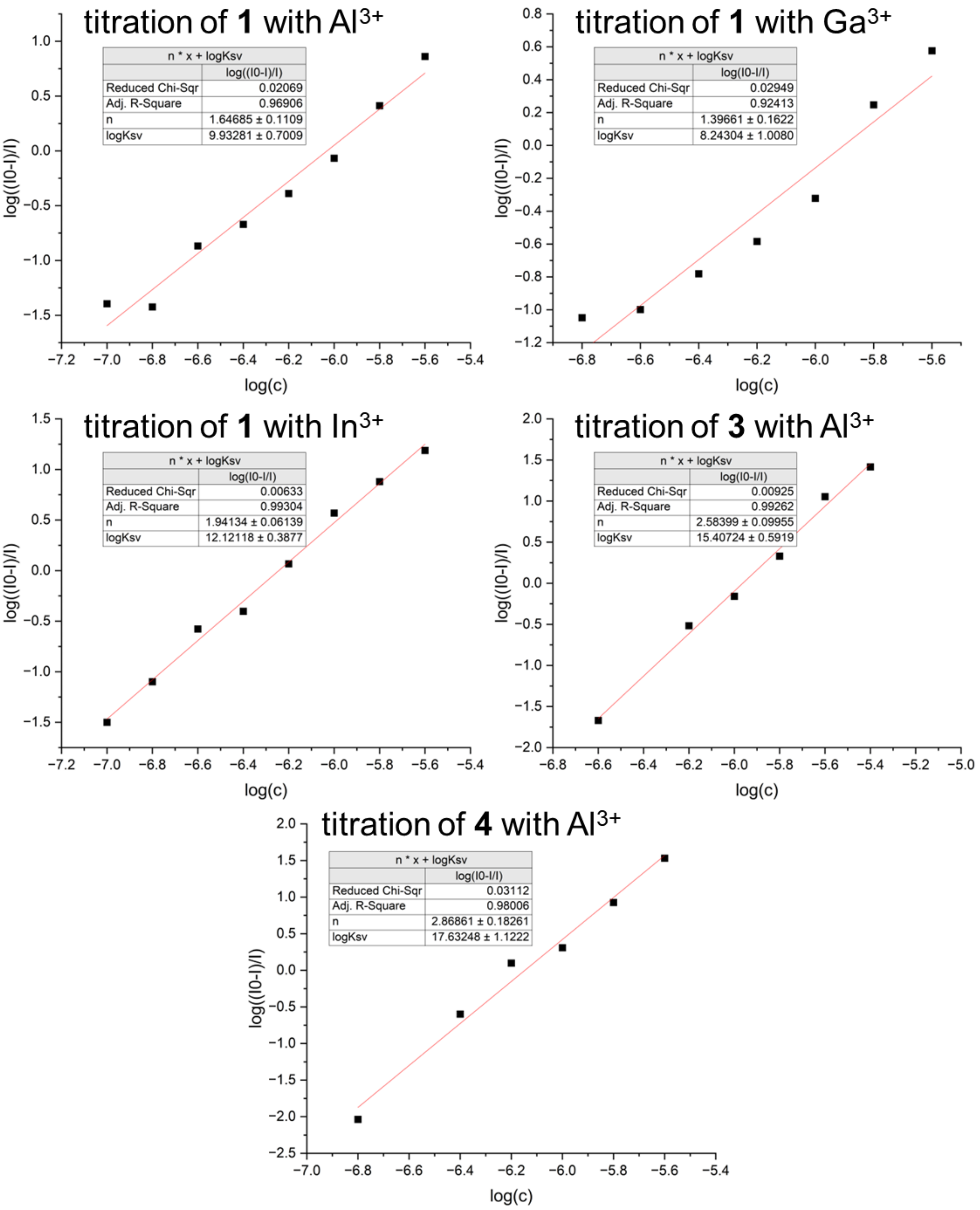

Plot S3. Modified Stern-Volmer plots for the emission quenching of compounds **1**, **3** and **4** by the addition of metal cations. Parameters of the linear regression, as well as calculated modified Stern-Volmer constants (K_{SV}) and average quencher number (n) values are also provided.

S6. Real-life water sample characterization

Table S2. ICP/AES results for samples **1-6**.

sample number	Al^{3+} concentration [ppb]
**1**	83.0
**2**	43.7
**3**	87.1
**4**	20.3
**5**	10.4
**6**	4.9

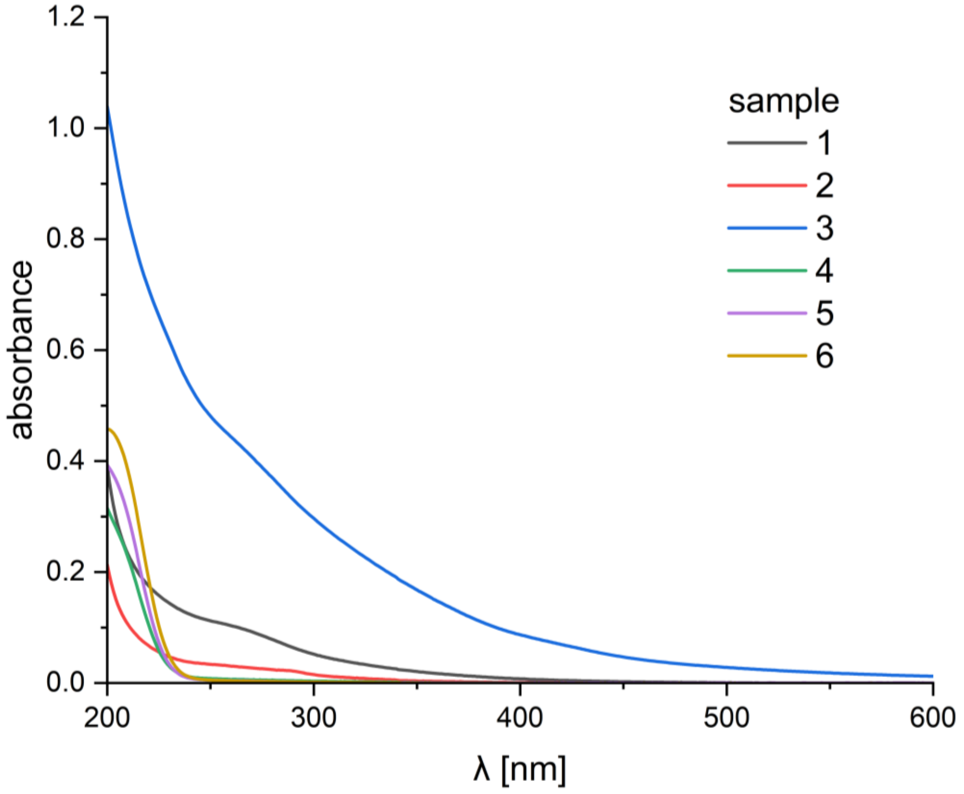

Plot S4. Absorption spectra of real-life water samples 1-6 without compound **1** added.

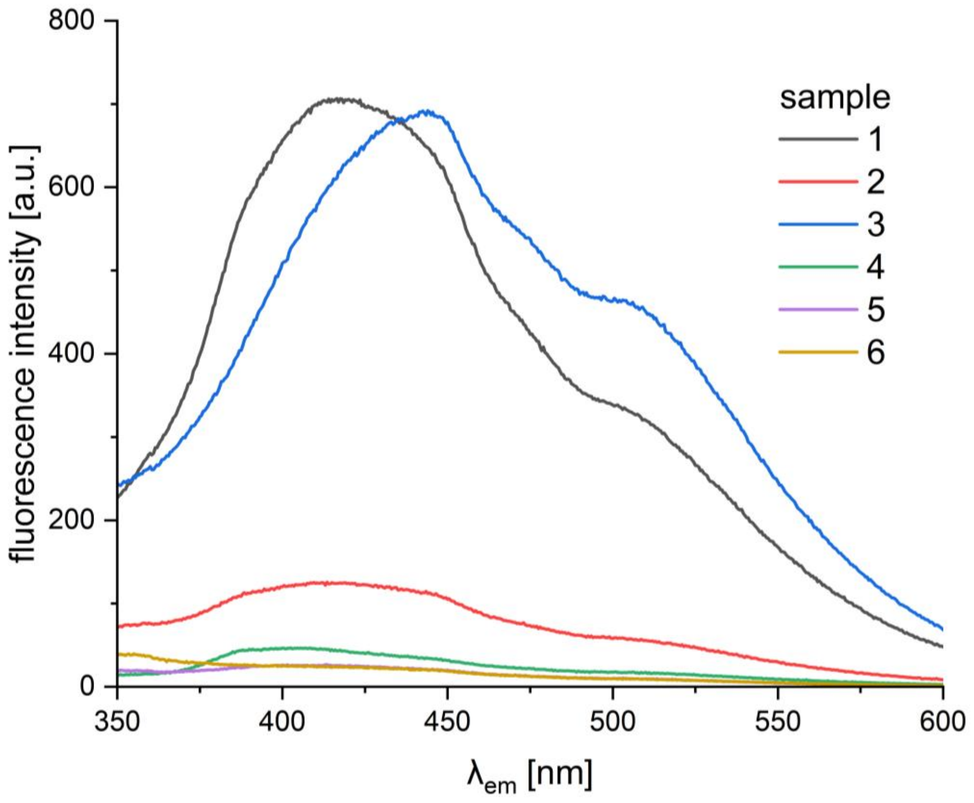

Plot S5. Fluorescence spectra of real-life water samples without compound **1** added.

S7. Free energy of dimerization profiles

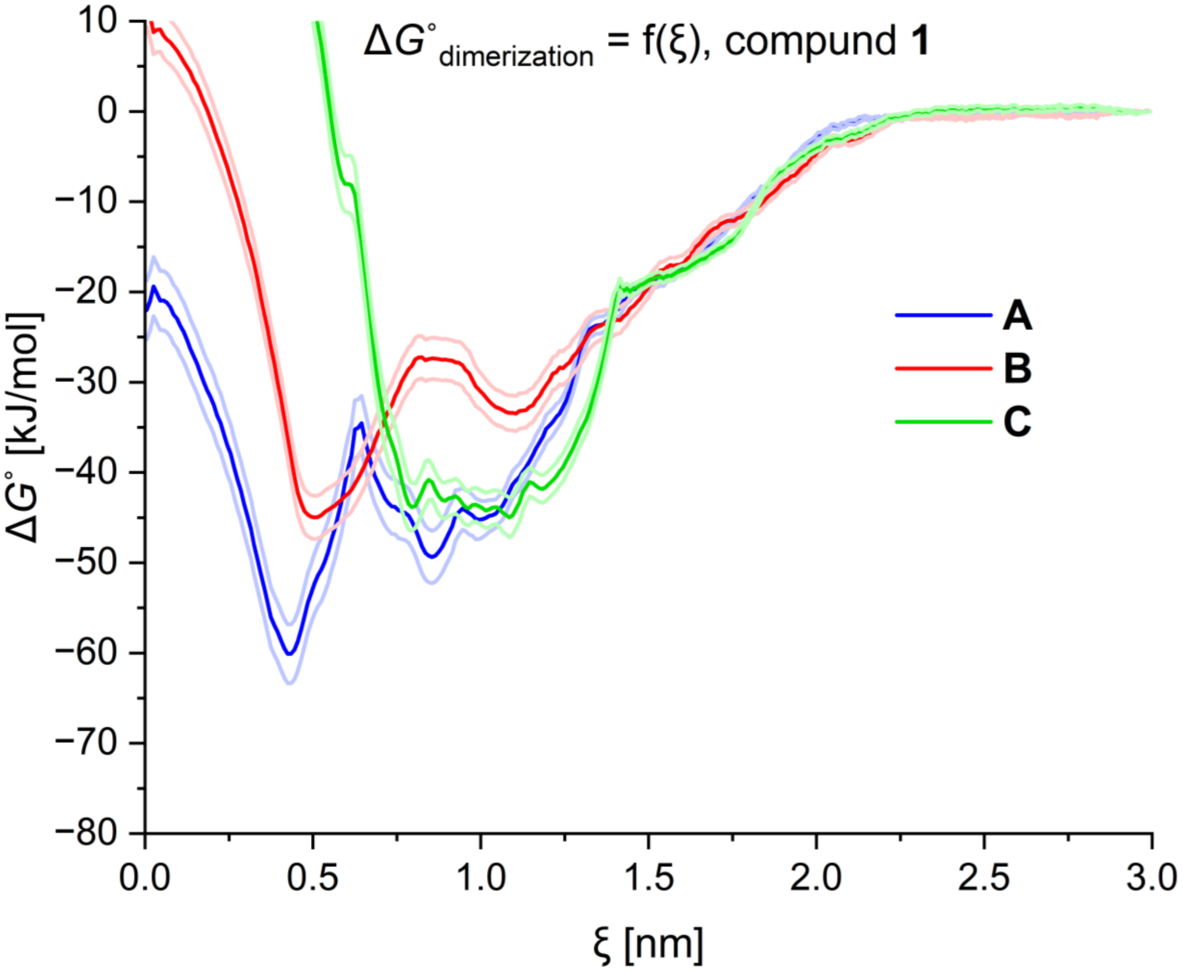

Plot S6. Free energy of dimerization of **1**, as a function of reaction coordinate. Different dimer types were plotted separately (A: convex-to-concave, B: convex-to-convex, C: concave-to-concave). Energy values were plotted in solid colors (solid blue, solid red, solid green) and uncertainty ranges were plotted in light colors (light blue, light red, light green).

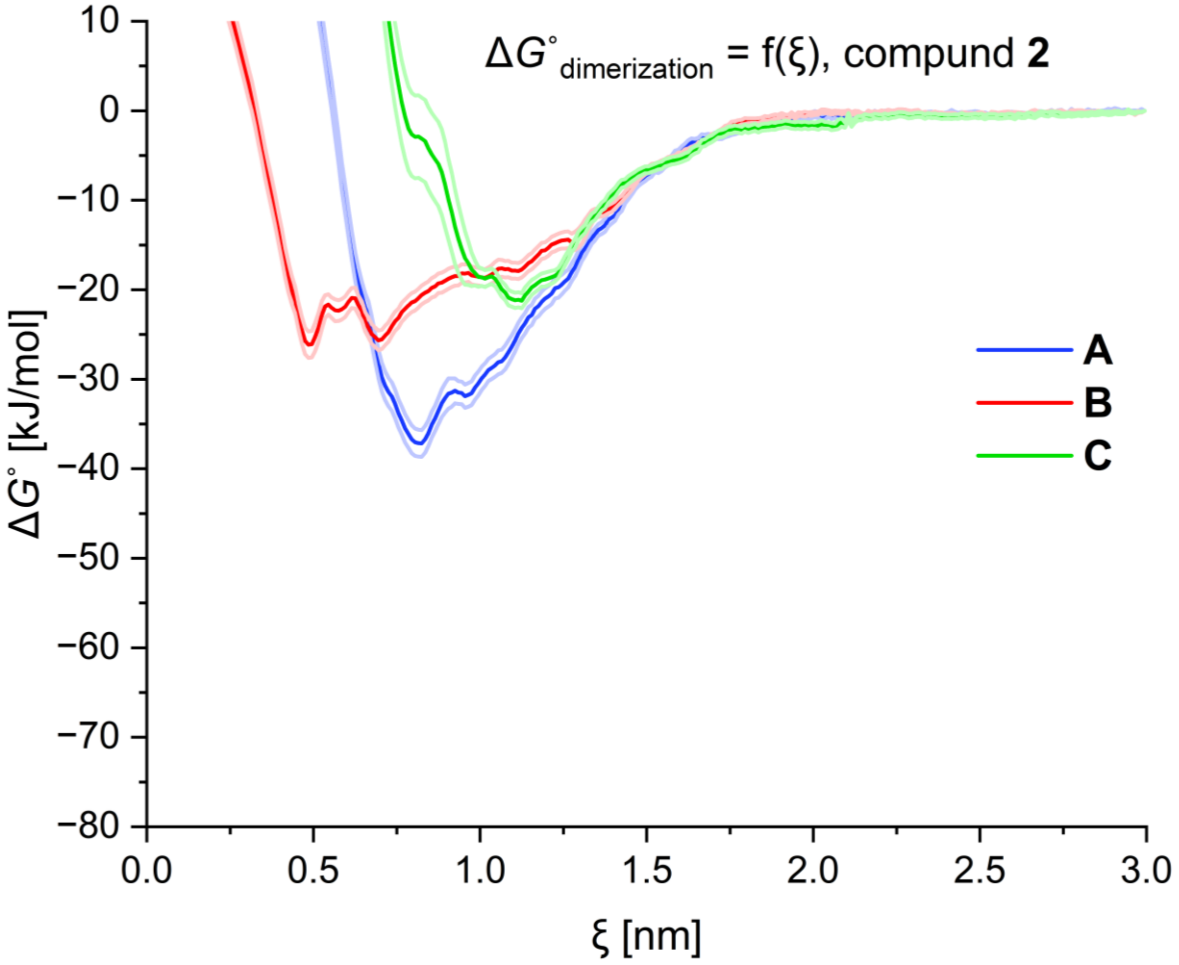

Plot S7. Free energy of dimerization of **2**, as a function of reaction coordinate. Different dimer types were plotted separately (A: convex-to-concave, B: convex-to-convex, C: concave-to-concave). Energy values were plotted in solid colors (solid blue, solid red, solid green) and uncertainty ranges were plotted in light colors (light blue, light red, light green).

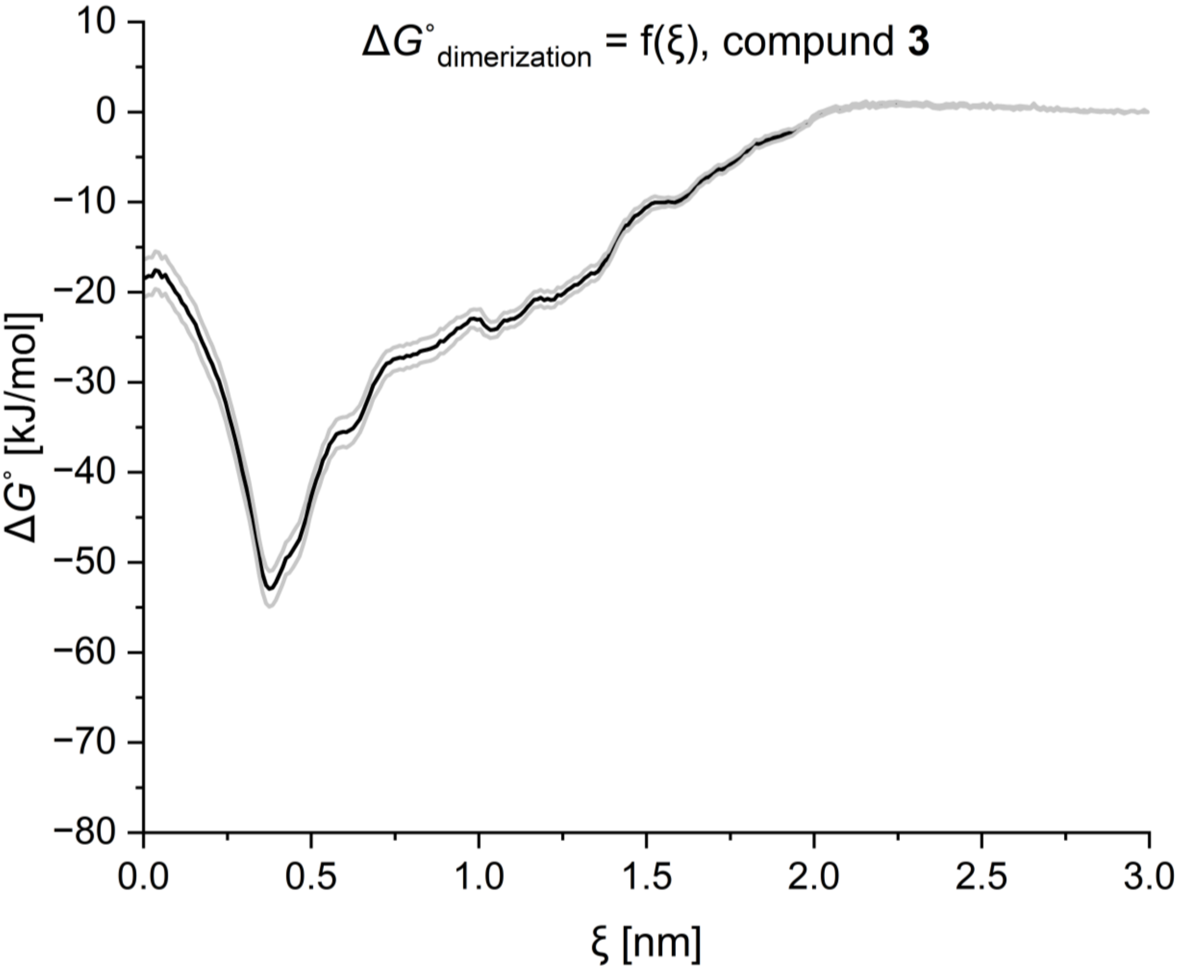

Plot S8. Free energy of dimerization of **3**, as a function of reaction coordinate. Energy values were plotted in black, and uncertainty range was plotted in grey.

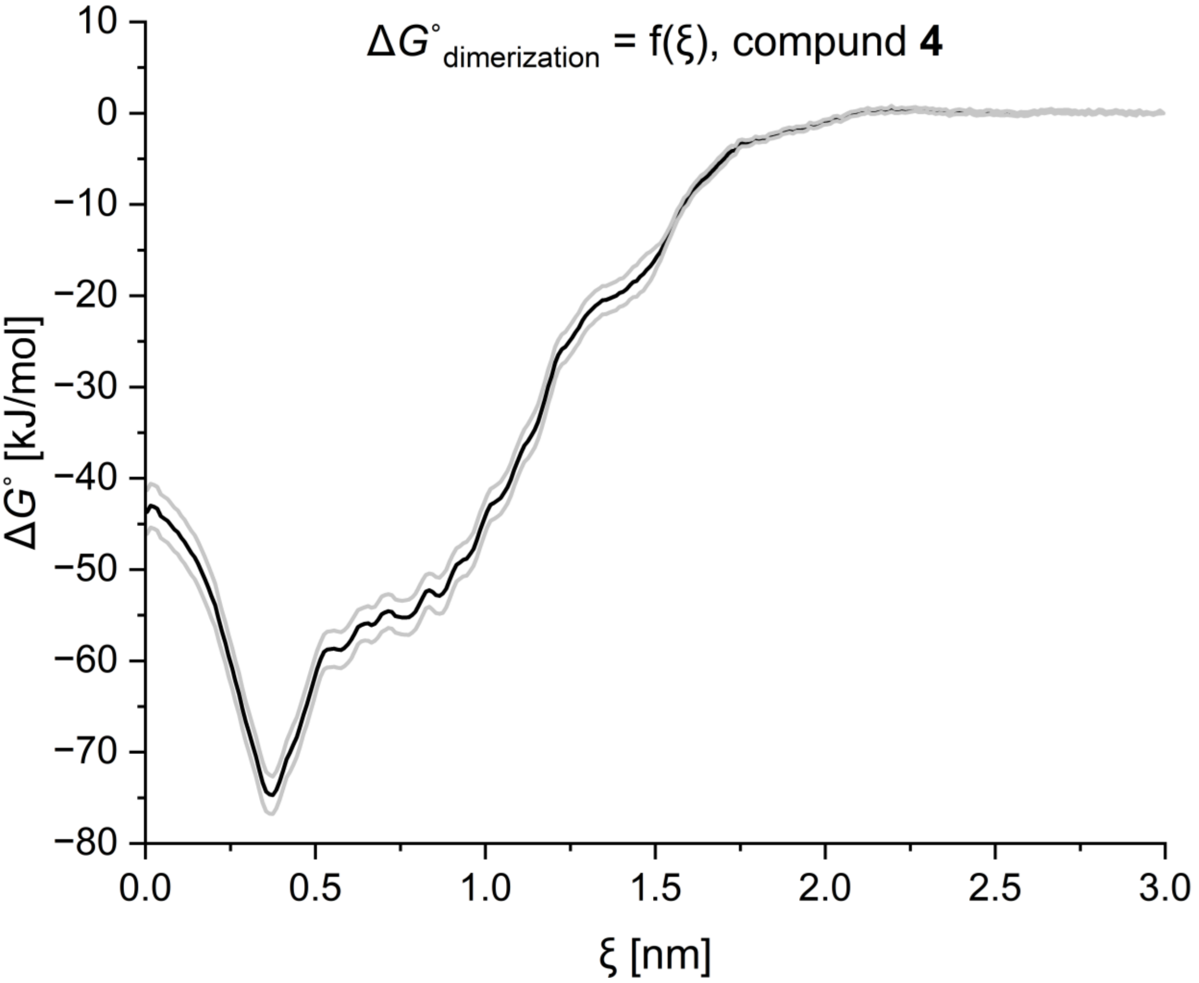

Plot S9. Free energy of dimerization of **4**, as a function of reaction coordinate. Energy values were plottesd in black, and uncertainty range was plotted in grey.

S8. Literature survey

Results of literature search on group 13 cation receptors were summarized in Table S3. To construct the table, we analyzed the publications from Google Scholar database, found using the following queries:

a) *"aqueous medium" OR "water-soluble" OR "water soluble" receptor fluorescent Al3+* . This query was used to find the publications on water-soluble fluorescent Al^{3+} receptors. As numerous such receptors were reported (over 50 were reported in 2024 only), 10 publications from the last year (2024) were randomly chosen and analyzed.

b) *"aqueous medium" OR "water-soluble" OR "water soluble" receptor fluorescent Ga3+* . This query was used to find the publications on water-soluble fluorescent Ga^{3+} receptors. 10 most recent publications were analyzed. These publications were published in years 2022-2024.

c) *"aqueous medium" OR "water-soluble" OR "water soluble" receptor fluorescent In3+* . This query was used to find the publications on water-soluble fluorescent In^{3+} receptors. Only seven publications on such receptors appear to have ever been published. As none of these analyzed publications on In^{3+} receptors mentioned the ability to detect Ga^{3+} and Al^{3+} alongside In^{3+}, we believe that this work is the first to report the detection of In^{3+}, Ga^{3+} and Al^{3+} using one fluorescent receptor in aqueous media.

Table S3. Results of the literature survey.

literature reference	detected ions	LOD [nM]	solvent	ion-binding moiety
37	Al^{3+}	330	DMSO:H_2O 1:1	Schiff base
38	Al^{3+}	560	H_2O	Schiff base
39	Al^{3+}	1900	H_2O	sulphonamide
40	Al^{3+}	153	DMSO:H_2O 4:1	Schiff base
41	Al^{3+}	41.7	DMF:H_2O 1:1	amide
42	Al^{3+}	82	H_2O	Schiff base
43	Al^{3+}	202	DMSO:H_2O 7:3	Schiff base
44	Al^{3+}	47.6	MeOH:H_2O 1:4	Schiff base
45	Ga^{3+}	34	DMSO:H_2O 9:1	Schiff base
46	Ga^{3+}	1300	MeOH:H_2O 95:5	Schiff base
47	Ga^{3+}	14	DMF:H_2O 9:1	Schiff base
48	Ga^{3+}	11.5	DMSO:H_2O 9:1	Schiff base
49	Ga^{3+}	3.9	MeCN:H_2O 9:1	Schiff base
50	Ga^{3+}	3.1	DMSO:H_2O 9:1	Schiff base
51	Ga^{3+}	48.8	DMSO:H_2O 9:1	Schiff base
52	In^{3+}	62.7	DMSO:H_2O 1:4	Schiff base
53	In^{3+}	1690	H_2O	Schiff base
54	In^{3+}	5.9	DMSO:H_2O 7:3	Schiff base
55	In^{3+}	0.18	DMF:H_2O 9:1	Schiff base
56	In^{3+}	2700	H_2O	Schiff base
57	Al^{3+}, Fe^{3+}	11.7 (Al^{3+})	DMSO:H_2O 9:1	Schiff base
58	Al^{3+}, Hg^{2+}	4.3 (Al^{3+})	DMSO:H_2O 2:3	Schiff base
59	Al^{3+}, Ga^{3+}	1500 (Al^{3+})<br>4300 (Ga^{3+})	DMF:H_2O 2:3	Schiff base
60	Al^{3+}, Ga^{3+}	48.6 (Al^{3+})<br>20.0 (Ga^{3+})	[a] MeOH:H_2O 9:1 (Al^{3+})<br>DMSO:H_2O 9:1 (Ga^{3+})	Schiff base
61	Ga^{3+}, Al^{3+}	201 (Ga^{3+})<br>61 (Al^{3+})	H_2O	Schiff base
62	Ga^{3+}, In^{3+}	4.4 (Ga^{3+}),<br>0.02 (In^{3+})	[a] DMSO:H_2O 9:1 (Ga^{3+})<br>DMF:H_2O 9:1 (In^{3+})	Schiff base
63	In^{3+}, Pb^{2+}	8.4 (In^{3+})	DMF:H_2O 9:1	Schiff base
**this work**	**Al^{3+}, Ga^{3+}, In^{3+}**	**240 (Al^{3+})<br>370 (Ga^{3+})<br>90 (In^{3+})**	**H_2O**	**carboxyl group**

[a]Different solvent systems were used for detection of different ions.

S9. Supporting references

(1) Sakurai, H.; Daiko, T.; Hirao, T. A Synthesis of Sumanene, a Fullerene Fragment. *Science (80-.).* **2003**, *301* (5641), 1878–1878. https://doi.org/10.1126/science.1088290.

(2) Cai, J.; Li, X.; Yue, X.; Taylor, J. S. Nucleic Acid-Triggered Fluorescent Probe Activation by the Staudinger Reaction. *J. Am. Chem. Soc.* **2004**, *126* (50), 16324–16325. https://doi.org/10.1021/ja0452626.

(3) Viel, S.; Ziarelli, F.; Pagès, G.; Carrara, C.; Caldarelli, S. Pulsed Field Gradient Magic Angle Spinning NMR Self-Diffusion Measurements in Liquids. *J. Magn. Reson.* **2008**, *190* (1), 113–123. https://doi.org/10.1016/j.jmr.2007.10.010.

(4) Kunde, T.; Nieland, E.; Schröder, H. V.; Schalley, C. A.; Schmidt, B. M. A Porous Fluorinated Organic [4+4] Imine Cage Showing CO 2 and H 2 Adsorption. *Chem. Commun.* **2020**, *56* (35), 4761–4764. https://doi.org/10.1039/D0CC01872D.

(5) CrysAlis PRO. Rigaku Oxford Diffraction, Yarnton, England 2014.

(6) Sheldrick, G. M. SHELXT – Integrated Space-Group and Crystal-Structure Determination. *Acta Crystallogr. Sect. A Found. Adv.* **2015**, *71* (1), 3–8. https://doi.org/10.1107/S2053273314026370.

(7) Sheldrick, G. M. Crystal Structure Refinement with SHELXL. *Acta Crystallogr. Sect. C Struct. Chem.* **2015**, *71* (1), 3–8. https://doi.org/10.1107/S2053229614024218.

(8) Dolomanov, O. V.; Bourhis, L. J.; Gildea, R. J.; Howard, J. A. K.; Puschmann, H. OLEX2 : A Complete Structure Solution, Refinement and Analysis Program. *J. Appl. Crystallogr.* **2009**, *42* (2), 339–341. https://doi.org/10.1107/S0021889808042726.

(9) Lakowicz, J. R. *Principles of Fluorescence Spectroscopy*, 3rd ed.; 2006.

(10) Bardhan, M.; Mandal, G.; Ganguly, T. Steady State, Time Resolved, and Circular Dichroism Spectroscopic Studies to Reveal the Nature of Interactions of Zinc Oxide Nanoparticles with Transport Protein Bovine Serum Albumin and to Monitor the Possible Protein Conformational Changes. *J. Appl. Phys.* **2009**, *106* (3). https://doi.org/10.1063/1.3190483.

(11) Basak, P.; Debnath, T.; Banerjee, R.; Bhattacharyya, M. Selective Binding of Divalent Cations toward Heme Proteins. *Front. Biol. (Beijing).* **2016**, *11* (1), 32–42. https://doi.org/10.1007/s11515-016-1388-0.

(12) Rajapakshe, B. U.; Li, Y.; Corbin, B.; Wijesinghe, K. J.; Pang, Y.; Abeywickrama, C. S. Copper-Induced Fluorescence Quenching in a Bis[2-(2′-Hydroxyphenyl)Benzoxazole]Pyridinium Derivative for Quantification of Cu2+ in Solution. *Chemosensors* **2022**, *10* (10), 382. https://doi.org/10.3390/chemosensors10100382.

(13) Abraham, M. J.; Murtola, T.; Schulz, R.; Páll, S.; Smith, J. C.; Hess, B.; Lindah, E. Gromacs: High Performance Molecular Simulations through Multi-Level Parallelism from Laptops to Supercomputers. *SoftwareX* **2015**, *1–2*, 19–25. https://doi.org/10.1016/j.softx.2015.06.001.

(14) Berendsen, H. J. C.; van der Spoel, D.; van Drunen, R. GROMACS: A Message-

Passing Parallel Molecular Dynamics Implementation. *Comput. Phys. Commun.* **1995**, *91* (1–3), 43–56. https://doi.org/10.1016/0010-4655(95)00042-E.

(15) Hanwell, M. D.; Curtis, D. E.; Lonie, D. C.; Vandermeersch, T.; Zurek, E.; Hutchison, G. R. Avogadro: An Advanced Semantic Chemical Editor, Visualization, and Analysis Platform. *J. Cheminform.* **2012**, *4* (1), 17. https://doi.org/10.1186/1758-2946-4-17.

(16) Tian, L. Sobtop, Version 1.0, Http://Sobereva.Com/Soft/Sobtop.

(17) Wang, J.; Wolf, R. M.; Caldwell, J. W.; Kollman, P. A.; Case, D. A. Development and Testing of a General Amber Force Field. *J. Comput. Chem.* **2004**, *25* (9), 1157–1174. https://doi.org/10.1002/jcc.20035.

(18) Mortier, W. J.; Van Genechten, K.; Gasteiger, J. Electronegativity Equalization: Application and Parametrization. *J. Am. Chem. Soc.* **1985**, *107* (4), 829–835. https://doi.org/10.1021/ja00290a017.

(19) Berendsen, H. J. C.; Grigera, J. R.; Straatsma, T. P. The Missing Term in Effective Pair Potentials. *J. Phys. Chem.* **1987**, *91* (24), 6269–6271. https://doi.org/10.1021/j100308a038.

(20) Darden, T.; York, D.; Pedersen, L. Particle Mesh Ewald: An N ·log(N) Method for Ewald Sums in Large Systems. *J. Chem. Phys.* **1993**, *98* (12), 10089–10092. https://doi.org/10.1063/1.464397.

(21) Jiang, F. Y.; Bouret, Y.; Kindt, J. T. Molecular Dynamics Simulations of the Lipid Bilayer Edge. *Biophys. J.* **2004**, *87* (1), 182–192. https://doi.org/10.1529/biophysj.103.031054.

(22) Bussi, G.; Donadio, D.; Parrinello, M. Canonical Sampling through Velocity Rescaling. *J. Chem. Phys.* **2007**, *126* (1). https://doi.org/10.1063/1.2408420.

(23) Parrinello, M.; Rahman, A. Crystal Structure and Pair Potentials: A Molecular-Dynamics Study. *Phys. Rev. Lett.* **1980**, *45* (14), 1196–1199. https://doi.org/10.1103/PhysRevLett.45.1196.

(24) Hub, J. S.; De Groot, B. L.; Van Der Spoel, D. G-Whams-a Free Weighted Histogram Analysis Implementation Including Robust Error and Autocorrelation Estimates. *J. Chem. Theory Comput.* **2010**, *6* (12), 3713–3720. https://doi.org/10.1021/ct100494z.

(25) Kumar, S.; Bouzida, D.; Swendsen, R. H.; Kollman, P. A.; Rosenberg, J. M. The Weighted Histogram Analysis Method for Free-Energy Calculations on Biomolecules. *J. Comput. Chem.* **1992**, *13* (8), 1011–1021.

(26) Zhang, Y.; Jiang, Y.; Qiu, Y.; Zhang, H. Rational Design of Nonbonded Point Charge Models for Highly Charged Metal Cations with Lennard-Jones 12-6 Potential. *J. Chem. Inf. Model.* **2021**, *61* (9), 4613–4629. https://doi.org/10.1021/acs.jcim.1c00723.

(27) Sure, R.; Grimme, S. Corrected Small Basis Set Hartree-Fock Method for Large Systems. *J. Comput. Chem.* **2013**, *34* (19), 1672–1685. https://doi.org/10.1002/jcc.23317.

(28) Barone, V.; Cossi, M. Quantum Calculation of Molecular Energies and Energy Gradients in Solution by a Conductor Solvent Model. *J. Phys. Chem. A* **1998**, *102* (11), 1995–2001. https://doi.org/10.1021/jp9716997.

(29) Neese, F. The ORCA Program System. *WIREs Comput. Mol. Sci.* **2012**, *2* (1), 73–78. https://doi.org/10.1002/wcms.81.

(30) Nakazawa Yuta; Yakiyama, Yumi; Sakurai, Hidehiro, H. U. Pentagon-Fused Sumanenes on the Aromatic Peripheries En Route to the Bottom-Up Synthesis of Fullerenes. *Synlett* **2023**, *34* (04), 374–378. https://doi.org/10.1055/a-1992-0487.

(31) Amaya, T.; Sakane, H.; Muneishi, T.; Hirao, T. Bowl-to-Bowl Inversion of Sumanene Derivatives. *Chem. Commun.* **2008**, *2* (6), 765–767. https://doi.org/10.1039/b712839h.

(32) Osawa, T.; Kajitani, T.; Hashizume, D.; Ohsumi, H.; Sasaki, S.; Takata, M.; Koizumi, Y.; Saeki, A.; Seki, S.; Fukushima, T.; Aida, T. Wide-Range 2D Lattice Correlation Unveiled for Columnarly Assembled Triphenylene Hexacarboxylic Esters. *Angew. Chemie - Int. Ed.* **2012**, *51* (32), 7990–7993. https://doi.org/10.1002/anie.201203077.

(33) Inamoto, K.; Yamamoto, A.; Ohsawa, K.; Hiroya, K.; Sakamoto, T. Highly Regioselective Palladium-Catalyzed Annulation Reactions of Heteroatom-Substituted Allenes for Synthesis of Condensed Heterocycles. *Chem. Pharm. Bull.* **2005**, *53* (11), 1502–1507. https://doi.org/10.1248/cpb.53.1502.

(34) Zniber, R.; Achour, R.; Cherkaoui, M. Z.; Donnio, B.; Gehringer, L.; Guillon, D. Columnar Mesophase from a New Hybrid Siloxane-Triphenylene. *J. Mater. Chem.* **2002**, *12* (8), 2208–2213. https://doi.org/10.1039/b202677e.

(35) Wayment, L. J.; Wang, X.; Huang, S.; McCoy, M. S.; Chen, H.; Hu, Y.; Jin, Y.; Sharma, S.; Zhang, W. 3D Covalent Organic Framework as a Metastable Intermediate in the Formation of a Double-Stranded Helical Covalent Polymer. *J. Am. Chem. Soc.* **2023**, *145* (28), 15547–15552. https://doi.org/10.1021/jacs.3c04734.

(36) Boger, D. L.; Palanki, M. S. S. Functional Analogs of CC-1065 and the Duocarmycins Incorporating the 9a-(Chloromethyl)-1,2,9,9a-Tetrahydrocyclopropa[c]Benz[e]Indol-4-One (C2BI) Alkylation Subunit: Synthesis and Preliminary DNA Alkylation Studies. *J. Am. Chem. Soc.* **1992**, *114* (24), 9318–9327. https://doi.org/10.1021/ja00050a012.

(37) Tohora, N.; Ahamed, S.; Sahoo, R.; Mahato, M.; Sultana, T.; Lama, S.; Maiti, A.; Das, S. K. Solid-State Brightness and Al3+ Ions-Triggered Flower-Shaped Nano-Luminogen for Cascade Detection of Al3+ and PO43− Ions. *Opt. Mater. (Amst).* **2024**, *155*, 115803. https://doi.org/10.1016/j.optmat.2024.115803.

(38) Apiratikul, N.; Bunrit, P.; Jommaroeng, S.; Boonsri, P.; Songsrirote, K. Synthesis and Application of Schiff Base as a Dual-Mode Chemosensor for Optical Determination of Aluminium Ion Content in Water Samples. *Sensors Int.* **2025**, *6*, 100313. https://doi.org/10.1016/j.sintl.2024.100313.

(39) Davi, L. B. O.; Costa, A. S. P. N.; Silva, M. S.; Ribeiro, A. S.; Barbosa, C. D. A. E. S.; Da Silva, J. C. S.; Lima, D. J. P.; Anunciação, D. S. Dansyl Derivative Turn off Fluorescent Probe for Al3+: Experimental and Computational Study in Aqueous Medium. *Polyhedron* **2024**, *247*, 116706. https://doi.org/10.1016/j.poly.2023.116706.

(40) Pudi, A.; Hsiao, Y.-S.; Lee, M.-C.; Chang, C.-J. An Azo-Schiff Base

Chemosensor for Selective Turn-on Fluorescent Sensing of Al3+ in Antacid Tablets and Cell-Imaging Applications. *J. Photochem. Photobiol. A Chem.* **2024**, *451*, 115532. https://doi.org/10.1016/j.jphotochem.2024.115532.

(41) Jiang, Q.; Song, J.; Yang, X.; Rao, X.; Zhao, P.; Wang, Z. A Novel Reversible Fluorescent Probe for Sequential Detection of Al3+ and HPO42− Based on Caffeic Acid and Its Applicability in Cell Imaging. *Spectrochim. Acta Part A Mol. Biomol. Spectrosc.* **2024**, *307*, 123627. https://doi.org/10.1016/j.saa.2023.123627.

(42) Shaikh, A.; Shaikh, J.; Ansari, D.; Sheikh, K. N.; Tambe, P.; Shaikh, S.; Lande, D. N.; Gejji, S. P.; Ahmed, K. A 'Turn-on' Fluorescence Sensor Based on 5-Methoxy Salicylaldehyde Hydrazone for Selective Detection of Al3+ Ions. *J. Mol. Struct.* **2025**, *1328*, 141317. https://doi.org/10.1016/j.molstruc.2025.141317.

(43) Naha, S.; Velmathi, S. A Fluorescence Turn "on-off" Imaging Probe for Sequential Detection of Al3+ and L-Cysteine in HeLa Cells. *Methods* **2024**, *221*, 27–34. https://doi.org/10.1016/j.ymeth.2023.11.009.

(44) Zhao, C.; Asif, M.; Lu, W.; Shuang, S.; Tang, Y.; Dong, C. Al3+ Induced Hydrolysis of Anthraquinone-Based Schiff Base Fluorescent Probe for Determination PPi Ions and Bioimaging. *J. Mol. Liq.* **2024**, *400*, 124507. https://doi.org/10.1016/j.molliq.2024.124507.

(45) Zavalishin, M. N.; Gamov, G. A.; Pogonin, A. E.; Isagulieva, A. K.; Shibaeva, A. V.; Klimovich, M. A.; Morozov, V. N. A New Fluorescent Vitamin B6-Based Probe for Selective and Sensitive Detection Ga3+ Ions in the Environment and Living Cells. *Dye. Pigment.* **2023**, *219*, 111621. https://doi.org/10.1016/j.dyepig.2023.111621.

(46) Goyal, H.; Annan, I.; Ahluwalia, D.; Bag, A.; Gupta, R. Discriminative 'Turn-on' Detection of Al3+ and Ga3+ Ions as Well as Aspartic Acid by Two Fluorescent Chemosensors. *Sensors* **2023**, *23* (4), 1798. https://doi.org/10.3390/s23041798.

(47) Kang, M.; Jiang, S.; Liu, Y.; Wei, K.; Liu, P.; Yang, X.; Pei, M.; Zhang, G. A New "off-on-off" Schiff Base from Quinoline and Thiophene as a Fluorescent Sensor for Sequential Monitoring Ga3+ and Pd2+. *J. Photochem. Photobiol. A Chem.* **2023**, *438*, 114510. https://doi.org/10.1016/j.jphotochem.2022.114510.

(48) Liu, Y.; Wang, H.; Guo, X.; Xing, Y.; Wei, K.; Kang, M.; Yang, X.; Pei, M.; Zhang, G. Two 5-(Thiophene-2-Yl)Oxazole Derived "Turn on" Fluorescence Chemosensors for Detection of Ga 3+ and Practical Applications. *New J. Chem.* **2022**, *46* (21), 10386–10393. https://doi.org/10.1039/D2NJ01672A.

(49) Xing, Y.; Liu, Z.; Li, B.; Li, L.; Yang, X.; Zhang, G. The Contrastive Study of Two Thiophene-Derived Symmetrical Schiff Bases as Fluorescence Sensors for Ga3+ Detection. *Sensors Actuators B Chem.* **2021**, *347*, 130497. https://doi.org/10.1016/j.snb.2021.130497.

(50) Guo, X.; Wu, J.; Han, H.; Xing, Y.; Liu, Y.; Wei, K.; Kang, M.; Yang, X.; Pei, M.; Zhang, G. A Novel Oxazole-Based Fluorescence Sensor towards Ga3+ and PPi for Sequential Determination and Application. *J. Photochem. Photobiol. A Chem.* **2022**, *433*, 114202. https://doi.org/10.1016/j.jphotochem.2022.114202.

(51) Xiang, H.; Wang, T.; Tang, S.; Wang, Y.; Xiao, N. A Novel Hydrazone-Based Fluorescent “off-on-off” Probe for Relay Sensing of Ga3+ and PPi Ions. *Spectrochim. Acta Part A Mol. Biomol. Spectrosc.* **2022**, *267*, 120510. https://doi.org/10.1016/j.saa.2021.120510.

(52) Mujthaba Aatif, A.; Selva Kumar, R.; Joseph, S.; Vetriarasu, V.; Abdul Majeed, S.; Ashok Kumar, S. K. Pyridinecarbohydrazide-Based Fluorescent Chemosensor for In3+ Ions and Its Applications in Water Samples, Live Cells, and Zebrafish Imaging. *J. Photochem. Photobiol. A Chem.* **2023**, *434*, 114257. https://doi.org/10.1016/j.jphotochem.2022.114257.

(53) Yang, M.; Lee, J. J.; Yun, D.; So, H.; Yi, Y.; Lim, M. H.; Lee, H.; Kim, K.-T.; Kim, C. In Vitro and Vivo Application of a Rhodanine-Based Fluorescence Sensor for Detection and Bioimaging of In3+ at Neutral PH. *J. Photochem. Photobiol. A Chem.* **2023**, *434*, 114249. https://doi.org/10.1016/j.jphotochem.2022.114249.

(54) Jayapriya, S.; Ebenazer, A. F.; Sampathkumar, N.; Rajesh, J.; Rajagopal, G. Chromene Carbohydrazide- Schiff Base as a Highly Selective Turn-Off Fluorescence Chemosensor for In3+ Ion and Its Application. *J. Fluoresc.* **2024**. https://doi.org/10.1007/s10895-024-03655-3.

(55) Liu, Y.; Cui, H.; Wei, K.; Kang, M.; Liu, P.; Yang, X.; Pei, M.; Zhang, G. A New Schiff Base Derived from 5-(Thiophene-2-Yl)Oxazole as “off-on-off” Fluorescence Sensor for Monitoring Indium and Ferric Ions Sequentially and Its Application. *Spectrochim. Acta Part A Mol. Biomol. Spectrosc.* **2023**, *292*, 122376. https://doi.org/10.1016/j.saa.2023.122376.

(56) Yun, D.; Chae, J. B.; So, H.; Do, T. N.; Kim, K.-T.; Yi, Y.; Lim, M. H.; Kim, C. Ratiometric Fluorescence In3+ Sensing via In3+-Triggered Tautomerization: Its Applications to Water Samples, Live Cells and Zebrafish. *Dye. Pigment.* **2020**, *183*, 108704. https://doi.org/10.1016/j.dyepig.2020.108704.

(57) Sun, Y.-X.; Zhao, B.; Gao, L.-L.; Sun, Y.; Chen, J.-H.; Deng, Z.-P.; Li, J.-G.; Ji, B.-T. A Naphthalene Based Chemosensor for Dual Channel Recognition of Al3+and Relay Recognition of Fe3+ in Water-Bearing System and Bioimaging in Zebrafish. *J. Mol. Struct.* **2024**, *1306*, 137850. https://doi.org/10.1016/j.molstruc.2024.137850.

(58) Yang, H.-R.; Liu, J.; Wei, T.-B.; Yao, H.; Shi, B.; Lin, Q. Collaboration Strategy-Based Fluorescence Sensor for Efficient Detecting Al3+ and Hg2+. *J. Mol. Struct.* **2024**, *1310*, 138197. https://doi.org/10.1016/j.molstruc.2024.138197.

(59) Liu, Q.; Liu, Y.; Xing, Z.; Huang, Y.; Ling, L.; Mo, X. A Novel Dual-Function Probe for Fluorescent Turn-on Recognition and Differentiation of Al3+ and Ga3+ and Its Application. *Spectrochim. Acta Part A Mol. Biomol. Spectrosc.* **2023**, *287*, 122076. https://doi.org/10.1016/j.saa.2022.122076.

(60) Guo, X.; Han, H.; Xing, Y.; Zhang, G. An Oxazole-Derived Schiff Base as a Multifunctional Fluorescence Sensor towards Al3+ and Ga3+ in Different Media. *J. Mol. Liq.* **2023**, *388*, 122719. https://doi.org/10.1016/j.molliq.2023.122719.

(61) Mishra, S.; Mamidi, P.; Chattopadhyay, S.; Singh, A. K. Economically Viable

Multi-Responsive Probes for Fluorimetric Detection of Trace Levels of Ga3+, Al3+ and PPi in near Aqueous Medium. *J. Photochem. Photobiol. A Chem.* **2023**, *434*, 114225. https://doi.org/10.1016/j.jphotochem.2022.114225.

(62) Zhang, Y.; Li, B. A Multifunctional Selective Fluorescent Chemosensor for Detection of Ga3+, In3+ and Fe3+ in Different Solvents. *J. Mol. Struct.* **2022**, *1250*, 131461. https://doi.org/10.1016/j.molstruc.2021.131461.

(63) Zhu, S.; Yang, L.; Zhao, Y. Ethyl 3-Aminobenzo[b]Thiophene-2-Carboxylate Derived Ratiometric Schiff Base Fluorescent Sensor for the Recognition of In3+ and Pb2+. *J. Fluoresc.* **2024**. https://doi.org/10.1007/s10895-023-03576-7.
